# New Cyclopeptides and Curvularins from Marine-Derived Fungal-Bacterial Symbiont *Aspergillus spelaeus* GXIMD 04541/*Sphingomonas echinoides* GXIMD 04532

**DOI:** 10.3390/md24030111

**Published:** 2026-03-15

**Authors:** Fei-Hua Yao, Jie Yang, Xiao-Yan Li, Shu-Fen Xu, Kai Liu, Zhen-Zhou Tang, Wei-Hui Li, Yong-Hong Liu, Xiang-Xi Yi, Cheng-Hai Gao

**Affiliations:** 1 Faculty of Pharmacy, Institute of Marine Drugs, Guangxi University of Chinese Medicine, Nanning 530200, China; hyywyfh@163.com (F.-H.Y.); jieyang202312@163.com (J.Y.); 13457524492@163.com (X.-Y.L.); 18370712882@163.com (S.-F.X.); kailiu@outlook.com (K.L.); trcstrive2015@126.com (Z.-Z.T.); yonghongliu@scsio.ac.cn (Y.-H.L.); 2 Guangxi Key Laboratory of Marine Drugs, University Engineering Research Center of High-Efficient Utilization of Marine Traditional Chinese Medicine Resources, Guangxi, Guangxi University of Chinese Medicine, Nanning 530200, China; 3 State Key Laboratory for Conservation and Utilization of Subtropical Agro-Bioresources, College of Life Science and Technology, Guangxi University, Nanning 530004, China; lwhlbx@163.com

**Keywords:** fungal-bacterial symbiont, cyclic tetrapeptides, antituberculosis activity, cytotoxicity

## Abstract

Three new cyclic tetrapeptides (nectriatidels A-C, **1**–**3**), two new curvularin analogs (**6** and **7**), and four known compounds (**4** and **5**, **8** and **9**) were isolated from the marine-derived fungal-bacterial symbiont *Aspergillus spelaeus* GXIMD 04541/*Sphingomonas echinoides* GXIMD 04532, which was obtained from *Mauritia arabica* in shallow coastal waters. Their structures were elucidated through NMR spectroscopy and HRESIMS, and their absolute configurations were determined by Marfey’s method and quantum chemical calculations. Compounds **1**–**5** showed moderate amphotericin B (AmB)-potentiating activity against *Candida albicans*. Compounds **7** and **8** exhibited significant activities against *Mycobacterium tuberculosis*, with MIC values of 32 and 16 μg/mL, respectively. Additionally, compounds **7** and **8** exhibited moderate cytotoxicity against human colorectal cancer cell lines DLD-1 and SW480, with IC_50_ values of 25~36 μM. Whole-genome sequencing of *A. spelaeus* revealed a 35.91 Mb assembly encoding 106 biosynthetic gene clusters (BGCs). antiSMASH analysis revealed that 79 of these BGCs (74.5%) displayed no significant similarity to known pathways in the MIBiG database, which is dominated by hybrid clusters, terpene, T1PKS, NRPS, and NRPS-like types. Genomic analysis identified the putative biosynthetic gene clusters for these metabolites and confirmed the fungal host as the predominant producer.

## 1. Introduction

Endosymbiosis is a specialized symbiotic relationship wherein a microbial partner resides intracellularly within its host. As an intimate symbiotic relationship, it is prevalent among fungi. Most fungi harbor bacterial endosymbionts within their hyphae, and these bacteria benefit from a stable niche and nutritional support [1]. In return, these endobacteria significantly enhance the ecological fitness of fungal hosts by contributing to chemical defense, growth promotion, and metabolic supplementation. Through the biosynthesis of bioactive metabolites such as necroximes and rhizoxins, the endosymbionts help their fungal hosts deter predators, resist pathogens, and evade host immune responses [2,3,4]. Beyond defense, they facilitate fungal growth by stimulating hyphal elongation [5] and sporulation [6]. Furthermore, they provide essential metabolic support [7] and help their fungal host cope with nutrient limitations and environmental stressors [7,8]. Given their remarkable biosynthetic versatility, fungal-bacterial symbionts could serve as an underexplored reservoir of structurally unique natural products with considerable pharmaceutical potential.

**Figure 1:**
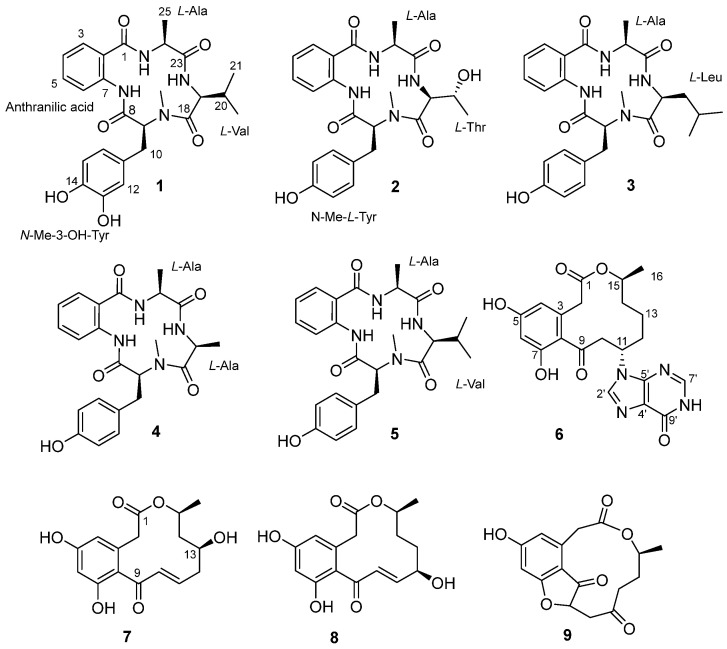
The structure of compounds **1**–**9**.

The ecological context of such interactions was critical in guiding the discovery of new bioactive microbial metabolites. To date, research on fungal-bacterial symbioses have primarily focused on associations from plants and soils, with the fungal phylum Mucoromycota harboring Burkholderia-related bacteria being the most extensively studied [1]. In contrast, marine extreme environments, which exert strong ecological and evolutionary pressures, remain an underexplored source of fungal-bacterial symbionts. Reports of such marine-derived symbionts are still scarce. The first documented example, isolated from deep-sea sediments, was *Spiromastix* sp. SCSIO F190/*Alcaligenes faecalis* SCSIO B001, which produced the broad-spectrum antimicrobial polyketides spiromarmycins [9]. Subsequently, another fungal-bacterial symbiont, *Aspergillus spelaeus* GXIMD 04541/*Sphingomonas echinoides* GXIMD 04532, was isolated from the marine gastropod *Mauritia arabica* collected in shallow coastal waters of southern China. Our preliminary investigation of this fungal-bacterial pair led to the isolation of the polyketide mauritone A and several anti-inflammatory curvularin analogs [10]. As part of our ongoing exploration of this fungi-bacterial symbiont, we conducted a further detailed chemical investigation of its culture extract. This effort resulted in the discovery of five new compounds, including three cyclic tetrapeptide nectriatidels A-C (**1**–**3**), and two curvularin analogs (**6** and **7**), together with four known compounds (**4** and **5**, **8** and **9**) (Figure 1). Herein, we describe the isolation, structural elucidation, bioactivity, and bioinformatic analysis of these compounds.

## 2. Results and Discussion

Compound **1** was obtained as a white powder, and its molecular formula was established as C_25_H_30_N_4_O_6_ by HRESI-MS at *m*/*z* 505.2072 [M + Na]^+^ (calcd for 505.2063), indicating 13 degrees of unsaturation. The ^1^H NMR spectrum of **1** displayed seven aromatic protons, four methines, one methylene, and four methyl signals. ^13^C NMR and DEPT spectra showed 25 carbon signals, including four methyls, one methylene, eleven methines (including seven olefinic carbons), and nine nonprotonated carbons (four carbonyls, five olefinic). Comparison of the NMR data (Table 1) of **1** with those of nectriatidel (**5**) [11] disclosed their resemblance, differing only in that **1** possessed an additional nonprotonated olefinic carbon at C-15, and lacked one aromatic proton, indicating that **1** belonged to cyclopeptide compound.

**Figure 2:**
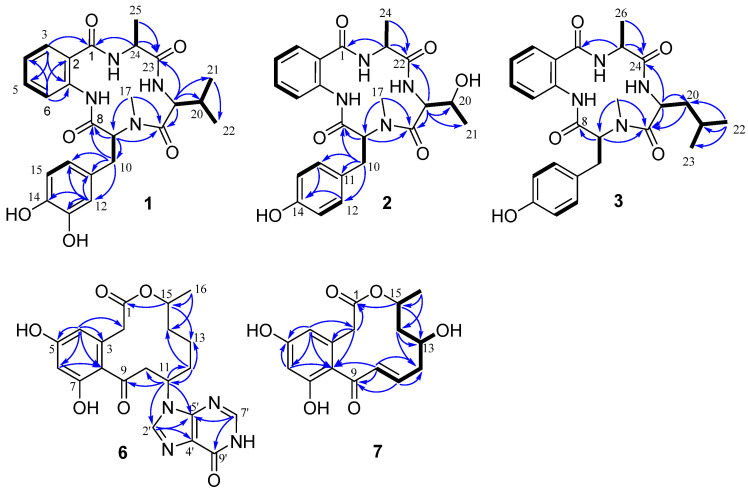
Key COSY and key HMBC correlations of compounds **1**–**3**, **6** and **7**.

Detailed analysis of the HSQC, HMBC, and ^1^H-^1^H COSY spectra (Figure 2) revealed that **1** contained four amino acid residues, including an anthranilic acid (Aad), a *N*-methyl-3-hydroxytyrosine (*N*-Me-3-OH-Tyr), a valine (Val), and an alanine (Ala). The ^1^H-^1^H COSY correlations of H-3 (*δ*_H_ 7.56)/H-4 (*δ*_H_ 7.16)/H-5 (*δ*_H_ 7.48)/H-6 (*δ*_H_ 8.34), together with the HMBC correlations from H-3 to C-1 (*δ*_C_ 172.1), C-5 (*δ*c 132.6) and C-7 (*δ*c 138.1), and from H-6 to C-2 (*δ*c 125.4), C-4 (*δ*c 124.3), and C-7, suggested that **1** possessed an anthranilic acid moiety. The presence of *N*-methyl-3-hydroxytyrosine moiety was revealed by COSY correlations of H-9 (*δ*_H_ 4.11)/H-10 (*δ*_H_ 3.28, 3.08), H-15 (*δ*_H_ 6.70)/H-16 (*δ*_H_ 6.54), and the HMBC correlations from H-9 (*δ*_H_ 4.11) to C-8 (*δ*c 170.2), C-17 (*δ*c 40.7) and C-18 (*δ*c 173.0), from H_2_-10 (*δ*_H_ 3.28, 3.08) to C-8, C-11 (*δ*c 131.0), C-12 (*δ*c 121.7), and C-16 (*δ*c 117.5), from H-12 (*δ*_H_ 6. 69) to C-10 (*δ*c 33.9), C-11, C-13 (*δ*c 146.6), C-14 (*δ*c 145.2) and C-16, and from H_3_-17 (*δ*_H_ 2.95) to C-9 (*δ*c 70.3) and C-18. In addition, HMBC correlations from H-19 (*δ*_H_ 4.49) to C-18 (*δ*c 173.0), C-20 (*δ*c 31.2), C-21 (*δ*c 20.0) and C-23 (*δ*c 176.1), and from H-21 (*δ*_H_ 0.94) to C-22 (*δ*c 18.7), together with COSY correlations of H-19/H-20/H-21/H-22 (*δ*_H_ 0.89), indicated the existence of a valine residue. Further HMBC correlations from H-24 (*δ*_H_ 4.13) to C-1, C-23 (*δ*c 176.1), from H_3_-25 (*δ*_H_ 1.46) to C-23, and COSY correlations of H-24 (*δ*_H_ 4.13) with H-25 (*δ*_H_ 1.46) revealed an alanine fragment. Furthermore, the HMBC correlations from -NCH_3_ of *N*-methyl-3-hydroxytyrosine to C-18 of Val, from H-19 of Val to C-23 of Ala, and from H-24 of Ala to C-1 of Aad, suggested the sequence of the amino acid residues was Aad-*N*-Me-3-OH-Tyr-Val-Ala.

**Figure 3:**
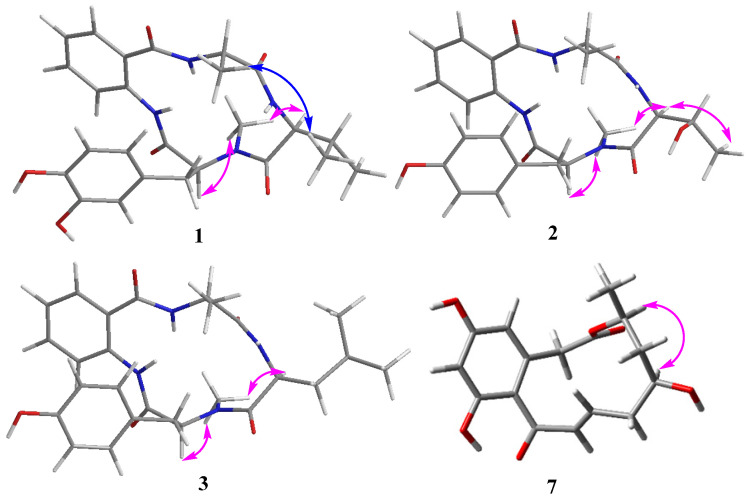
Key NOESY correlations of compounds **1**–**3** and **7**.

The amino acid residues were identified as *L*-Val and *L*-Ala by Marfey’s method (see Section 3.4 Materials and Methods, Figure S61). However, the configuration of the *N*-methyl-3-hydroxytyrosine residue could not be determined due to the unavailability of the corresponding standard amino acid for comparison. In the NOESY spectrum, key NOE correlations of H_3_-17 with H-9 and H-19 indicated their cofacial *α*-orientations, while the NOE correlation of H_3_-22 with H_3_-25 suggested these methyl groups on the opposite face (Figure 3). Therefore, the configuration of the *N*-methyl-3-hydroxytyrosine residue was defined as *L*-*N*-Me-3-OH-Tyr. Thus, the structure of **1** was elucidated as shown and named nectriatidel A.

Compound **2** was isolated as a white powder with a molecular formula of C_24_H_28_N_4_O_6_ determined by NMR and HRESI-MS data. A thorough analysis of the NMR data revealed that the structure of **2** closely resembled that of nectriatidel (**5**) [11], differing by the absence of one methine and one methyl, and the presence of an additional oxygenated methine group at (*δ*_H_ 3.94, m; *δ*c 68.4) in **2**. The oxygenated methine H-20 (*δ*_H_ 3.94) exhibited ^1^H-^1^H COSY correlations with H-19 (*δ*_H_ 4.73) and H-21 (*δ*_H_ 1.16), along with the HMBC interactions from H-19 to C-18, C-20, C-21, and C-22, confirming the existence of a threonine residue. Further HMBC correlations from -NCH_3_ (*δ*_H_ 2.91) of *N*-methyl-tyrosine to C-18 of Thr, from H-19 of Thr to C-22 (*δ*c 176.5) of Ala, and from H-23 of Ala to C-1 of Aad, suggested an amino acid sequence of Aad-*N*-Me-Tyr-Thr-Ala in **2**. The relative configuration of **2** was consistent with that of **1**, as deduced from NOESY correlations of H_3_-17 with H-9 and H-19, and of H-19 with H_3_-21 for *α*-orientation (Figure 3). Thereafter, the configuration of the amino acid residues in **2** was conclusively assigned to *L*-*N*-Me-Tyr, *L*-Thr, and *L*-Ala by Marfey’s method and HPLC analysis (see Figure S62).

Compound **3** was isolated as a white powder, and its molecular formula was determined to be C_26_H_32_N_4_O_5_ based on its HRESI-MS data (*m*/*z* 503.2272 [M + Na]^+^). Extensive NMR data analysis revealed that **3** shared a similar structure with **5** [11]. The key difference was the presence of an additional methylene signal, indicating that the valine residue in **5** was replaced by leucine in **3**. This conclusion was further supported by key HMBC correlations from H-19 (*δ*_H_ 4.96) to C-18 (*δ*c 173.1), C-20 (*δ*c 41.7) and C-24 (*δ*c 175.8), from H-20 (*δ*_H_ 1.61, 1.40) to C-18, and from H-22 (*δ*_H_ 0.96) to C-20, C-21 (*δ*c 26.0) and C-23 (*δ*c 22.8), together with the COSY correlations of H-19/H-20/H-21/H-22/H-23. Further 2D NMR analysis established the amino acid sequence of **3** as Aad-*N*-Me-Tyr-Leu-Ala. The consistency of the key NOESY correlations (H_3_-17/H-9 and H_3_-17/H-19) between compounds **3** and **2** supported the assignment of an identical relative configuration. Finally, the configuration of amino acid residues in **3** was identified as *L*-*N*-Me-Tyr, *L*-Val, and *L*-Leu through Marfey’s method (Figure S63).

Compound **6** was obtained as yellow oil with a molecular formula of C_21_H_22_N_4_O_6_ based on HRESI-MS analysis. Its ^1^H NMR spectrum revealed the presence of four aromatic proton signals at *δ*_H_ 8.20 (s, H-2′), 8.03 (s, H-7′), 6.26 (d, *J* = 2.3 Hz, H-6), 6.24 (d, *J* = 2.3 Hz, H-4), one oxygenated methine proton at *δ*_H_ 4.87, one nitrogenated methine at *δ*_H_ 5.07 (dd, *J* = 9.5, 4.8 Hz, H-11), and one methyl at 1.16 (d, *J* = 6.2 Hz, H_3_-16). The ^13^C NMR and DEPT spectra displayed eighteen carbon signals, including one methyl, four methylenes, six methines, and seven quaternary carbons. Further analysis of HSQC and HMBC spectra identified three additional carbon signals, including one methylene at *δ*_C_ 51.5 and two quaternary carbons *δ*_C_ 119.1 and 124.3, which were not detected in the ^13^C NMR spectrum. The presence of two singlets at *δ*_H_ 8.20 (1H, s) and 8.03 (1H, s) in the ^1^H NMR spectrum, along with four nitrogen atoms as indicated by HRESI-MS, suggests the possible existence of a purine moiety in **6**. A comparison of the NMR data (Table 2) of **6** with those of (11*S*,15*S*)-11-adeninecurvularin [12] revealed significant similarities, with only differences in chemical shifts. These findings indicated that **6** was a curvularin-type compound, specifically representing a hybrid of curvularin and purine. This deduction was supported by key HMBC correlations from H-2′ (*δ*_H_ 8.22) to C-4′ (*δ*_C_ 125.0) and C-5′ (*δ*_C_ 150.1), from H-7′ (*δ*_H_ 8.04) to C-5′ and C-9′(*δ*_C_ 159.3), and from H-11 to C-9 (*δ*_C_ 203.8), C-10 (*δ*_C_ 51.5), C-12 (*δ*_C_ 33.3), C-2′(*δ*_C_ 141.0) and C-5′. These correlations confirmed the attachment of the hypoxanthine moiety at C-11 in **6**. Thus, the planar structure of **6** was proposed as depicted.

**Figure 4:**
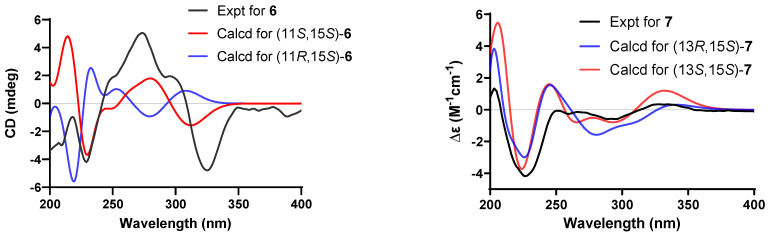
Comparison of the experimental and calculated ECD spectra of **6**–**7** in MeOH.

Due to the significant loss during transfer and the limited sample quantity, the absolute configuration of **6** was ultimately determined through quantum chemical ECD calculations and biosynthetic pathway analysis. The configuration of C-15 was assigned as 15*S* based on the total synthesis of published curvularins [13] and the shared biogenetic pathway for curvularin derivatives [10]. In addition, the absolute configuration of C-11/C-15 in **6** was determined as 11*S*,15*S* by comparing its experimental ECD spectrum with that of (11*S*,15*S*)-11-adeninecurvularin [12]. To further confirm the absolute configuration, ECD spectra were calculated for isomers (11*R*,15*S*)-**6** and (11*S*,15*S*)-**6**. The calculated ECD spectrum of (11*S*,15*S*)-**6** matched well with the experimental spectrum of **6** (Figure 4). Furthermore, we performed NMR chemical shift calculations for both isomers. The calculated ^13^C NMR chemical shifts for (11*S*,15*S*)-**6** were in good agreement with the experimental data, with a high R^2^ value of 0.9940. In addition, the DP4+ results unambiguously identified (11*S*,15*S*)-**6** isomer as the likely real structure, with a 100% DP4+ probability (Figure S66). Thus, the structure of **6** was elucidated and designated as (11*S*,15*S*)-11-hypoxanthinecurvularin.

Compound **7** had the molecular formula C_16_H_18_O_6_ as deduced from its HRESI-MS and NMR data (Table 2), identical to that of (10*E*,15*R*)-13-hydroxy-10,11-dehydrocurvularin [14]. Their ^1^H NMR spectra were closely matched, with only differences in the chemical shifts (Figure S39). Further analysis of the HSQC, COSY, and HMBC spectrum of **7** confirmed its planar structure, which was identical to that of (+)-(10*E*,15*R*)-13-hydroxy-10,11-dehydrocurvularin. However, compound **7** showed a negative Cotton effect around 230 nm in the CD spectrum (Figure 4), which was opposite to the positive Cotton effect reported for (+)-(10*E*,15*R*)-13-hydroxy-10,11-dehydrocurvularin [230 (+5.49)]. Consistent with this, the specific rotation of **7** {$[\alpha {]}_{D}^{25}$ –12.0 (*c* 0.05, MeOH)} was also opposite in sign to that of the known compound {$[\alpha {]}_{D}^{25}$ +126.5 (*c* 0.29, EtOH)} [14]. These data indicated that the two compounds were stereoisomers, allowing the C-15 configuration of **7** to be deduced as 15*S*. The relative configuration of **7** was determined by analysis of the NOESY spectrum (see Figure S37 in the Supplementary Material), in which the correlation between H-13 and H-15 indicated their cofacial *α*-orientation. We found that the calculated ECD spectra of (13*S*,15*S*)-**7** and (13*R*,15*S*)-**7** were similar to the experimental CD spectrum (Figure 4), and could not unambiguously determine the absolute configuration of **7**. Subsequently, two possible isomers were subject to quantum chemical calculations of NMR chemical shift. The DP4+ probability analysis supported (13*S*,15*S*)-**7** as the likely real structure, with a 100% DP4+ probability (Figure S69). In addition, linear regression of experimental versus calculated 13C NMR shifts yielded an R^2^ of 0.9967 for (13*S*,15*S*)-**7**, compared to 0.9935 for (13*R*,15*S*)-**7** (Figure S73), suggesting that the calculated 13C NMR chemical shifts of (13*S*,15*S*)-**7** showed good agreement with the experimental data. Thus, the structure of **7** was established as (13*S*,15*S*)-13-hydroxy-10,11-dehydrocurvularin.

Compound **8** had the same molecular formula C_16_H_18_O_6_ as **7** based on its HRESI-MS and NMR data. A thorough analysis of the NMR data revealed that the planar structure of **8** was identical to that of 12-hydroxy-10,11-*trans*-dehydrocurvularin [13] and (+)-(10*E*,15*R*)-12-hydroxy-10,11-dehydrocurvularin [14] (Table S8 and Figure S40). Compound **8** displayed a negative Cotton effect around 220 nm in the CD spectrum (Figure S68), which was identical to that of compound **7**. Although no CD data were reported for the known compound 12-hydroxy-10,11-*trans*-dehydrocurvularin, the specific rotation of **8** {$[\alpha {]}_{D}^{26}$ –24.0 (*c* 0.05, MeOH)} shared the same sign as that of the known compound {$[\alpha {]}_{D}^{26}$ –49.5 (*c* 0.86, EtOH)} [13], supporting the assignment of the C-15 configuration in **8** as 15*S*. However, the stereochemistry at C-12 remains unreported in the literature. To determine the absolute configuration of **8**, two possible isomers were subject to chemical calculations. Although the configuration at C-12 could not be unambiguously assigned by ECD calculation (Figure S71), the calculated 13C NMR chemical shifts of (12*R*,15*S*)-**8** were in good agreement with the experimental data, with a 90.68% DP4+ probability (Figure S72). Thus, the structure of **8** was established as (10*E*,12*R*,15*S*)-12-hydroxy-10,11-dehydrocurvularin.

Other known compounds were also isolated and identified as cyclo(Ala-NMeTyr-Ant-Ala) (**4**) [15], nectriatidel (**5**) [11], and ent-curvulone A (**9**) [16] by comparison of their spectroscopic data with those reported in the literature. Cyclic peptides are a major class of structurally diverse natural products with a broad range of biological activities [17], which has attracted growing interest from the pharmaceutical industry. Among them, cyclic tetrapeptides are particularly notable for potent and diverse bioactivities, including antitumor, cytotoxic, antiviral, antibacterial, antifungal, and antibiotic-potentiating activities [11,18]. Previous studies have reported that compound **5** potentiated the activity of AmB against *C*. *albicans* in a dose-dependent manner [11,15]. In this study, we tested the AmB-potentiating activity of nectriatidel derivatives against *C*. *albicans*. The minimum inhibitory concentration (MIC) value of AmB alone against *C. albicans* was 1.0 μg/mL (Table 3), whereas compounds **1**–**5** showed no activity at 50 μg/mL. Compounds **3** and **5** decreased the MIC value of AmB from 1.0 μg/mL to 0.125 μg/mL, with 8-fold potentiation at the concentration of 32 μg/mL. This effect was weaker than that reported in previous studies [11,15]. Compounds **1**, **2** and **4** exhibited moderate AmB-potentiating activity, weaker than compounds **3** and **5**. For compound **4**, replacement of Valine with Alanine at the third amino acid position led to a 4-fold potentiation of AmB activity. In contrast, for compound **2**, the presence of threonine at the same position had only a minimal effect on the AmB-potentiating activity. This highlights the importance of this residue for AmB potentiation.

We also evaluated the antitubercular activity of compounds **1**–**5** and **7**–**9**. Compounds **7** and **8** showed moderate anti-*M*. *tuberculosis* activity, with MIC values of 32 and 16 μg/mL, respectively (Figure S74). However, other compounds did not exhibit antitubercular activity at 50 μg/mL. In addition, we also evaluated the cytotoxicity of compounds **1**–**5**, **7**–**9** toward human colorectal cancer cell lines and prostate cancer cell lines. Only compounds **7** and **8** exhibited moderate cytotoxicity against human colorectal cancer cell lines DLD-1 and SW480, other compounds exhibited no cytotoxicity toward HT29, MCF7, and KTC cell lines at 40 μM. Compound **7** exhibited moderate cytotoxicity against human colorectal cancer DLD-1, SW480, and B16F10 cell lines with IC_50_ values of 36, 25, and 35 μM (Figure S75), respectively. Compound **8** exhibited moderate cytotoxicity against human colorectal cancer DLD-1 and SW480 cell lines with IC_50_ values of 30 and 36 μM, respectively. Compounds **1**–**5**, **7**–**9** did not exert cytotoxicity to prostate cancer cell lines 22Rv1 and PC3 at 10 μM (Figure S75). Furthermore, we assessed the acetylcholinesterase and α-glucosidase activities of these compounds **1**–**5**, **7**–**9**, but found no activities at the test concentration.

In this work, a chemical investigation of the marine gastropod-derived fungal-bacterial symbiont *A*. *spelaeus* GXIMD 04541/*S*. *echinoides* GXIMD 04532 led to the isolation of five cyclopeptides (**1**–**5**), and four curvularin analogs (**6**–**9**), expanding the diversity and complexity of marine natural products. However, the biosynthetic origins of these metabolites remain to be elucidated in this symbiotic system. We attempted to isolate pure cultures of the fungus and its endobacterium using various methods but failed, indicating an intimate symbiotic relationship. Given the low abundance of bacteria within fungal mycelia and spores, we obtained the fungal genome sequence by bioinformatically filtering out its endobacterial sequences.

The genome assembly of *A*. *spelaeus* GXIMD 04541 comprises 358 scaffolds totaling 35.91 Mbp with a GC content of 52.6% (Table S14). Functional annotation predicted 11,886 genes, 199 *tRNA* genes, and 44 *rRNA* genes. Genome mining of *A*. *spelaeus* GXIMD 04541 using antiSMASH v.8.0.4 with default parameters predicted a total of 106 putative biosynthetic gene clusters associated with secondary metabolite production (Table S15). These clusters encompassed a diverse range of biosynthetic types. Hybrid clusters incorporating multiple biosynthetic systems (*n* = 27), such as NRPS-T1PKS, NRPS-terpene, T1PKS-terpene, were the dominant clusters, followed by terpene clusters (*n* = 23), type I polyketide synthase (T1PKS) clusters (*n* = 15), nonribosomal peptide synthetase (NRPS) clusters (*n* = 13), and NRPS-like clusters (*n* = 9). Minor but chemically informative categories comprised 7 indole clusters, 3 terpene precursor clusters, one betalactone cluster, one T3PKS cluster, and one fungal-CDPS cluster. Notably, 79 clusters (74.5%) lacked significant similarity to any known pathways in the MIBiG database, indicating extensive cryptic biosynthetic potential dominated by terpene, NRPS, T1PKS, NRPS-like, terpene and hybrid clusters.

Five nectriatidel derivatives identified in this study are cyclic tetrapeptides that incorporate an anthranilic acid unit. Structurally analogous peptides have been reported from the soil-derived fungus *Nectriaceae* sp. and the marine-derived fungus *Aspergillus terreus* SCSGAF0162, suggesting that the fungal host is likely the putative producer in our symbiont system. Nonribosomal peptide synthetases (NRPS) are large, complex multidomain enzymes responsible for the biosynthesis of cyclic peptides [19]. Given the unique nature of an anthranilic acid adenylating domain, we performed bioinformatic analysis on the adenylation (A) domains of the NRPS. By comparison, the deduced NRPS domain architectures to the chemical structure of nectriatidels, an NRPS cluster located on scaffold 4 Region 3 from 374,218 to 448,510 bp, attracted our attention. This NRPS gene *g2525.t1* encoded three modules for the biosynthesis of an Aad-Ala-Val fragment, along with three outside modules (Figure S76). In addition, the adjacent gene *g2526.t1* encoded a single module that was possibly implicated in the biosynthesis of a Tyr or OH-Tyr residue. Another NRPS gene *g10543.t1* encoded three modules predicted to assemble an Aad-Ala-Ala fragment. Although the encoded assembly line is not fully co-linear with the tetrapeptide backbone, as three NRPS modules would typically yield a tripeptide. Nevertheless, the presence of a specific anthranilic acid adenylating domain supports the capacity for anthranilate incorporation. Taken together, these genomic features indicate that the fungal host possesses a functional NRPS cluster for cyclotetrapeptide biosynthesis, thereby designating *A. spelaeus* as the putative producer of the nectriatidel analogs.

The resorcylic acid lactones (RALs) are a class of secondary metabolites produced by various fungi, marine organisms, and plants. They are predominantly isolated from fungal genera, including *Ilyonectria*, *Fusarium*, *Paecilomyces*, *Penicillium*, and *Aspergillus*, among others [20,21], and exhibit a wide range of bioactivities. Given that RALs are primarily of fungal origin, compounds **6**–**9** are most likely biosynthesized by the host *A*. *spelaeus* in this symbiotic system. Previous studies reported that RAL biosynthesis is catalyzed by two core polyketide synthase (PKS) proteins, a highly reducing PKS (hrPKS) and a nonreducing PKS (nrPKS) [22,23]. In the genome of *A*. *spelaeus*, we identified a putative RALs biosynthetic gene cluster. This cluster comprises 23 genes and encodes all five core enzymes essential for RALs biosynthesis, including two polyketide synthases (hrPKS, nrPKS), a dehydrocurvularin biosynthesis regulator, a cytochrome P450, and a major facilitator superfamily (MFS) transporter (Figure S78). The high similarity of these core genes to those characterized in *Aspergillus terreus* and other documented RALs producers [22,23,24], strongly supports the functional role of this cluster. Therefore, we designated this cluster as the most likely candidate responsible for RAL biosynthesis, and confirmed the fungal host as the producer of the RAL derivatives **6**–**9**. Compound **6**, an unusual Michael adduct identified in this study, possesses distinct structural features. Its chirality suggests an enzyme-mediated biosynthetic origin within microbial metabolism, warranting further exploration.

Cyclic tetrapeptides (**1**–**5**) isolated in this study had no antimicrobial activity or cytotoxicity but exhibited moderate AmB-potentiating activity under our assay conditions. This suggests that, in an ecological context, they may play a specialized role in the host defense system, potentially targeting non-microbial threats. In contrast, the curvularin derivatives (**7** and **8**) exhibited antitubercular and cytotoxic effects, positioning them as potential key chemical defense agents in the fungal-bacterial symbiotic system. Building on the distinct but complementary bioactivity profiles of these two compound classes, we hypothesize a potential synergistic interplay within the symbiosis. The curvularin derivatives could serve as the frontline antimicrobial defense, while the cyclic tetrapeptides might function as accessory molecules that enhance this defense. This proposed synergism not only offers a plausible ecological explanation for the co-production of these metabolites but also highlights a promising direction for future research aimed at evaluating their combined biological effects. Future investigations into the potential synergistic effects between these peptides and polyketides are warranted to validate this ecological hypothesis.

## 3. Materials and Methods

### 3.1. General Experimental Procedures

The general procedures were described previously [10]. 1D NMR and 2D NMR spectra were acquired using an Avance III 500 NMR spectrometer (Bruker Corporation, Billerica, MA, USA) with TMS as an internal standard. HR-ESI-MS data were recorded on a Xevo G2-XS QTOF HRMS spectrometer (Waters, Milford, MA, USA). Semipreparative reversed-phase HPLC was performed on a Waters 2695 HPLC system (Waters Technology Shanghai Co., Ltd, Shanghai, China) equipped with a YMC-Pack ODS-A C18 column (10 × 250 mm, 10 μm, YMC, Tokyo, Japan). UV spectra were measured on an Evolution 350 spectrometer (Thermo Fisher Scientific Inc., Waltham, MA, USA). Optical rotations were recorded on an Insmark IP-digi300 polarimeter (Shanghai Insmar Instrument Technology Co., Ltd., Shanghai, China). CD spectra data were measured using a JASCO J-1500 circular dichroism spectrophotometer (JASCO, Easton, PA, Tokyo, Japan). OD values were recorded on a PerkinElmer VICTOR NIVO multimode plate Reader (PerkinElmer Management Shanghai Co., Ltd., Shanghai, China). Column chromatography was performed with silica gel (200–300 mesh, Qingdao Ocean Chemical Co., Ltd., Qingdao, China), ODS (40–63 μm, YMC, Tokyo, Japan), and Sephadex LH-20 (GE Healthcare, Chicago, IL, USA). The silica gel GF254 (10–40 μm) used for TLC was supplied by Qingdao Ocean Chemical Co., Ltd., Qingdao, China. Cell Counting Kit-8 was obtained from Biyuntian Biotechnology Co., Ltd, Shanghai, China.

### 3.2. Strain Material and Identification

The sample *Mauritia arabica* was collected at a depth of approximately 30 m in the offshore waters near Nanji Village, Leizhou, Zhanjiang City, Guangdong Province, China (109°55′ E, 20°13′ N) on 18 March 2023. This species typically inhabits rocky substrates in the lower intertidal zone. The identification was confirmed by Dr. Liu Xinming of Guangxi University of Chinese Medicine. A fungal-bacterial symbiont was isolated from the fleshy tissue of *Mauritia arabica*. The fungal strain was identified as *Aspergillus spelaeus* based on the ITS sequence, and the endobacterial strain GXIMD 04532 was identified as *Sphingomonas echinoides* based on the 16S rRNA sequence. The fungal-bacterial symbiont *Aspergillus spelaeus* GXIMD 04541/*Sphingomonas echinoides* GXIMD 04532 was stained with SYTO9, a fluorescent dye specific to bacteria. Using laser confocal microscopy [25], green fluorescence signals were observed in both the mycelium and spores, indicating the presence of endosymbiotic bacteria. This result confirmed the existence of bacterial endosymbionts within the fungal host. The strain was deposited in the Institutes of Marine Drugs, Guangxi University of Chinese Medicine.

### 3.3. Fermentation, Extraction and Isolation

The fungal-bacterial symbiont was cultured in 200 mL of potato dextrose broth (PDB) at 28 °C on a rotary shaker (180 rpm) for three days to obtain the seed culture. For large-scale fermentation, 150 Erlenmeyer flasks (1000 mL) were used, with each containing 80.0 g of rice, 0.4 g of yeast extract, 0.4 g of glucose, 3.6 g of sea salt, and 120 mL of water. The rice solid media were supplemented with a 5 mL seed broth and incubated at room temperature for 25 days. The fermentation was then repeatedly extracted with ethyl acetate (EtOAc) four times, yielding 197.3 g of crude extract. The extract was fractionated on silica gel G using CHCl_3_-CH_3_OH (100:0, 98:2, 95:5, 85:15, 8:2, 7:3, 0:100, *v*/*v*) as eluent, producing five fractions (Fr 1-Fr 5). Fr 3 (30.5 g) was subjected to reversed-phase silica gel column chromatography with H_2_O/MeOH (10–100%) to give 6 fractions (Fr 3.1-Fr 3.6). Fr 3.3 was further separated by reversed-phase silica gel column chromatography with H_2_O/CH_3_CN to give 7 fractions (Fr 3.3.1-Fr 3.3.7). Fr 3.3.3 was purified by semipreparative HPLC using CH_3_CN/H_2_O (30:70, *v*/*v*, 2 mL/min) to yield compounds **1** (3.2 mg, *t*_R_ = 24.72 min), **2** (3.8 mg, *t*_R_ = 25.17 min), and **4** (2.9 mg, *t*_R_ = 25.99 min). Fr 3.3.4 was subjected to semipreparative HPLC (CH_3_OH/H_2_O, 70: 30, 2 mL/min) to afford compounds **3** (2.5 mg, *t*_R_ = 28.16 min) and **5** (12.7 mg, *t*_R_ = 26.80 min). Fr 3.3.5 was separated by semipreparative HPLC with MeOH-H_2_O (48:52, *v*/*v*, 2 mL/min) to give compounds **6** (1.6 mg, *t*_R_ = 25.70 min). Fr 3.4 was purified by semipreparative HPLC (CH_3_OH/H_2_O, 30:70, *v*/*v*, 2 mL/min) to yield compounds **7** (4.7 mg, *t*_R_ = 26.00 min), **8** (5.5 mg, *t*_R_ = 28.46 min) and **9** (6.6 mg, *t*_R_ = 51.90 min).

Nectriatidel A (**1**): white powder; $[\alpha {]}_{D}^{26}$– 80.08 (*c* 0.05, MeOH); UV (MeOH) *λ*_max_ (log *ε*) 209 (4.37), 252 (3.94), 286 (3.61) nm; ^1^H and ^13^C NMR data see Table 1; HR-ESIMS: *m*/*z* 505.2072 [M + Na]^+^ (calcd for C_25_H_30_NaN_4_O_6_^+^, 505.2063).

Nectriatidel B (**2**): white powder; $[\alpha {]}_{D}^{26}$– 76.08 (*c* 0.05, MeOH); UV (MeOH) *λ*_max_ (log *ε*) 220 (4.36), 252 (4.01), 286 (3.52) nm; ^1^H and ^13^C NMR data see Table 1; HR-ESIMS: *m*/*z* 491.1907 [M + Na]^+^ (calcd for C_24_H_28_NaN_4_O_6_^+^, 491.1907).

Nectriatidel C (**3**): white powder; $[\alpha {]}_{D}^{26}$– 60.04 (*c* 0.05, MeOH); UV (MeOH) *λ*_max_ (log *ε*) 220 (4.33), 252 (3.97), 286 (3.53) nm; ^1^H and ^13^C NMR data see Table 1; HR-ESIMS: *m*/*z* 503.2272 [M + Na]^+^ (calcd for C_26_H_32_NaN_4_O_5_^+^, 503.2271).

(11*S*,15*S*)-11-(9*H*-purin-6-ol)-curvularin (**6**): yellow oil; $[\alpha {]}_{D}^{26}$– 50.98 (*c* 0.10, MeOH); UV (MeOH) *λ*_max_ (log *ε*) 240 (3.71), 270 (3.52), 303 (3.25) nm; ECD (2.35 mM, CH_3_OH) *λ*_max_ (Δ*ε*) 218(−0.19), 229 (−0.48), 273 (+0.62), 325 (−0.59) nm; ^1^H and ^13^C NMR data see Table 2; HR-ESIMS: *m*/*z* 427.1636 [M + H]^+^ (calcd for C_21_H_23_N_4_O_6_^+^, 427.1618).

(+)-(10*E*,13*S*,15*S*)-13-Hydroxy-10,11-dehydrocurvularin (**7**): white amorphous powder; $[\alpha {]}_{D}^{26}$– 12.00 (*c* 0.05, MeOH); UV (MeOH) *λ*_max_ (log *ε*) 207 (4.12), 290 (3.45) nm; ECD (1.44 mM, CH_3_OH) *λ*_max_ (Δ*ε*) 203 (+1.35), 226 (−4.18), 251 (−0.065), 292 (−0.57) nm; ^1^H and ^13^C NMR data see Table 2; HR-ESIMS: *m*/*z* 347.1107 [M + Na + H_2_O]^+^ (calcd for C_16_H_20_O_7_Na^+^, 347.1107).

(10*E*,12*R*,15*S*)-12-Hydroxy-10,11-dehydrocurvularin (**8**): white amorphous powder; $[\alpha {]}_{D}^{26}$ –24.04 (*c* 0.05, MeOH); ECD (1.44 mM, CH_3_OH) *λ*_max_ (Δ*ε*) 214 (−4.57), 224 (−4.78), 257 (0.74), 313 (−0.24), 335 (0.48) nm; ^1^H and ^13^C NMR data see Table S8; HR-ESIMS: *m*/*z* 347.1106 [M + Na + H_2_O]^+^ (calcd for C_16_H_20_O_7_Na^+^, 347.1107).

### 3.4. Determination of the Absolute Configurations of Amino Acid Residues of ***1***–***5***

The sample hydrolysis and derivatization procedures were the same as those previously described [26]. Briefly, compounds **1**–**5** (0.5 mg, each) were hydrolyzed and then derivatized with Marfey’s reagent. The dried mixture was dissolved in MeOH and analyzed by HPLC (Waters 2695 HPLC system). Standard amino acids (*D/L*-Ala, *D/L*-Leu, *D/L*-Val, *D/L*-Thr, and *L*-*N*-Me-Tyr) underwent the same derivatization process. The retention times of the FDAA derivatives of **1**–**5** and the standard were compared with those of the standard amino acids (see Supplementary Materials).

### 3.5. Computational Methods

The theoretical calculations of compounds **6** and **7** were performed using Gaussian 09. The conformers were optimized by the time-dependent DFT calculations at B3LYP/6-31G (d) level with Polarized Continuum Model (PCM) in MeOH. The ECD calculations of **6**, **7** and **8** were conducted at the wb97xd/def2tzvp and m062x/def2tzvp level of theory, respectively. The experimental and calculated CD spectra were compared using the software SpecDis 1.70.1, with ECD spectra generated by applying a Gaussian band shape (0.20–0.30 eV half-width). The 13C NMR calculations of **6** and **7** were done with the GIAO method at the mPW1PW91/6-311G (d,p) level with PCM. Finally, the DP4+ probability calculations were completed using the Excel template provided by the Sarotti group [27].

### 3.6. Antifungal Activity of Drug Combinations Against C. albicans

Compounds **1**–**5** were evaluated for antifungal activity against *C*. *albicans* using the broth microdilution method in 96-well microplates (Corning, Kennebunk, ME, USA). *C. albicans* was first cultured on Sabouraud Dextrose agar at 35 °C for 24 h. A single colony was then inoculated into Sabouraud Dextrose Broth (SDB) and incubated at 35 °C for 12 h with shaking (180 rpm). The resulting culture was adjusted to a 0.5 McFarland standard and then diluted 100-fold in fresh SDB to obtain the final working inoculum. Compounds **1**–**5** and AmB were dissolved in 100% dimethyl sulfoxide (DMSO). In the first assay for the antifungal activity, each well was filled with 100 μL of diluted inoculum, 99 μL of SDB, and 1 μL of either the test compounds (final concentration: 100, 50 μg/mL) or AmB (final concentration: 0.125 to 8.0 μg/mL) in a 96-well microplate. The plates were incubated at 35 °C for 24 h~48 h.

For the antifungal potentiation assay, each well contained 100 μL diluted inoculum, 98 μL SDB, 1 μL of amphotericin B (final concentration: 0.0313 to 1.00 μg/mL), and 1 μL of compounds **1**–**5** (final concentration: 0, 2, 4, 8, 16, 32 μg/mL). Plates were incubated at 35 °C for 48 h. After incubation, the MIC of AMB, alone and in combination, was determined as the lowest concentration completely inhibiting visible fungal growth (a clear well), compared to the growth control.

### 3.7. Antitubercular Activity

The antitubercular activity of compounds **1**–**5** and **7**–**9** was determined by the REMA (Resazurin Microtiter Assay) method [28]. Briefly, the *Mycobacterium tuberculosis* H37Rv strain was cultured in Middlebrook 7H9 broth. Upon reaching a logarithmic growth phase, the bacterial suspension was adjusted to 1 × 10^6^ CFU/mL. For initial screening, all compounds were tested at a final concentration of 50 μg/mL. For active compounds **7** and **8**, two-fold serial dilutions were prepared in 96-well plates to achieve final concentrations ranging from 32 to 0.0625 μg/mL. Specifically, 100 μL of each compound solution (64 μg/mL in culture medium) was added to the first well and serially diluted across the plate. Then, 100 μL of bacterial suspension was added to each well. Plates were incubated at 37 °C and 5% CO_2_ for 7 days, with Rifampicin and culture medium as positive and negative controls, respectively. After incubation, 30 μL of 0.2% resazurin solution was added, and the plate was further incubated in the dark for 24 h. The MIC value was determined visually as the lowest concentration that completely inhibited the reduction in resazurin, leaving the well blue, indicating inhibition of bacterial growth.

### 3.8. Cytotoxicity Assay

The cytotoxicity of compounds **1**–**5** and **7**–**9** against human colorectal cancer cell lines (DLD-1 and SW480), and cell lines of mouse cutaneous melanoma B16F10 (all obtained from Guangxi Scientific Research Center of Traditional Chinese Medicine, Guangxi University of Chinese Medicine, Nanning, China) was evaluated in vitro using the Cell Counting Kit-8 (CCK-8) assay [29]. Briefly, the test compounds were dissolved in DMSO and subsequently diluted with culture medium to prepare a series of concentrations. Cells were seeded into 96-well microtiter plates (5 × 10^3^ cells per well), and incubated at 37 °C and 5% CO_2_ for 24 h. Then, cells were treated with the serially diluted compounds for 48 h. Cell viability was determined by CCK-8 assay, using cisplatin and 0.1% DMSO as positive and negative controls, respectively. Compounds **1**–**5** and **7**–**9** were also tested for cytotoxicity against prostate cancer cell lines 22Rv1 and PC3 through the MTT method [30], with doxorubicin as the positive control. IC_50_ values were calculated using the “log(inhibitor) vs. Normalized response—Variable slope” function in GraphPad Prism 10.1 software.

## 4. Conclusions

In summary, a marine-derived fungal-bacterial symbiont *A*. *spelaeus* GXIMD 04541/*S. echinoides* GXIMD 04532 was obtained from the marine gastropod *Mauritia arabica* collected in shallow coastal waters of southern China. Chemical investigation of this fungal-bacterial symbiont resulted in the isolation of three new cyclic tetrapeptides and two new curvularin analogs, and four known compounds. Their structures were determined by extensive spectroscopic analyses, Marfey’s method, and quantum chemical calculations. Compounds **1**–**5** showed moderate AmB-potentiating activity, and compounds **7** and **8** exhibited moderate antituberculosis activity, with MIC values of 32 and 16 μg/mL, respectively. In addition, compounds **7** and **8** exhibited moderate cytotoxicity against DLD-1, SW480, and B16F10 cell lines with IC_50_ values ranging from 25 to 36 μM. Genomic analysis of *A. spelaeus* identified the biosynthetic gene clusters responsible for producing these metabolites, indicating that the fungal host was the predominant producer. These metabolites may serve as chemical defenses against predators or competitors, thereby playing a critical role in maintaining the stability and survival of the symbiotic system in the marine environment.

## Figures and Tables

**Table 1:** ^1^H NMR (500 MHz) and ^13^C NMR (125 MHz) data for compounds **1**–**3** (in Methanol-*d*_4_, *δ* ppm).

Moiety	No.	1		2		3	
		*δ*_H_ (*J* in Hz)	*δ*_C_, Type	*δ*_H_ (*J* in Hz)	*δ*_C_, Type	*δ*_H_ (*J* in Hz)	*δ*_C_, Type
anthranilic acid	1		172.1, C		172.1, C		172.1, C
	2		125.4, C		125.4, C		125.2, C
	3	7.56, dd (7.6, 1.5)	127.7, CH	7.57, dd (7.6, 1.5)	127.9, CH	7.56, dd (7.6, 1.5)	127.8, CH
	4	7.16, t (7.6)	124.3, CH	7.17, t (7.6)	124.4, CH	7.16, t (7.6)	124.3, CH
	5	7.48, td (7.9, 1.6)	132.6, CH	7.49, td (7.9, 1.6)	132.6, CH	7.49, t (7.9)	132.7, CH
	6	8.34, d (8.2)	122.1, CH	8.32, d (8.2)	122.3, CH	8.34, d (8.2)	122.1, CH
	7		138.1, C		138.1, C		138.2, C
*N*-Me-3-OH-Tyr/ *N*-Me-Tyr	8		170.2, C		170.1, C		170.3, C
	9	4.11, dd (7.3, 3.8)	70.3, CH	4.12, dd (11.1, 4.2)	69.9, CH	4.10, m	70.0, CH
	10	3.28, d (4.0); 3.08, dd (14.2, 11.0)	33.9, CH_2_	3.28, d (4.2); 3.17, dd (14.2, 11.0)	33.5, CH_2_	3.27, dd (14.2, 5.0); 3.20, dd (14.2, 10.9)	33.4, CH_2_
	11		131.0, C		130.2, C		130.1, C
	12	6.69, d (2.2)	117.5, CH	7.06, d (8.1)	131.4, CH	7.01, d (8.1)	131.4, CH
	13		146.6, C	6.74, d (8.4)	116.4, CH	6.74, d (8.4)	116.4, CH
	14		145.2, C		157.4, C		157.4, C
	15	6.70, d (8.1)	116.4, CH	6.74, d (8.4)	116.4, CH	6.74, d (8.4)	116.4, CH
	16	6.54, dd (8.0, 2.1)	121.7, CH	7.06, d (8.1)	131.4, CH	7.01, d (8.1)	131.4, CH
	17	2.95, s	40.7, CH_3_	2.91, s	40.6, CH_3_	2.88, s	40.5, CH_3_
Val/Thr/	18		173.0, C		172.7, C		173.1, C
	19	4.49, d (10.4)	56.2, CH	4.73, d (6.5)	54.9, CH	4.96, t (7.3)	48.0, CH
	20	2.03, m	31.2, CH	3.94, m	68.4, CH	1.61, dd (13.0, 6.3); 1.41, m	41.7, CH_2_
	21	0.94, d (6.6)	20.0, CH_3_	1.16, d (6.2)	20.1, CH_3_	1.56, m	26.0, CH
	22	0.89, d (6.7)	18.7, CH_3_			0.96, d (6.3)	23.3, CH_3_
Ala	23		176.1, C		176.5, C	0.91, d (7.1)	22.8, CH_3_
	24	4.13, q (7.4)	54.9, CH	4.16, q (7.4)	55.0, CH		175.8, C
	25	1.46, d (7.4)	16.1, CH_3_	1.48, d (7.4)	16.0, CH_3_	4.12, m	54.9, CH
	26					1.43, d (7.5)	16.0, CH_3_

**Table 2:** ^1^H NMR (500 MHz) and ^13^C NMR (125 MHz) data for compounds **6** and **7**.

No.		6 ^a^	No.	7 ^b^	
	*δ*_H_ (*J* in Hz)	*δ*_C_, type		*δ*_H_ (*J* in Hz)	*δ*_C_, type
1		172.7, C	1		170.2, C
2	3.65, m	41.0, CH_2_	2	3.30, overlapped; 3.39, overlapped	39.7, CH_2_
3		124.3 ^c^, C	3		133.8, C
4	6.24, d (2.3)	113.0, CH	4	6.19, d (2.2)	109.6, CH
5		163.0, C	5		159.3, C
6	6.26, d (2.3)	103.1, CH	6	6.22, d (2.2)	101.5, CH
7		160.9 ^c^, C	7		157.3, C
8		119.1, C	8		118.0, C
9		203.8, C	9		197.8, C
10	NS	51.5 ^c^, CH_2_	10	6.30, s	133.8, CH
11	5.08, dd (9.5, 4.8)	52.5, CH	11	6.27, dd (8.4, 6.0)	149.0, CH
12	2.01, ddt (14.4, 9.8, 5.4); 1.88, dp (15.0, 5.1)	33.3, CH_2_	12	2.51, overlapped; 2.27, ddd (12.7, 10.5, 8.3)	43.0, CH_2_
13	1.43, m; 1.15, m	23.5, CH_2_	13	3.55, d (8.9)	70.1, CH
14	1.78, ddt (15.1, 10.5, 5.9); 1.45, m	32.4, CH_2_	14	1.81 m; 1.74, d (15.1)	45.7, CH
15	4.87, overlapped	74.4, CH	15	4.83, ddd (12.0, 6.7, 3.4)	70.7, CH
16	1.16, d (6.2)	21.5, CH_3_	16	1.11 d (6.3)	21.3, CH_3_
2′	8.20, s	141.0, CH	5-OH	9.72, s	
4′		125.0, C	7-OH	10.19, s	
5′		150.1, C	13-OH	4.94, s	
7′	8.03, s	146.4, CH			
9′		159.3, C			
NH	4.68, s				

^a^ in Methanol-*d*_4_, *δ* ppm; ^b^ in DMSO-*d*_6_, *δ* ppm, ^c^ showed in HMBC, NS, no showed.

**Table 3:** MIC values of AmB against *C. albicans* in combination with compounds **1**–**5**.

Combination with Compound			MIC (µg/mL)	Ratio
AmB	none		1.0	1
AmB	**1**	8 µg/mL	1.0	1
	**1**	16 µg/mL	0.50	2
	**1**	32 µg/mL	0.25	4
AmB	**2**	8 µg/mL	1.0	1
	**2**	16 µg/mL	1.0	1
	**2**	32 µg/mL	0.50	2
AmB	**3**	8 µg/mL	0.50	2
	**3**	16 µg/mL	0.25	4
	**3**	32 µg/mL	0.125	8
AmB	**4**	8 µg/mL	1.0	1
	**4**	16 µg/mL	0.50	2
	**4**	32 µg/mL	0.25	4
AmB	**5**	8 µg/mL	0.50	2
	**5**	16 µg/mL	0.25	4
	**5**	32 µg/mL	0.125	8

AmB: amphotericin B.

## Data Availability

The data presented in this study are available upon request from the corresponding author.

## Supplementary Materials

The following supporting information can be downloaded at: https://www.mdpi.com/article/10.3390/md24030111/s1, Tables S1–S8: The 1H and 13C NMR data of **1**–**8**; Figure S1–S58: The NMR, HRESIMS spectra of compound **1**–**9**; Figure S59: HPLC analysis spectra of compound **5**, **8**, **9**; Figure S60: UV spectra of compound **1**–**4**, **6**, **7**; Figures S61–S64: Marfey’s analysis spectra of compound **1**–**5**; Tables S9–S11: Relative thermal energies (ΔE), relative free energies (ΔG), and equilibrium populations (P) of low-energy conformers of **6**–**8** in MeOH; Figures S65, S68, S70: Conformations of low-energy conformers of 6–8, respectively; Figures S66, S69, S72: DP4+ analysis result of compounds **6**–**8**, respectively; Figures S67, S73: DP4+ results and linear correlation plots between the experimental and calculated 13C NMR chemical shifts of compound **6**–**8**, respectively. Figure S71: Comparison of the experimental and calculated ECD spectra of **8**; Figure S74: The antituberculosis activity of compounds **7**, **8**; Figure S75: Cytotoxic activity of compounds **1**–**5**, **7**–**9**; Table S12: Line correlation coefficients R2 and mean absolute error (MAE) analyses of the experimental and calculated 13C NMR data of model compounds **6**–**8**; Table S13: The acetylcholinesterase and α-glucosidase activities of compounds **1**–**5**, **7**–**9**; Figure S76: Organization of three NPRS biosynthetic gene clusters; Figure S77: The proposed NRPS biosynthetic pathway for **5**; Figure S78: Structure and arrangement of genes involved in curvularin biosynthesis in the *A. spelaeus* genome; Table S14: Genome features of *A. spelaeus* GXIMD 04541; Table S15: Secondary metabolite biosynthesis gene clusters in *A. spelaeus* GXIMD 04541.
