# Supplementary material for: New Cyclopeptides and Curvularins from Marine-Derived Fungal-Bacterial Symbiont Aspergillus spelaeus GXIMD 04541/Sphingomonas echinoides GXIMD 04532
Source: Mar Drugs. 2026 Mar 15;24(3):111. doi: 10.3390/md24030111 (PMC13027483; doi:10.3390/md24030111)
Supplement: Supplementary file 1 [file marinedrugs-24-00111-s001.zip › marinedrugs-4149630-supplementary.pdf]

New Cyclopeptides and Curvularins from Marine-derived Fungal-bacterial Symbiont *Aspergillus spelaeus* GXIMD 04541/*Sphingomonas echinoides* GXIMD 04532

Fei-Hua Yao [1,2,†], Jie Yang [1,†], Xiao-Yan Li [1], Shu-Fen Xu [1], Kai Liu [1,2], Zhen-Zhou Tang [1,2], Wei-Hui Li [3], Yong-Hong Liu [1,2], Xiang-Xi Yi [1,2,*] and Cheng-Hai Gao [1,2,*]

1 Faculty of Pharmacy/Institute of Marine Drugs, Guangxi University of Chinese Medicine, Nanning 530200, China; hyywyfh@163.com (F.-H.Y.); jieyang202312@163.com (J.Y.); 13457524492@163.com (X.-Y.L.); 18370712882@163.com (S.-F.X.); kailiu@outlook.com (K.L.); trcstrive2015@126.com (Z.-Z.T.); yonghongliu@scsio.ac.cn (Y.-H.L.)

2 Guangxi Key Laboratory of Marine Drugs, University Engineering Research Center of High-efficient Utilization of Marine Traditional Chinese Medicine Resources, Guangxi, Guangxi University of Chinese Medicine, Nanning 530200, China

3 State Key Laboratory for Conservation and Utilization of Subtropical Agro-Bioresources, College of Life Science and Technology, Guangxi University, Nanning 530004, China; lwhlbx@163.com

* Correspondence: yixiangxi2017@163.com (X.-X.Y.); gaoch@gxtcmu.edu.cn (C.-H.G.)

† These authors contributed equally to this work.

Contents

1. Supplementary materials and methods 1

1.1 General experimental procedures 1

1.2 *α*-Glucosidase inhibitory activity 1

1.3 Assessment of acetylcholinesterase (AChE) inhibitory effects 1

1.4 Spectroscopic data of known compounds 4-5, 8-9 2

Table S1. ^{1}H NMR (500 MHz) and ^{13}C NMR (125 MHz) data for compound 1 (in Methanol-d_4, δ ppm) 3

Table S2. ^{1}H NMR (500 MHz) and ^{13}C NMR (125 MHz) data for compound 2 (in Methanol-d_4, δ ppm) 3

Table S3. ^{1}H NMR (500 MHz) and ^{13}C NMR (125 MHz) data for compound 3 (in Methanol-d_4, δ ppm) 4

Table S4. Comparison of ^{1}H and ^{13}C NMR data for compound 4 and *Cyclo*(Ala-*N*MeTyr-Ant-Ala) 4

Table S5. Comparison of ^{1}H and ^{13}C NMR data for compound 5 and Nectriatidel 5

Table S6. ^{1}H NMR (500 MHz) and ^{13}C NMR (125 MHz) data for compound 6 (in Methanol-d_4, δ ppm) 6

Table S7. ^{1}H NMR (500 MHz) and ^{13}C NMR (125 MHz) data for compound 7 (in DMSO-d_6, δ ppm) 6

Table S8. ^{1}H NMR (500 MHz) and ^{13}C NMR (125 MHz) data for compound 8 (in DMSO-d_6, δ ppm) 7

Figure S1 ^{1}H NMR spectrum of 1 in methanol-d_4 8

Figure S2 ^{13}C NMR spectrum of 1 in methanol-d_4 8

Figure S3 DEPT135 spectrum of 1 in methanol-d_4 9

Figure S4 ^{1}H-^{1}H COSY spectrum of 1 in methanol-d_4 9

Figure S5 HSQC spectrum of 1 in methanol-d_4 10

Figure S6 HMBC spectrum of 1 in methanol-d_4 10

Figure S7 NOESY spectrum of 1 in methanol-d_4 11

Figure S8 HR-ESIMS spectrum of 1 11

Figure S9 ^{1}H NMR spectrum of 2 in methanol-d_4 12

Figure S10 ^{13}C NMR spectrum of 2 in methanol-d_4 12

Figure S11 DEPT135 spectrum of 2 in methanol-d_4 13

Figure S12 ^{1}H-^{1}H COSY spectrum of 2 in methanol-d_4 13

Figure S13 HSQC spectrum of 2 in methanol-d_4 14

Figure S14 HMBC spectrum of 2 in methanol-d_4 14

Figure S15 NOESY spectrum of 2 in methanol-d_4 15

Figure S16 HR-ESIMS spectrum of 2 15

Figure S17 ^{1}H NMR spectrum of 3 in methanol-d_4 16

Figure S18 ^{13}C NMR spectrum of 3 in methanol-d_4 16

Figure S19 DEPT135 spectrum of 3 in methanol-d_4 17

Figure S20 ^{1}H-^{1}H COSY spectrum of 3 in methanol-d_4 17

Figure S21 HSQC spectrum of 3 in methanol-d_4 18

Figure S22 HMBC spectrum of 3 in methanol-d_4 18
Figure S23 NOESY spectrum of 3 in methanol-d_4 19
Figure S24 HR-ESIMS spectrum of 3 19
Figure S25 ^{1}H NMR spectrum of 6 in methanol-d_4 20
Figure S26 ^{13}C NMR spectrum of 6 in methanol-d_4 20
Figure S27 DEPT135 spectrum of 6 in methanol-d_4 21
Figure S28 HSQC spectrum of 6 in methanol-d_4 21
Figure S29 HMBC spectrum of 6 in methanol-d_4 22
Figure S30 HR-ESIMS spectrum of 6 22
Figure S31 ^{1}H NMR spectrum of 7 in methanol-d_4 23
Figure S32 ^{13}C NMR spectrum of 7 in methanol-d_4 23
Figure S33 DEPT135 spectrum of 7 in methanol-d_4 24
Figure S34 ^{1}H-^{1}H COSY spectrum of 7 in methanol-d_4 24
Figure S35 HSQC spectrum of 7 in methanol-d_4 25
Figure S36 HMBC spectrum of 7 in methanol-d_4 25
Figure S37 NOESY spectrum of 7 in methanol-d_4 26
Figure S38 HR-ESIMS spectrum of 7 26
Figure S39 ^{1}H NMR spectra of 7 and (+)-(10*E*,15*R*)-13-Hydroxy-10,11-dehydrocurvularin in acetone-d_6 27
Figure S40 Comparison the ^{1}H NMR spectra of 8 and (+)-(10*E*,15*R*)-12-Hydroxy-10,11-dehydrocurvularin in acetone-d_6 27
Figure S41 ^{1}H NMR spectrum of 4 in methanol-d_4 28
Figure S42 ^{13}C NMR spectrum of 4 in methanol-d_4 28
Figure S43 DEPT135 spectrum of 4 in methanol-d_4 29
Figure S44 ^{1}H-^{1}H COSY spectrum of 4 in methanol-d_4 29
Figure S45 HSQC spectrum of 4 in methanol-d_4 30
Figure S46 HMBC spectrum of 4 in methanol-d_4 30
Figure S47 NOESY spectrum of 4 in methanol-d_4 31
Figure S48 HR-ESIMS spectrum of 4 31
Figure S49 ^{1}H NMR spectrum of 5 in methanol-d_4 32
Figure S50 ^{13}C NMR spectrum of 5 in methanol-d_4 32
Figure S51 HR-ESIMS spectrum of 5 33
Figure S52 ^{1}H NMR spectrum of 8 in DMSO-d_6 33
Figure S53 ^{13}C NMR spectrum of 8 in DMSO-d_6 34
Figure S54 HMBC spectrum of 8 in DMSO-d_6 34
Figure S55 HR-ESIMS spectrum of 8 35
Figure S56 ^{1}H NMR spectrum of 9 in DMSO-d_6 35
Figure S57 ^{13}C NMR spectrum of 9 in DMSO-d_6 36
Figure S58 HR-ESIMS spectrum of 9 36
Figure S59 HPLC analysis spectra of compounds 5, 8-9 36
Figure S60 UV spectra of compounds 1-4, 6-7 37
Figure S61 HPLC analysis of FDAA derivates of standard amino acids and compound 1 38

Figure S62 HPLC analysis of FDAA derivates of standard amino acids and compound 2..39
Figure S63 HPLC analysis of FDAA derivates of standard amino acids and compound 3..40
Figure S64 HPLC analysis of FDAA derivates of standard amino acids and compounds 4-5..41
Figure S65 Conformations of low-energy conformers of 6 in MeOH...........42
Table S9 Relative thermal energies (ΔE), relative free energies (ΔG), and equilibrium populations (P) of low-energy conformers of 6 in MeOH.........43
Figure S66 DP4+ analysis result of 6 (experimental for 6, isomer 1 for (11*S*,15*S*)-6a, isomer 2 for (11*S*,15*S*)-6b) 43
Figure S67 DP4+ results and linear correlation plots between the experimental and calculated ^{13}C NMR chemical shifts of compound 6........44
Figure S68 Conformations of low-energy conformers of 7 in MeOH...........44
Table S10 Relative thermal energies (ΔE), relative free energies (ΔG), and equilibrium populations (P) of low-energy conformers of 7 in MeOH.........45
Figure S69 DP4+ analysis result of 7 (experimental for 7, isomer 1 for (13*R*,15*S*)-7, isomer 2 for (13*S*,15*S*)-7) 46
Figure S70 Conformations of low-energy conformers of 8 in MeOH...........47
Figure S71 Comparison of the experimental and calculated ECD spectra of 8 47
Table S11 Relative thermal energies (ΔE), relative free energies (ΔG), and equilibrium populations (P) of low-energy conformers of 8 in MeOH.........48
Figure S72 DP4+ analysis result of 8 (experimental for 8, isomer 1 for (12*R*,15*S*)-8, isomer 2 for (12*S*,15*S*)-8) 48
Figure S73 DP4+ results and linear correlation plots between the experimental and calculated ^{13}C NMR chemical shifts of compounds 7-8...49
Table S12 Line correlation coefficients R^2 and mean absolute error (MAE) analyses of the experimental and calculated ^{13}C NMR data of model compounds 6-8...49
Figure S74 The anti-*Mycobacterium tuberculosis* activity of compounds 7-8 50
Figure S75 The cytotoxicity of compounds 1-5, 7-9 against cell lines SW480, B16F10, DLD-1, PC-3 and 22Rv1...50
Table S13 The acetylcholinesterase and *α*-glucosidase activities of compounds 1-5, 7-9..51
Figure S76 Organization of three NPRS biosynthetic gene clusters.............51
Figure S77 The proposed NRPS biosynthetic pathway for 5........................52
Figure S78 Structure and arrangement of genes involved in curvularin biosynthesis in the *Aspergillus spelaeus* genome. 52
Table S14 Genome features of *Aspergillus spelaeus* GXIMD 04541 53
Table S15 Secondary metabolite biosynthesis gene clusters in *Aspergillus spelaeus* GXIMD 04541 54

1. Supplementary materials and methods

1.1 General experimental procedures

α-glucosidase was purchased from Sigma-Aldrich (Shanghai) Trading Co., Ltd., and 4-nitrophenyl-*α*-*D*-glucoside was purchased from Shanghai Aladdin Biochemical Technology Co., Ltd. Dimethyl sulfoxide (DMSO) was obtained from Sigma-Aldrich (St. Louis, MO, USA); Sodium carbonate and disodium hydrogen phosphate were provided by the Tianjin Damao Chemical Reagent Factory. Sodium dihydrogen phosphate and acetylcholinesterase were from Beijing Taiyang Biotechnology Co., Ltd. acetylthiocholine iodide and 5,5'-dithiobis(2-nitrobenezoic acid) were purchased from Tianjin Saitong Biotechnology Co., LTD provides. Tacrine hydrochloride hydrate was provided by Shanghai Maclean Biochemical Technology Co. Ltd. Acarbose and dimolybdenum tetraacetate are obtained from Shanghai Titan Technology Co. Ltd.

1.2 *α*-Glucosidase inhibitory activity

The inhibitory activity of compounds **1**-**5**, **7**-**9** against *α*-glucosidase were investigated. Briefly, *α*-Glucosidase and 4-nitrophenyl-*α*-*D*-glucopyranoside (PNPG) were prepared in 0.1 M potassium phosphate buffer (pH 6.8), and compounds **1**-**9** were dissolved in 10% DMSO. Then, 20 μL of *α*-glucosidase (final concentration 1 U/mL), 60 μL of phosphate buffer and 20 μL of the test compounds (final concentration 0.25mg/mL) were added into 96-well plates. After incubated at 37 °C for 15 min, 20 μL of PNPG (concentration 2.5 mM) was added, followed by incubation at 37 °C for 30 min. The reaction was terminated by adding 80 μL of 0.2 M Na_2CO_3 solution. Prepare blank controls, negative controls, positive controls (acarbose solution with a final concentration of 0.25 mg/ml), test groups, and blank groups. The absorbance at 405 nm was measured, and the *α*-glucosidase inhibitory rate for each compound was calculated accordingly.

1.3 Assessment of acetylcholinesterase (AChE) inhibitory effects

The AChE inhibitory activity of compounds **1**-**5**, **7**-**9** against *α*-glucosidase were investigated. Briefly, 0.1 M phosphate buffer solution (PBS, pH 8.0) was prepared. The substrate and enzyme solutions were prepared at 6.25 mM

thioacetylcholine and 0.1 U/mL AChE, respectively. Test samples and the positive control (tacrine) were dissolved in 2% DMSO (final concentrations of 0.05 mg/mL and 333 nM, respectively). The reaction mixture was assembled in a 96-well plate, comprising blank, negative control, positive control, test sample, and background control wells. PBS, 2% DMSO, tacrine, test sample, and AChE solution were added sequentially to a total volume of 200 μL. After incubation at 30 °C for 20 min, 40 μL of a freshly mixed solution of DTNB and thioacetylcholine (both at a final concentration of 0.625 mM) was added to each well. The background absorbance was immediately measured at 405 nm, and the final absorbance was recorded after an additional 30 min incubation at 30 °C.

1.4 Spectroscopic data of known compounds 4-5, 8-9

Cyclo(Ala-*N*MeTyr-Ant-Ala) (**4**): white powder; $[\alpha]_D^{26}$ – 72.08 (*c* 0.05, MeOH); UV (MeOH) λ_{max} (log ε) 220 (4.25), 252 (3.88), 286 (3.38) nm; HR-ESIMS: *m/z* 461.1801 $[M+Na]^+$ (calcd for $C_{23}H_{26}NaN_4O_5^+$, 461.1801); ^{1}H NMR (500 MHz) and ^{13}C NMR (125 MHz, Methanol-d_4) in **Table S4**.

Nectriatidel (**5**): white amorphous powder; $[\alpha]_D^{26}$ –116.09 (*c* 0.05, MeOH); HR-ESIMS *m/z* 489.2100 $[M+Na]^+$(calcd. for $C_{25}H_{30}N_4O_5Na^+$ 489.2114). ^{1}H NMR (500 MHz) and ^{13}C NMR (125 MHz, Methanol-d_4) in **Table S5**.

ent-Curvulone A (**9**): white amorphous powder; $[\alpha]_D^{26}$ 42.05 (*c* 0.05, MeOH); HR-ESIMS *m/z*: 305.1030 $[M+H]^+$ (calcd for $C_{16}H_{17}O_6^+$ 305.1025). ^{1}H NMR (500 MHz, DMSO-d_6) δ_H 6.32 (1H, d, *J* = 1.8 Hz, H-4), 6.28 (1H, d, *J* = 1.8 Hz, H-6), 5.01 (1H, t, *J* = 4.3 Hz, H-10), 4.52 – 4.44 (1H, m, H-15), 3.92 (1H, d, *J* = 17.6 Hz, H-2), 3.61 (1H, d, *J* = 17.6 Hz, H-2), 3.02 (1H, dd, *J* = 13.1, 4.5 Hz, H-11a), 2.69 (1H, dd, *J* = 13.1, 4.3 Hz, H-11b), 2.43 (1H, ddd, *J* = 19.7, 10.4, 3.5 Hz, H-13a), 2.37 – 2.29 (1H, m, H-13b), 1.67 (1H, tdd, *J* = 11.5, 5.5, 3.6 Hz, H-14a), 1.40 (1H, ddt, *J* = 14.2, 10.4, 3.2 Hz, H-14b), 1.02 (3H, d, *J* = 6.1 Hz, H-16); ^{13}C NMR (125 MHz, DMSO-d_6) δ_C 204.70 (C-12), 197.3 (C-9), 175.3 (C-7), 168.8 (C-1), 168.5(C-5), 135.8 (C-3), 113.8 (C-4), 111.4 (C-8), 96.6 (C-6), 82.3 (C-10), 72.1 (C-15), 43.2 (C-11), 40.9 (C-13), 38.2 (C-2), 26.2 (C-14), 20.3 (C-16).

Table S1. ^{1}H NMR (500 MHz) and ^{13}C NMR (125 MHz) data for compound 1 (in Methanol-d_4, δ ppm)

moiety	No.	δ_H (J in Hz)	δ_C, type	COSY	HMBC
anthranilic acid	1		172.1, C		
	2		125.4, C		
	3	7.56, dd (7.6, 1.5)	127.7, CH	H-4	C-1(weak), C-5, C-7
	4	7.16, t (7.6)	124.3, CH	H-3, H-5	C-2, C-6, C-7
	5	7.48, td (7.9, 1.6)	132.6, CH	H-4, H-6	C-3, C-7
	6	8.34, d (8.2)	122.1, CH	H-5	C-1, C-2, C-4, C-7
	7		138.1, C		
	8		170.2, C		
N-Me-3-OH-Tyr	9	4.11, dd (7.3, 3.8)	70.3, CH	H-10	C-8, C-10, C-11, C-17, C-18
	10	3.28, d (4.0); 3.08, dd (14.2, 11.0)	33.9, CH_2	H-9	C-8, C-9, C-11, C-12, C-16
	11		131.0, C		
	12	6.69, d (2.2)	117.5, CH		C-10, C-11, C-13, C-14, C-16
	13		146.6, C		
	14		145.2, C		
	15	6.70, d (8.1)	116.4, CH	H-16	C-11, C-13
	16	6.54, dd (8.0, 2.1)	121.7, CH	H-15	C-10, C-12, C-14, C-15
	17	2.95, s	40.7, CH_3		C-9, C-18
Val	18		173.0, C		
	19	4.49, d (10.4)	56.2, CH	H-20	C-18, C-20, C-21, C-23
	20	2.03, m	31.2, CH	H-19, H-21, H-22	C-19, C-21, C-22
	21	0.94, d (6.6)	20.0, CH_3	H-20	C-19, C-20, C-22
	22	0.89, d (6.7)	18.7, CH_3	H-20	C-19, C-20, C-21
Ala	23		176.1, C		
	24	4.13, q (7.4)	54.9, CH	H-25	C-1, C-23, C-25
	25	1.46, d (7.4)	16.1, CH_3	H-24	C-23, C-24

Table S2. ^{1}H NMR (500 MHz) and ^{13}C NMR (125 MHz) data for compound 2 (in Methanol-d_4, δ ppm)

moiety	No.	δ_H (J in Hz)	δ_C, type	COSY	HMBC
anthranilic acid	1		172.1, C		
	2		125.4, C		
	3	7.57, dd (7.6, 1.5)	127.9, CH	H-4	C-1, C-5, C-7
	4	7.17, t (7.6)	124.4, CH	H-3, H-5	C-2, C-6, C-7(weak)
	5	7.49, td (7.9, 1.6)	132.6, CH	H-4, H-6	C-3, C-7
	6	8.32, d (8.2)	122.3, CH	H-5	C-1(weak), C-2, C-4, C-7
	7		138.1, C		
	8		170.1, C		
N-Me-Tyr	9	4.12, dd (11.1, 4.2)	69.9, CH	H-10	C-8, C-10, C-11, C-17, C-18
	10	3.28, d (4.2); 3.17, dd (14.2, 11.0)	33.5, CH_2	H-9	C-8, C-9, C-11, C-12/16
	11		130.2, C		
	12/16	7.06, d (8.1)	131.4, CH	H-13/H-15	C-12/16, C-13/15, C-14
	13/15	6.74, d (8.4)	116.4, CH	H-12/H-16	C-11, C-13/15, C-14
	14		157.4, C		
	17	2.91, s	40.6, CH_3		C-9, C-18
Thr	18		172.7, C		
	19	4.73, d (6.5)	54.9, CH	H-20	C-18, C-20, C-21, C-22
	20	3.94, m	68.4, CH	H-21	C-18, C-19, C-21
	21	1.16, d (6.2)	20.1, CH_3	H-20	C-19, C-20
Ala	22		176.5, C		
	23	4.16, q (7.4)	55.0, CH	H-24	C-1, C-22, C-24
	24	1.48, d (7.4)	16.0, CH_3	H-23	C-22, C-23

Table S3. ^1H NMR (500 MHz) and ^{13}C NMR (125 MHz) data for compound 3 (in Methanol-d_4, δ ppm)

moiety	No.	δ_H (*J* in Hz)	δ_C, type	COSY	HMBC
anthranilic acid	1		172.1, C		
	2		125.2, C		
	3	7.56, dd (7.6, 1.5)	127.8, CH	H-4	C-1, C-5, C-7
	4	7.16, t (7.6)	124.3, CH	H-3, H-5	C-2, C-6
	5	7.49, t (7.9)	132.7, CH	H-4, H-6	C-3, C-7
	6	8.34, d (8.2)	122.1, CH	H-5	C-2, C-4, C-7
	7		138.2, C		
N-Me-Tyr	8		170.3, C		
	9	4.10, m	70.0, CH	H-10	C-8, C-10, C-11, C-17, C-18
	10	3.27, dd (14.2, 5.0); 3.20, dd (14.2, 10.9)	33.4, CH_2	H-9	C-8 (weak), C-9, C-12/16
	11		130.1, C		
	12/16	7.01, d (8.1)	131.4, CH	H-13/H-15	C-10, C-12/16, C-14
	13/15	6.74, d (8.4)	116.4, CH	H-12/H-16	C-14, C-11, C-13/15
	14		157.4, C		
	17	2.88, s	40.5, CH_3		C-9, C18
	18		173.1, C		
Leu	19	4.96, t (7.3)	48.0, CH	H-20	C-18, C-20, C-21, C-24
	20	1.61, dd (13.0, 6.3); 1.41, m	41.7, CH_2	H-19, H-21	C-19, C-21, C-22, C-23
	21	1.56, m	26.0, CH	H-20, H-22, H-23	C-20, C-22, C-23
	22	0.96, d (6.3)	23.3, CH_3	H-21	C-20, C-21, C-23
	23	0.91, d (7.1)	22.8, CH_3	H-21	C-20, C-21, C-22
Ala	24		175.8, C		
	25	4.12, m	54.9, CH	H-26	C-1, C-24, C-26
	26	1.43, d (7.5)	16.0, CH_3	H-25	C-24, C-25

Table S4. Comparison of ^1H and ^{13}C NMR data for compound 4 and *Cyclo*(Ala-*N*MeTyr-Ant-Ala)

moiety	No.	**4** (in Methanol-d_4)		*Cyclo*(Ala-*N*MeTyr-Ant-Ala) (in DMSO-d_6)[1]	
		δ_H (*J* in Hz)	δ_C, type	δ_H (*J* in Hz)	δ_C, type
anthranilic acid	1		172.0, C		169.7
	2		125.3, C		124.3
	3	7.56, dd (7.7, 1.5)	127.9, CH	7.55, dd (8.0, 1.0)	127.0
	4	7.17, t (7.6)	124.3, CH	7.12, dt (8.0, 1.0)	122.4
	5	7.49, t (7.9)	132.7, CH	7.47, dt (8.0, 1.5)	131.2
	6	8.32, d (8.2)	122.2, CH	8.27, dd (8.0, 1.0)	119.9
	7		138.2, C		136.9
N-Me-Tyr	8		170.3, C		168.1
	9	4.06, dd (8.5, 7.2)	69.9, CH	4.07, dd (10.0, 5.0)	67.6
	10	3.24, d (7.9)	33.2, CH_2	3.10, dd (13.5, 5.0); 3.05, dd (13.5, 10.0)	31.7
	11		130.2, C		128.7
	12/16	7.00, d (8.3)	131.4, CH	6.92, d (8.5)	130.0
	13/15	6.75, d (8.3)	116.4, CH	6.67, d (8.5)	115.2
	14		157.4, C		155.7
	17	2.85, s	40.4, CH_3	2.74, s	39.2
Ala	18		173.6, C		171.4
	19	5.02, q (6.6)	45.2, CH	4.85, dq (9.0, 6.5)	42.8
	20	1.23, d (6.6)	17.6, CH	1.10, d (6.5)	17.4
Ala	21		175.7, C		172.0

22	4.11, q (7.4)	54.9, CH	3.92, dq (7.0, 5.0)	53.2
23	1.42, d (7.4)	16.0, CH_3	1.30, d (7.0)	15.5

Reference: [1] Nagai, K.; Kobayashi, K.; Miyake, R.; Sato, Y.; Seki, R.; Fukuda, T.; Yagi, A.; Uchida, R.; Ohshiro, T.; Tomoda, H. Synthesis and biological evaluation of nectriatide derivatives, potentiators of amphotericin B activity. *J Antibiot.* **2024**, 77, 214–220.

Table S5. Comparison of ^{1}H and ^{13}C NMR data for compound 5 and Nectriatidel

moiety	No.	**5** (in Methanol-d_4)		Nectriatidel (in DMSO-d_6)[2]	
		δ_H (*J* in Hz)	δ_C, type	δ_H (*J* in Hz)	δ_C, type
anthranilic acid	1		172.0, C		169.8, C
	2		125.3, C		124.4, C
	3	7.56, dd (7.7, 1.6)	127.8, CH	7.56, dd (8.5, 2.0)	126.8, CH
	4	7.16, dd (7.7, 1.1)	124.3, CH	7.12, td (8.5, 2.0)	122.3, CH
	5	7.48, ddd (8.8, 7.5, 1.6)	132.6, CH	7.47, td (8.5, 2.0)	131.1, CH
	6	8.33, d (8.1)	122.1, CH	8.29, d (8.5)	119.9, CH
	7		138.0, C		136.8, C
	8		170.1, C		167.9, C
	9	4.13, m	70.3, CH	4.17, dd (10.5, 4.0)	67.8, CH
N-Me-Tyr	10	3.32, m; 3.15, dd (14.2, 11.1)	33.6, CH_2	3.17, m 3.00, dd (12.0, 9.0)	32.1, CH_2
	11		130.2, C		128.6, C
	12/16	7.05, d (8.4)	131.4, CH	6.98, d (8.0)	130.0, CH
	13/15	6.73, d (8.4)	116.4, CH	6.65, d (8.0)	115.0, CH
	14		157.3, C		155.7, C
	17	2.91, s	40.6, CH_3	2.79, s	39.5, CH_3
Val	18		173.0, C		170.8, C
	19	4.47, d (10.3)	56.2, CH	4.31, t (11.0)	53.8, CH
	20	2.03, m	31.1, CH	1.95, m	29.3, CH
	21	0.92, d (6.6)	20.0, CH_3	0.81, d (7.0)	19.2, CH_3
	22	0.89, d (6.8)	18.7, CH_3	0.77, d (7.0)	18.1, CH_3
Ala	23		172.4, C		172.4, C
	24	4.13, m	54.9, CH	3.93, m	53.2, CH
	25	1.45, d (7.3)	15.5, CH_3	1.32, d (7.0)	15.5, CH_3

Reference: [2] Fukuda, T.; Nagai, K.; Yagi, A.; Kobayashi, K.; Uchida, R.; Yasuhara, T.; Tomoda, H. Nectriatide, a potentiator of amphotericin B activity from *Nectriaceae* sp. BF-0114. J. Nat. Prod. 2019, 82, 2673–2681.

Table S6. ^{1}H NMR (500 MHz) and ^{13}C NMR (125 MHz) data for compound 6 (in Methanol-d_4, δ ppm)

No.	δ_H (*J* in Hz)	δ_C, type	HMBC
1		172.7, C	
2	3.65, m	41.0, CH_2	
3		124.3 [c], C	
4	6.24, d (2.3)	113.0, CH	C-2, C-3, C-5, C-6, C-8
5		163.0, C	
6	6.26, d (2.3)	103.1, CH	C-4, C-8
7		160.9 [c], C	
8		119.1, C	
9		203.8, C	
10	NS	51.5[c], CH_2	
11	5.08, dd (9.5, 4.8)	52.5, CH	C-9, C-10, C-12, C-2', C-5'
12	2.01, ddt (14.4, 9.8, 5.4); 1.88, dp (15.0, 5.1)	33.3, CH_2	C-11, C-13, C-14
13	1.43, m; 1.15, m	23.5, CH_2	C-12, C-14, C-15
14	1.78, ddt (15.1, 10.5, 5.9); 1.45, m	32.4, CH_2	C-13, C-15
15	4.87, overlapped	74.4, CH	C-1, C-13
16	1.16, d (6.2)	21.5, CH_3	C-14, C-15
2′	8.20, s	141.0, CH	C-4′, C-5′
4′		125.0, C	
5′		150.1, C	
7′	8.03, s	146.4, CH	C-5′, C-9′
9′		159.3, C	
NH	4.68, brs		

[c] showed in HMBC, NS, no showed.

Table S7. ^{1}H NMR (500 MHz) and ^{13}C NMR (125 MHz) data for compound 7 (in DMSO-d_6, δ ppm)

No.	δ_H (*J* in Hz)	δ_C, type	COSY	HMBC
1		170.2, C		
2	3.30, overlapped; 3.39, overlapped	39.7, CH_2		C-1, C-3, C-4, C-8
3		133.8, C		
4	6.19, d (2.2)	109.6, CH		C-2, C-5, C-6, C-8
5		159.3, C		
6	6.22, d (2.2)	101.5, CH		C-4, C-5, C-7, C-8
7		157.3, C		
8		118.0, C		
9		197.8, C		
10	6.30, s	133.8, CH	H-11	C-8, C-9, C-11, C-12
11	6.27, dd (8.4, 6.0)	149.0, CH	H-10, H-12	C-9, C-10, C-12
12	2.51, overlapped; 2.27, ddd (12.7, 10.5, 8.3)	43.0, CH_2	H-11, H-13	C-10, C-11, C-13, C-14
13	3.55, d (8.9)	70.1, CH	H-12, H-14	
14	1.81 m; 1.74, d (15.1)	45.7, CH	H-13, H-15	C-12, C-13
15	4.83, ddd (12.0, 6.7, 3.4)	70.7, CH	H-14, H-16	C-1, C-13
16	1.11 d (6.3)	21.3, CH_3	H-15	C-14, C-15
5-OH	9.72, s			
7-OH	10.19, s			
13-OH	4.94, s			

Table S8. ^1H NMR (500 MHz) and ^{13}C NMR (125 MHz) data for compound 8 (in DMSO-d_6, δ ppm)

**No.**	δ_H (J in Hz)	δ_C, type	HMBC
1		170.2, C	
2	3.26, d (14.6); 3.15, d (14.8)	39.0, CH_2	C-1, C-3, C-4, C-8
3		132.5, C	
4	6.21, d (2.1)	108.6, CH	C-2, C-5, C-6, C-8
5		158.4, C	
6	6.23, d (2.1)	101.4, CH	C-4, C-5, C-7, C-8
7		155.6, C	
8		119.1, C	
9		198.4, C	
10	6.26, s	129.8, CH	C-8, C-9, C-11, C-12
11	6.28, d (4.1)	156.7, CH	C-9, C-10, C-12
12	5.14, d (4.0)	68.2, CH	C-11, C-13
13	1.87, dt (15.9, 7.9); 1.52, m	32.2, CH_2	C-12, C-14, C-15
14	1.70, m; 1.45, dd (15.5, 9.6)	26.9, CH_2	C-12, C-15
15	4.74, dq (7.7, 5.6, 3.8)	72.4, CH	C-1, C-13
16	1.09 d (6.3)	20.5, CH_3	C-14, C-15
5/7-OH	9.68, brs		
12-OH	4.27, dd (7.4, 3.8)		

Figure S1 ^{1}H NMR spectrum of 1 in methanol-d_4

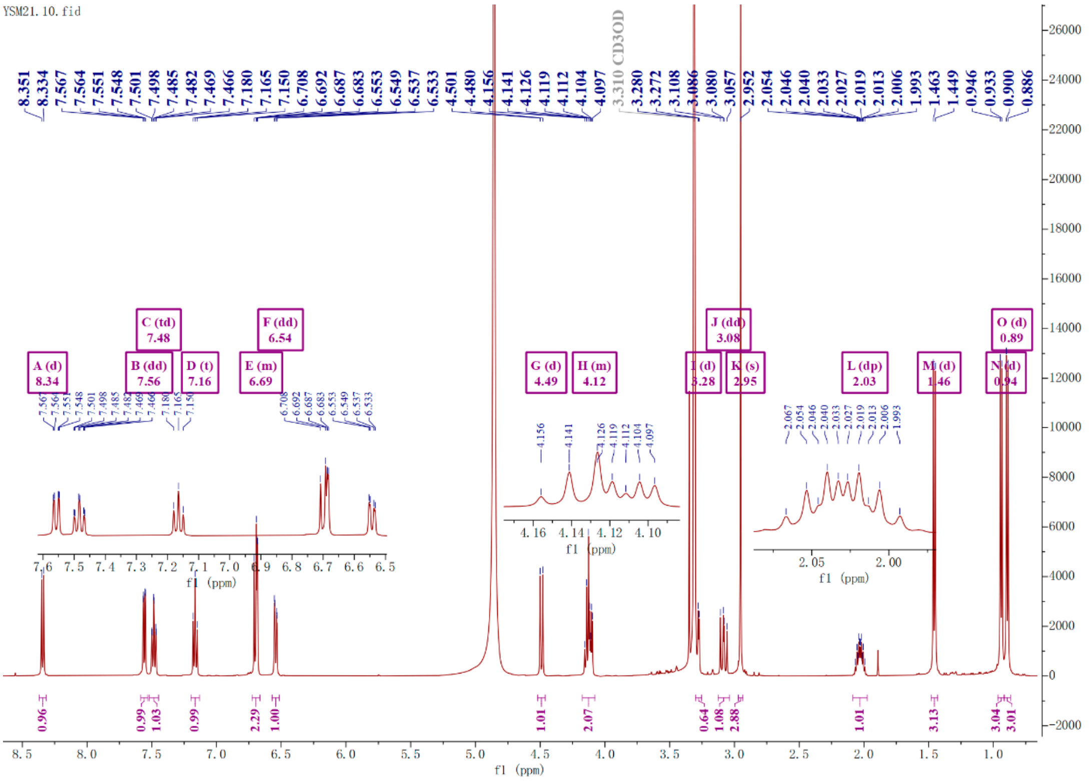

Figure S2 ^{13}C NMR spectrum of 1 in methanol-d_4

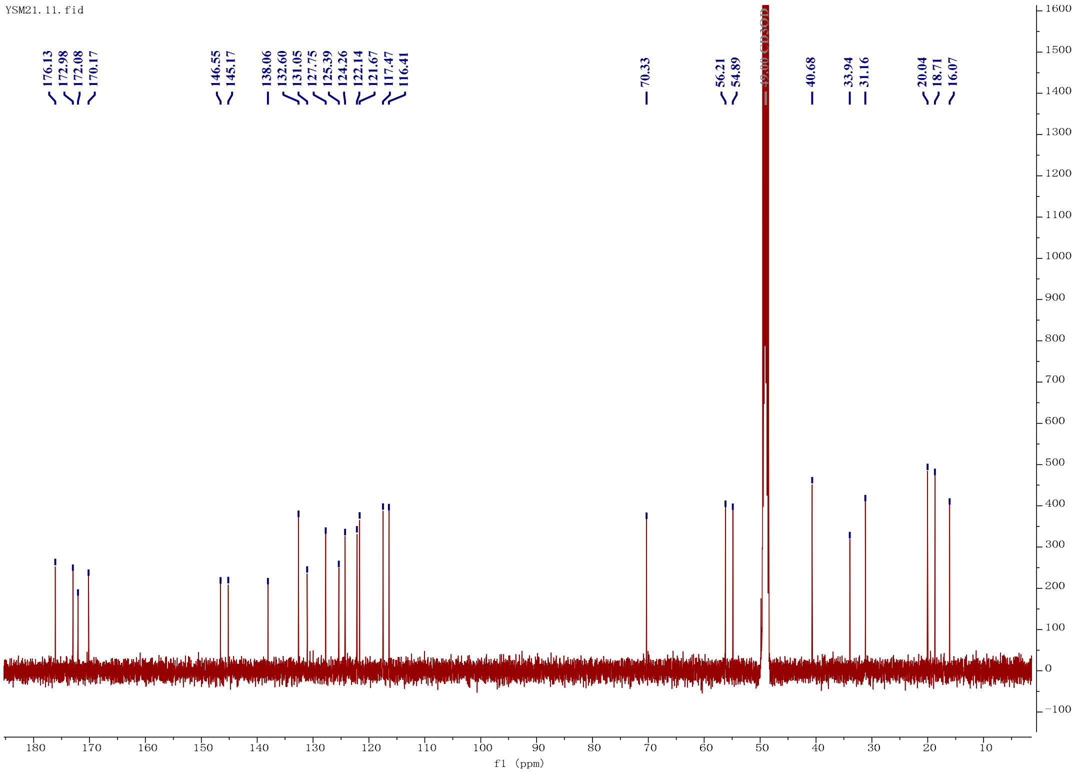

Figure S3 DEPT135 spectrum of 1 in methanol-d_4

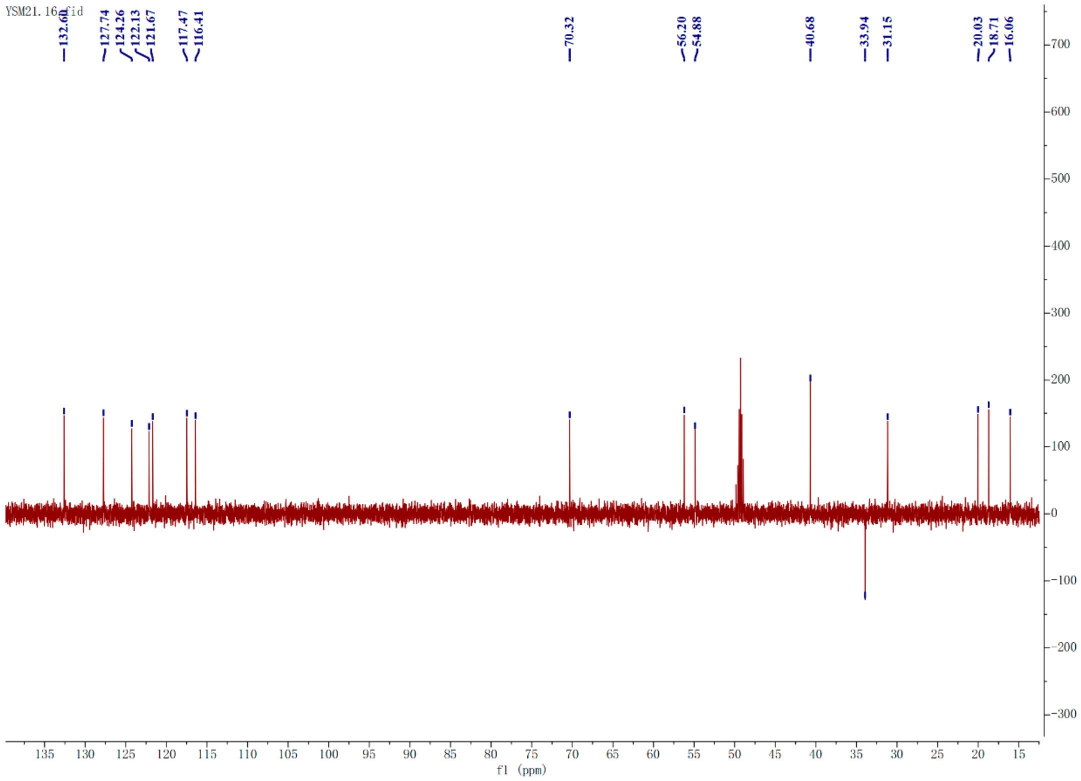

Figure S4 ^1H-^1H COSY spectrum of 1 in methanol-d_4

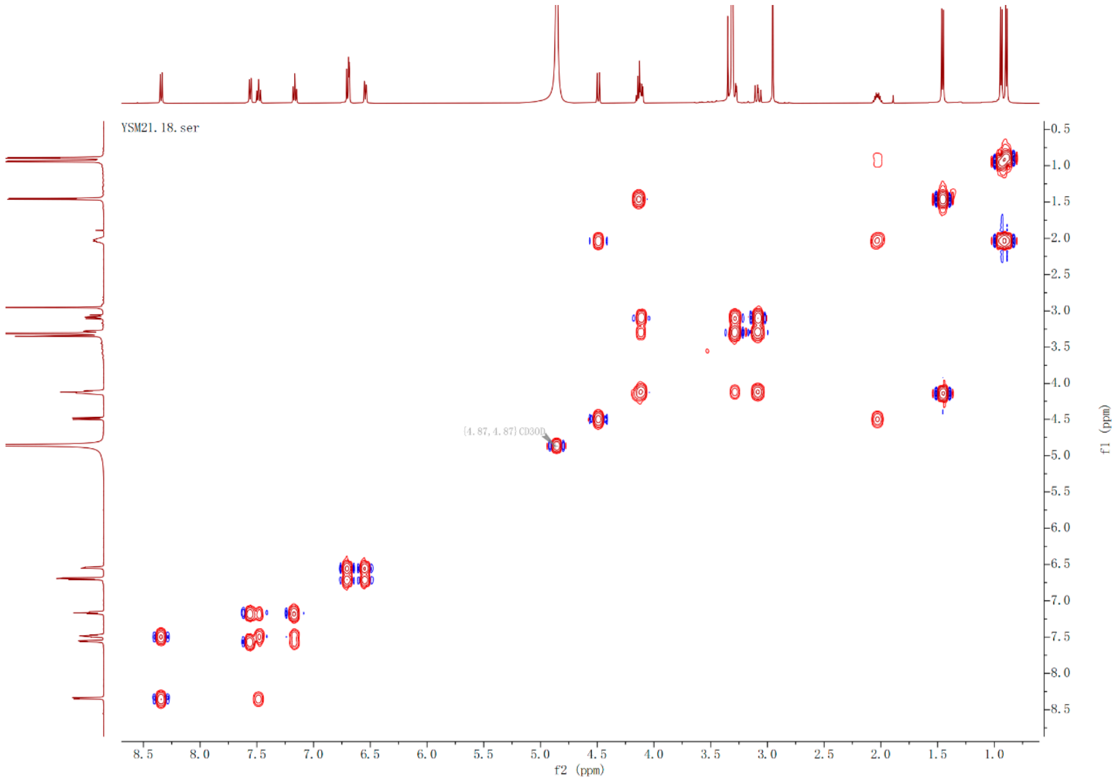

Figure S5 HSQC spectrum of 1 in methanol-d_4

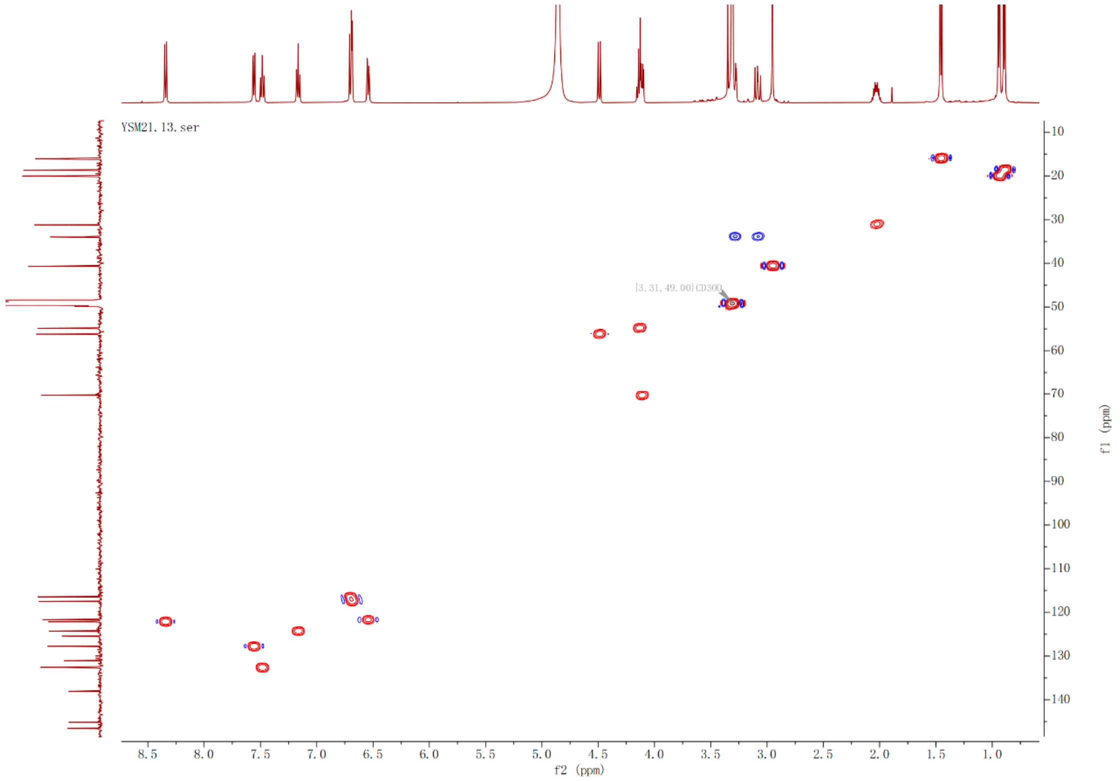

Figure S6 HMBC spectrum of 1 in methanol-d_4

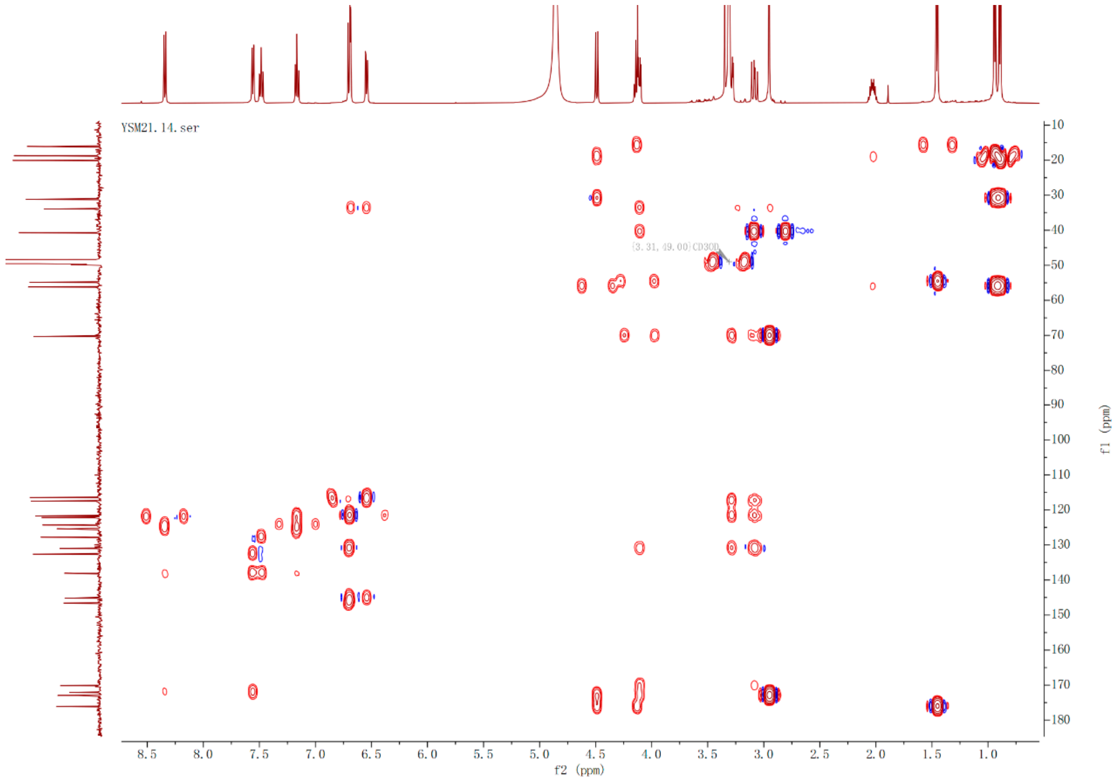

Figure S7 NOESY spectrum of 1 in methanol-d_4

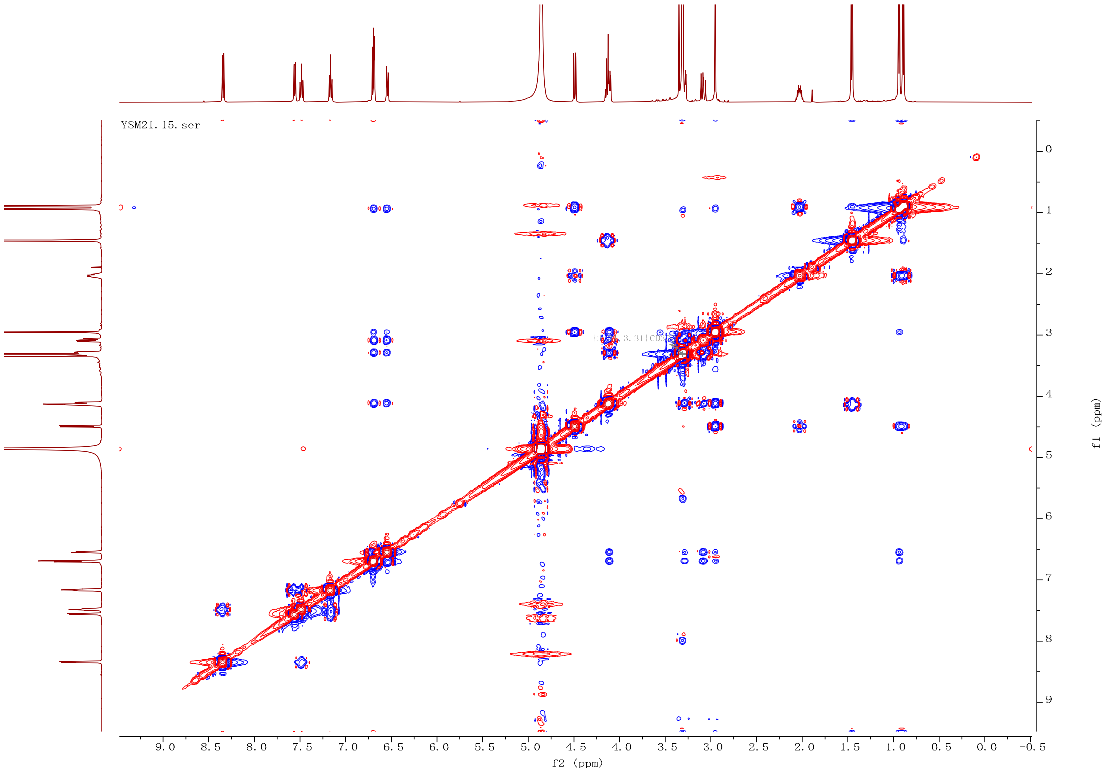

Figure S8 HR-ESIMS spectrum of 1

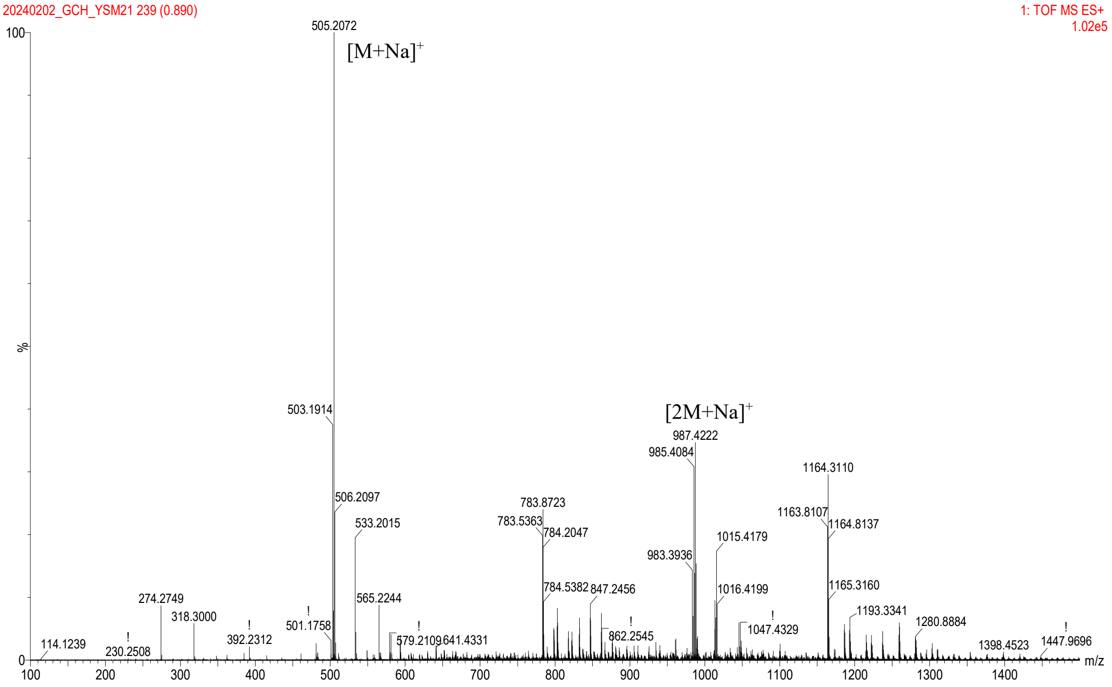

Figure S9 ^{1}H NMR spectrum of 2 in methanol-d_4

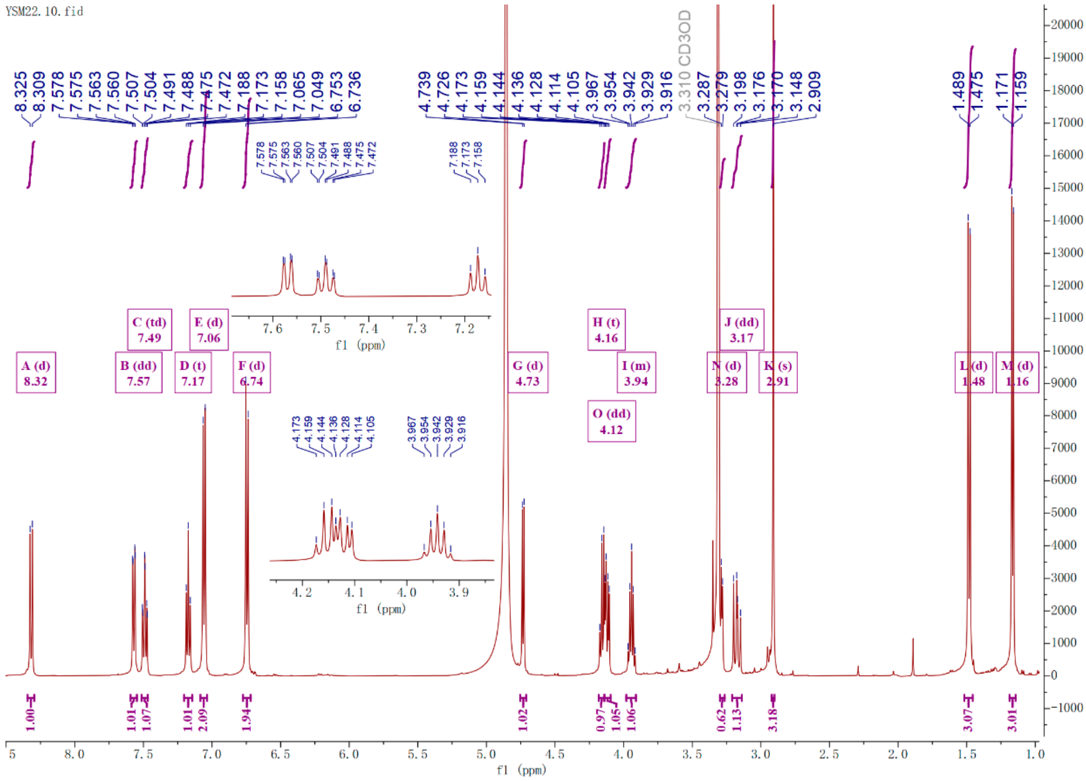

Figure S10 ^{13}C NMR spectrum of 2 in methanol-d_4

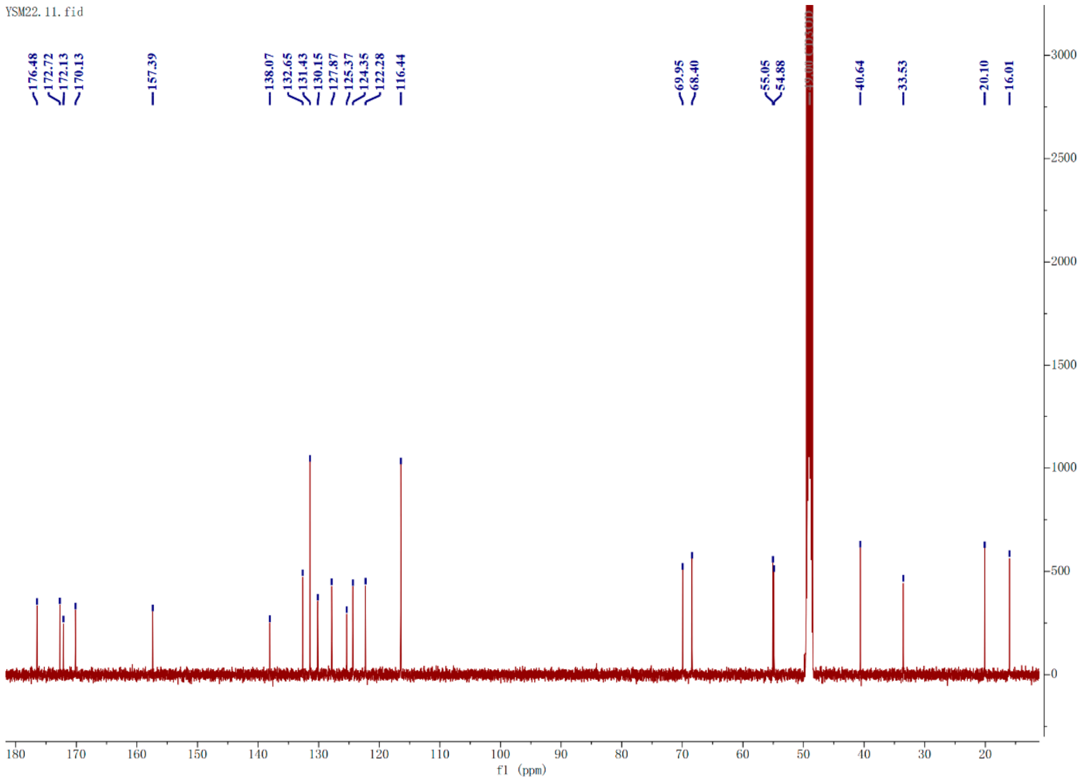

Figure S11 DEPT135 spectrum of 2 in methanol-d_4

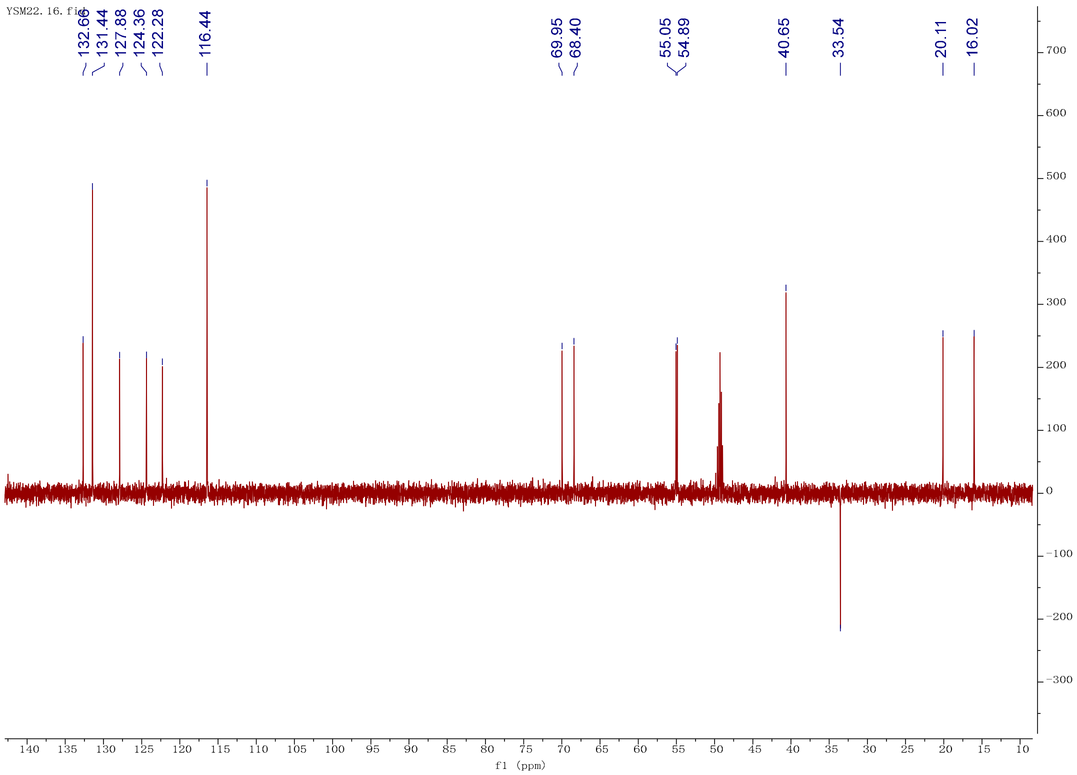

Figure S12 ^{1}H-^{1}H COSY spectrum of 2 in methanol-d_4

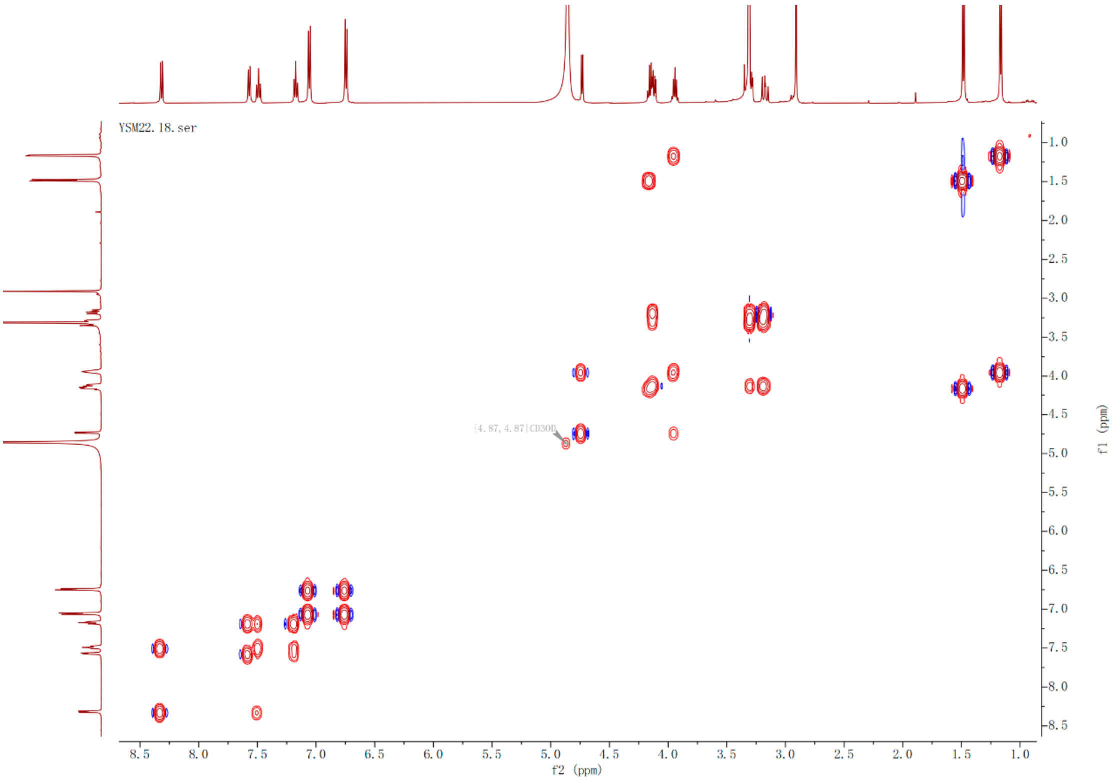

Figure S13 HSQC spectrum of 2 in methanol-d_4

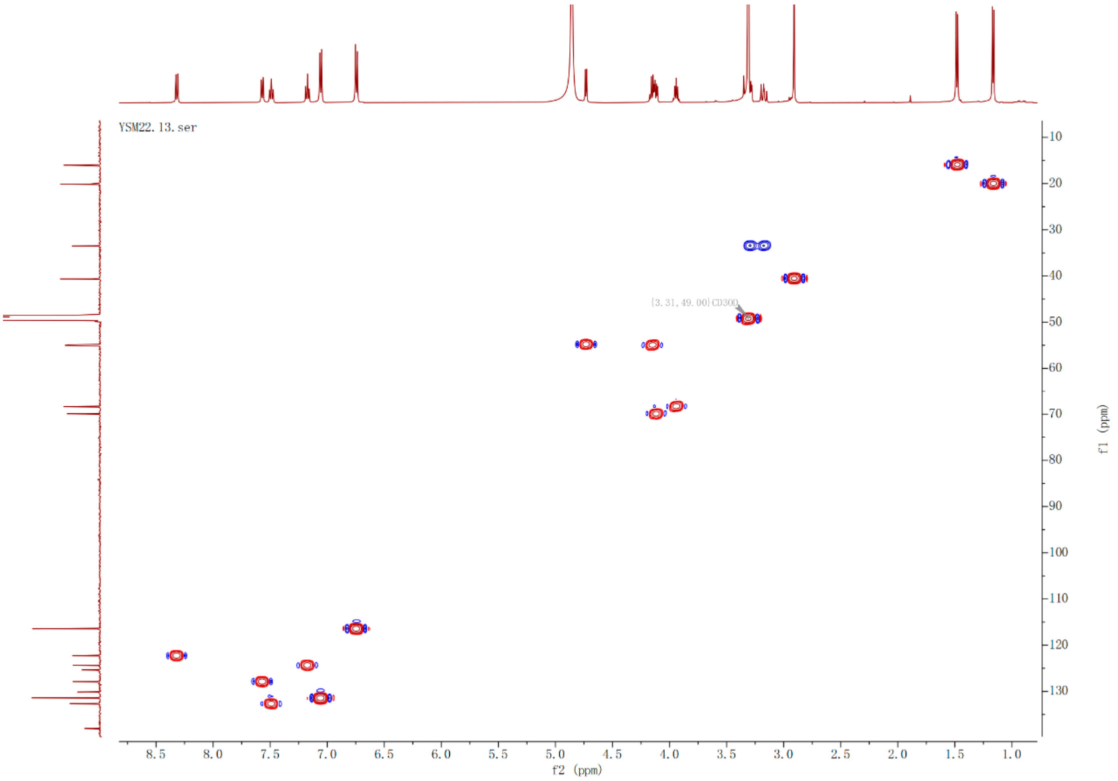

Figure S14 HMBC spectrum of 2 in methanol-d_4

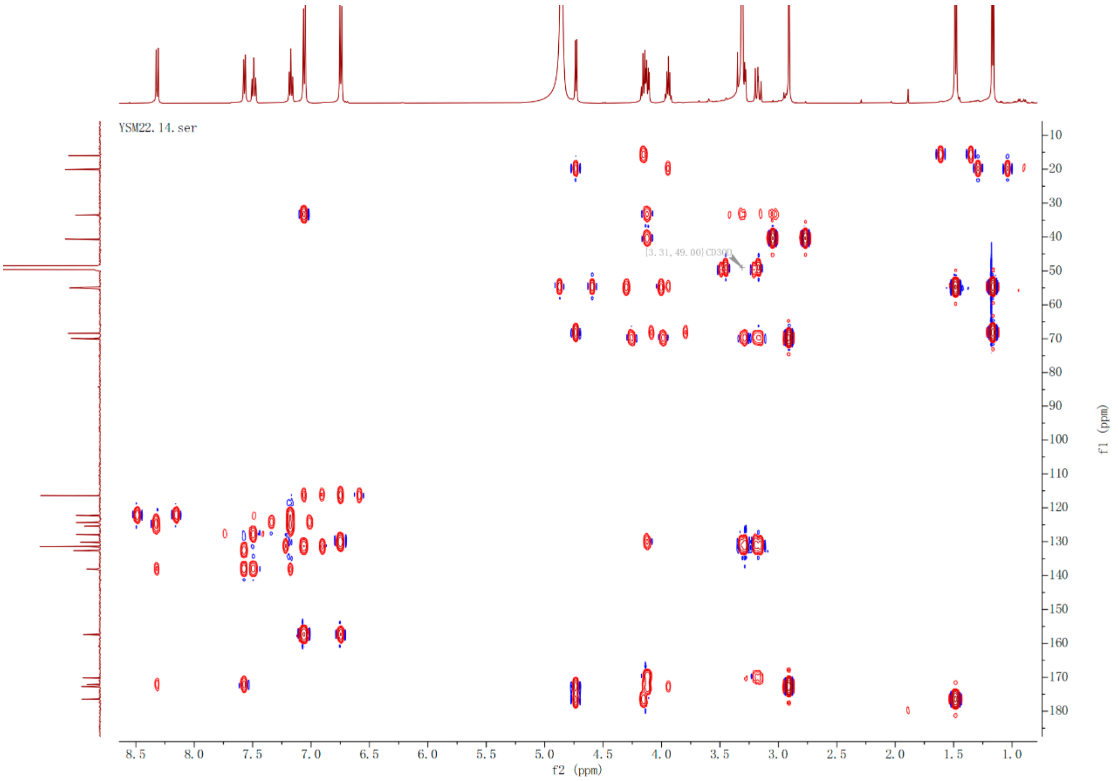

Figure S15 NOESY spectrum of 2 in methanol-d_4

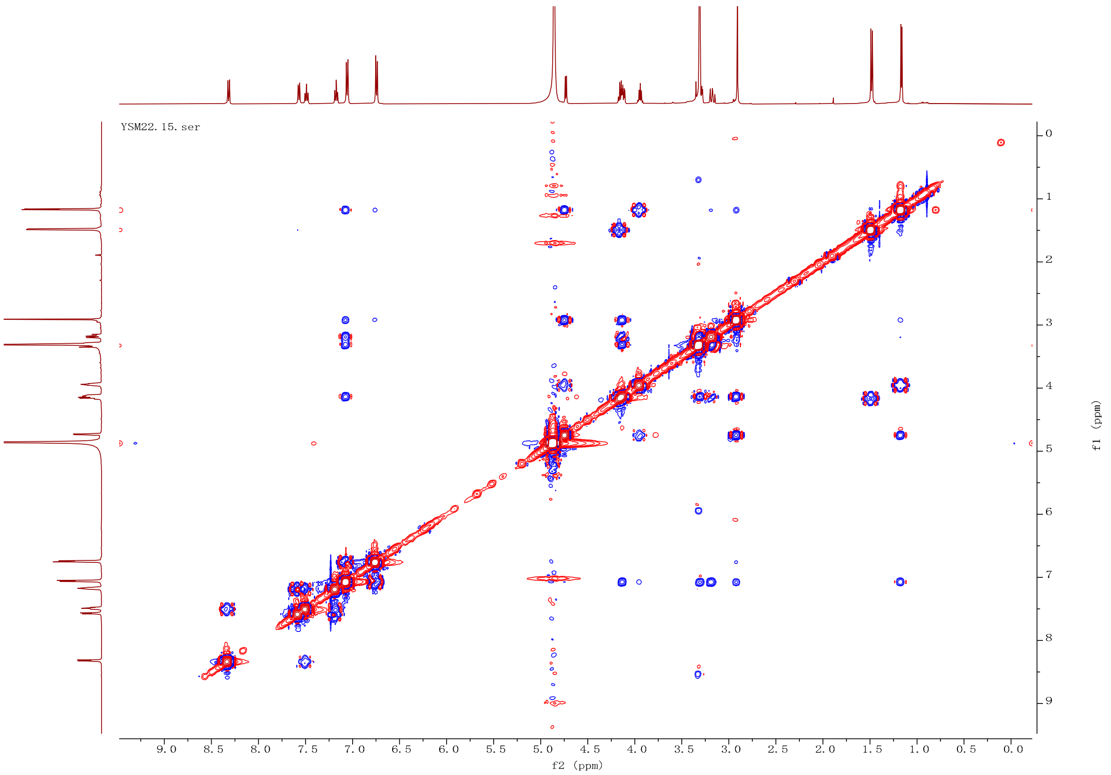

Figure S16 HR-ESIMS spectrum of 2

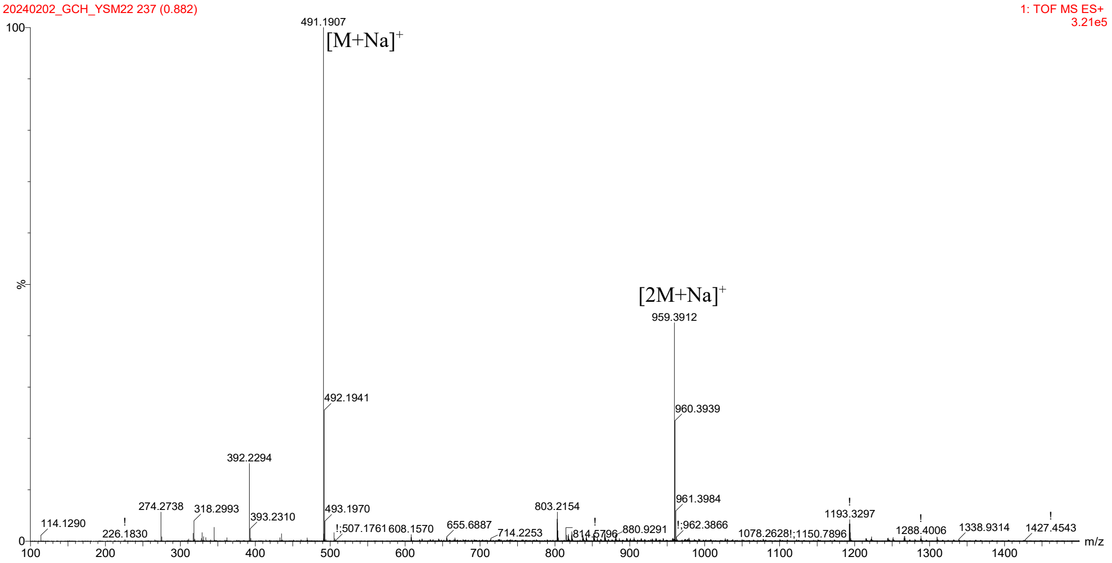

Figure S17 ^{1}H NMR spectrum of 3 in methanol-d_4

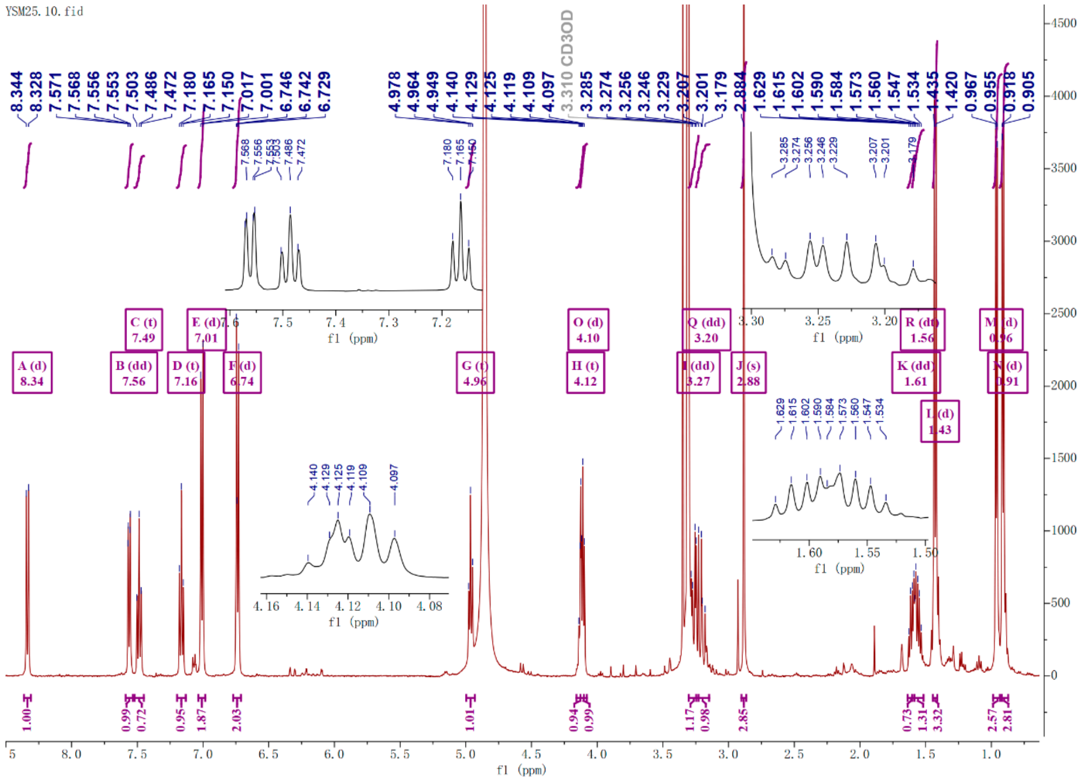

Figure S18 ^{13}C NMR spectrum of 3 in methanol-d_4

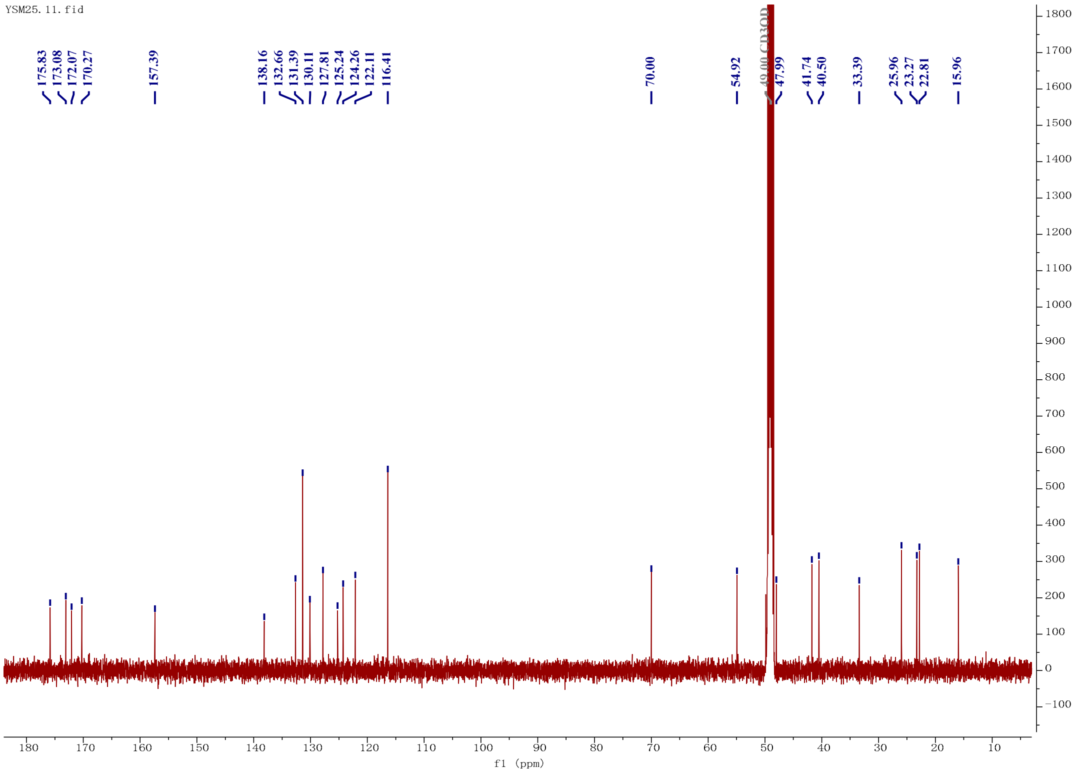

Figure S19 DEPT135 spectrum of 3 in methanol-d_4

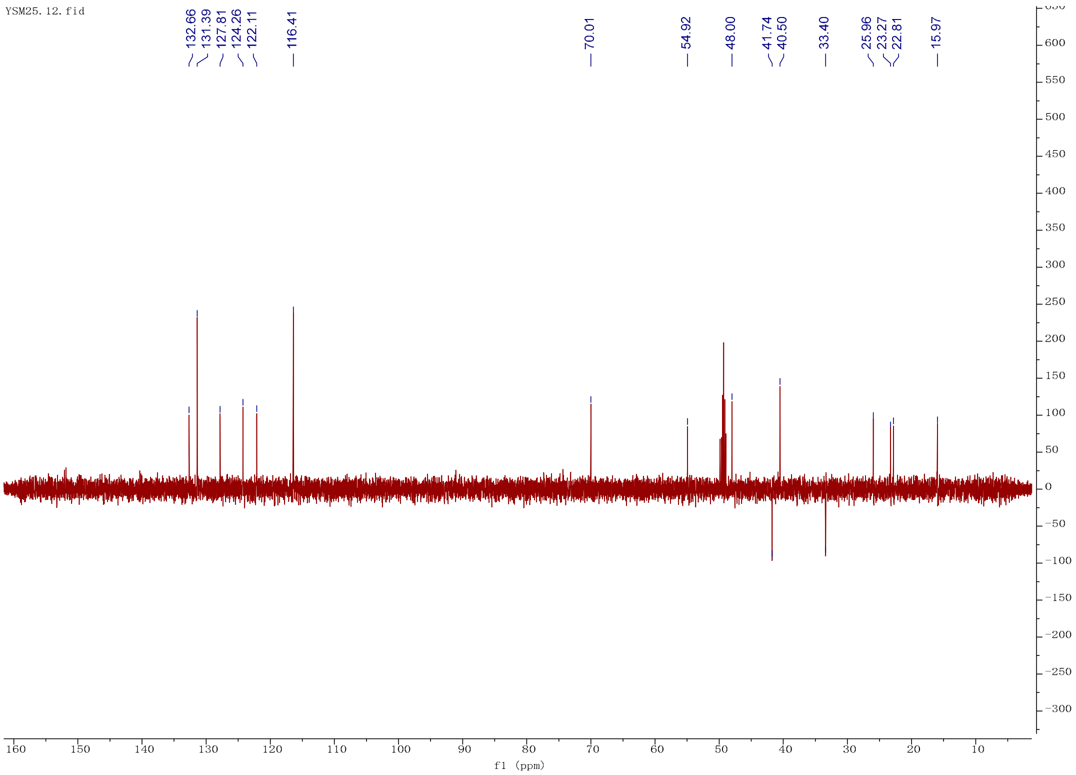

Figure S20 ^{1}H-^{1}H COSY spectrum of 3 in methanol-d_4

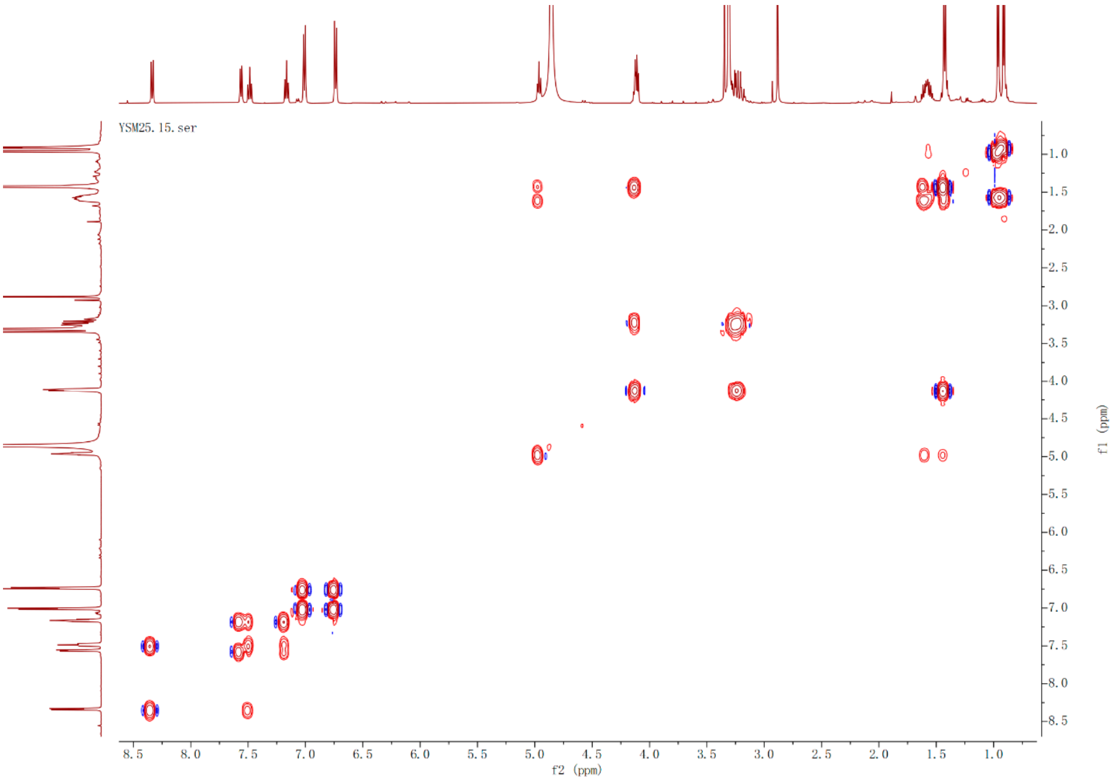

Figure S21 HSQC spectrum of 3 in methanol-d_4

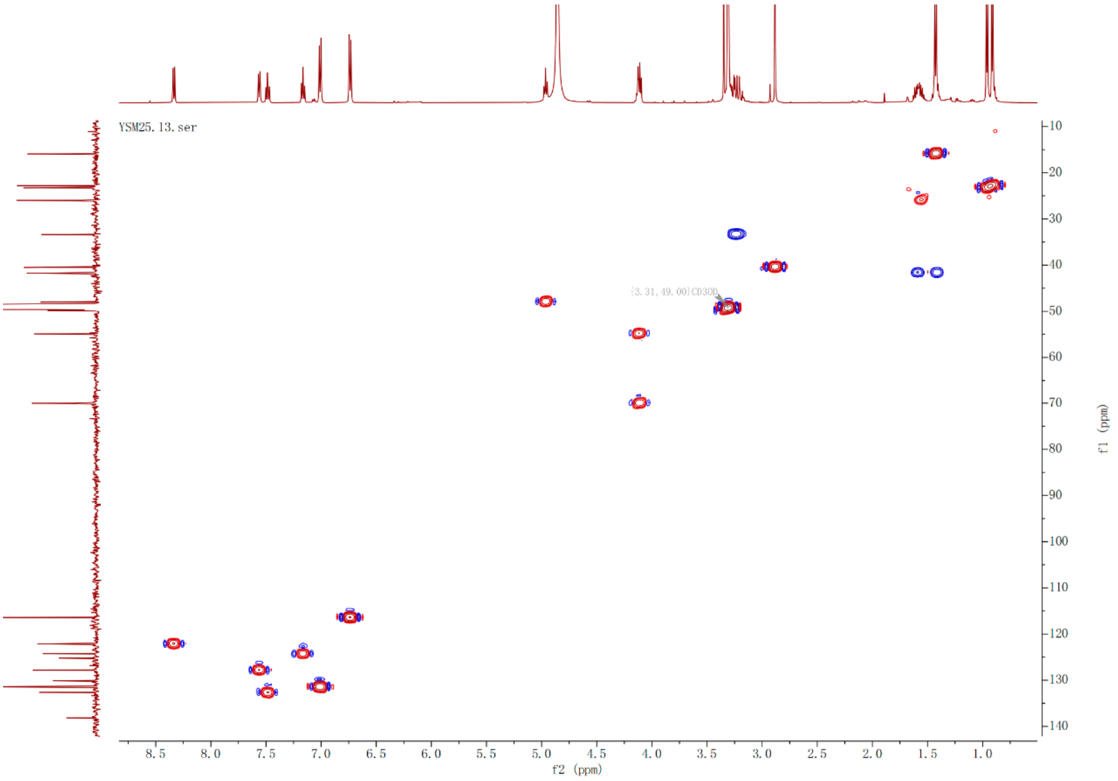

Figure S22 HMBC spectrum of 3 in methanol-d_4

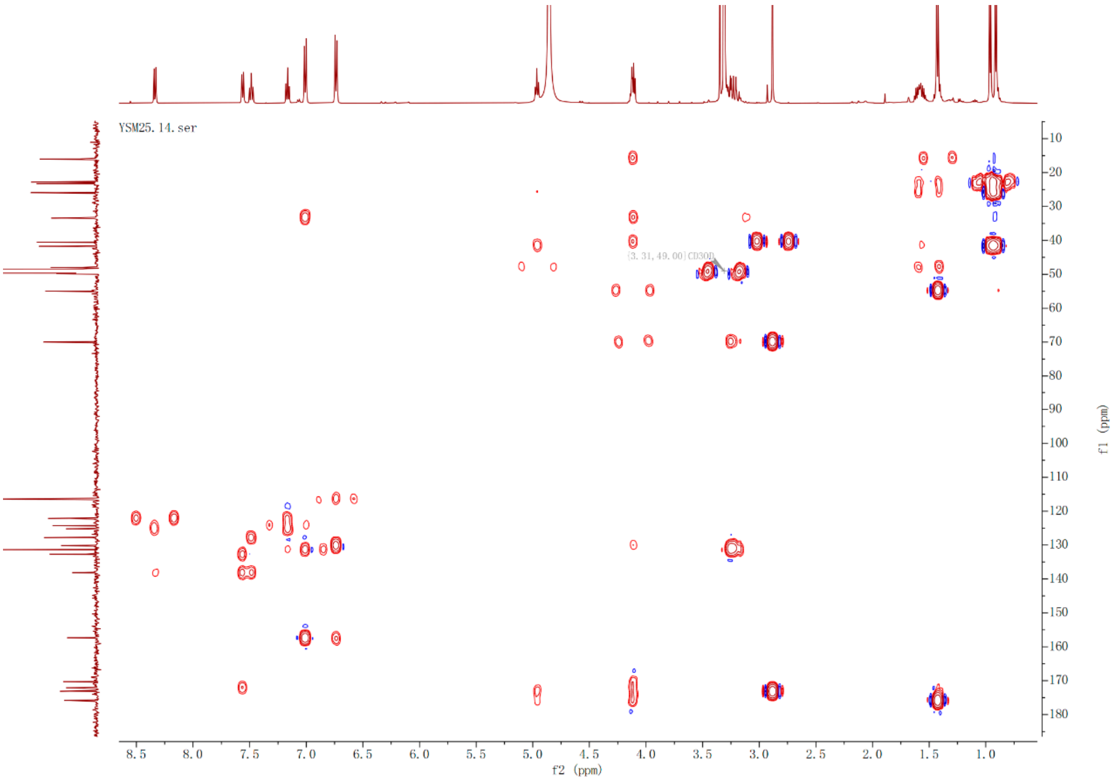

Figure S23 NOESY spectrum of 3 in methanol-d_4

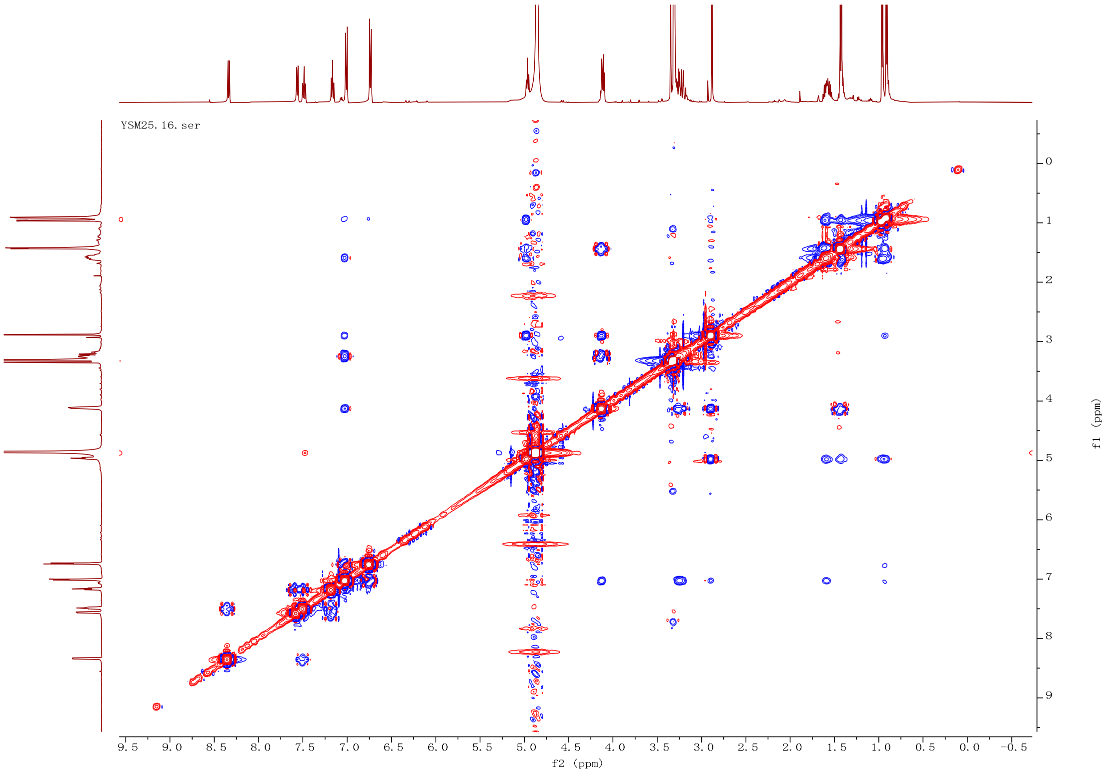

Figure S24 HR-ESIMS spectrum of 3

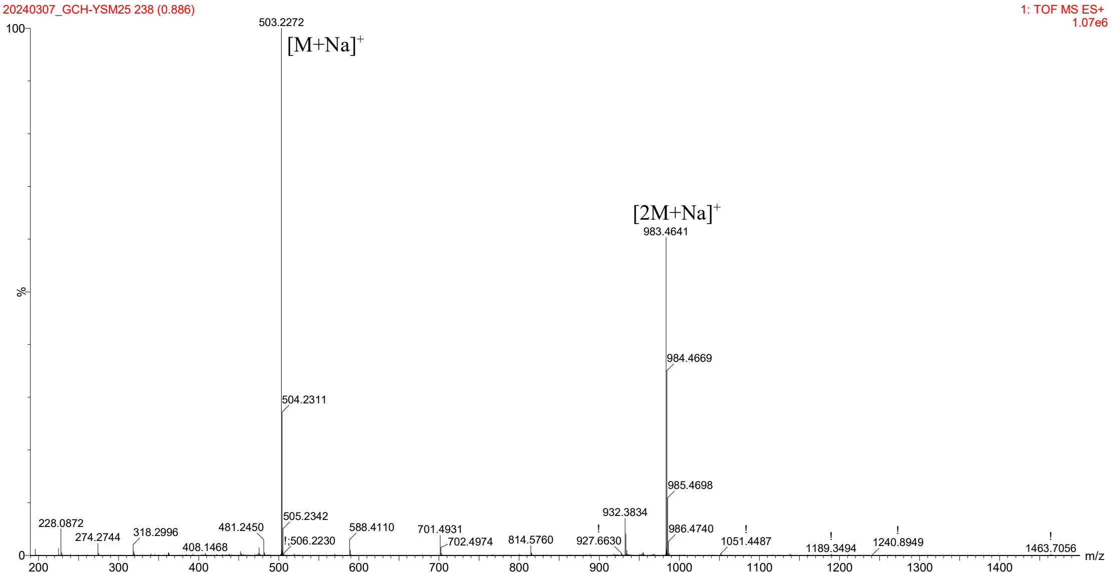

Figure S25 ^{1}H NMR spectrum of 6 in methanol-d_4

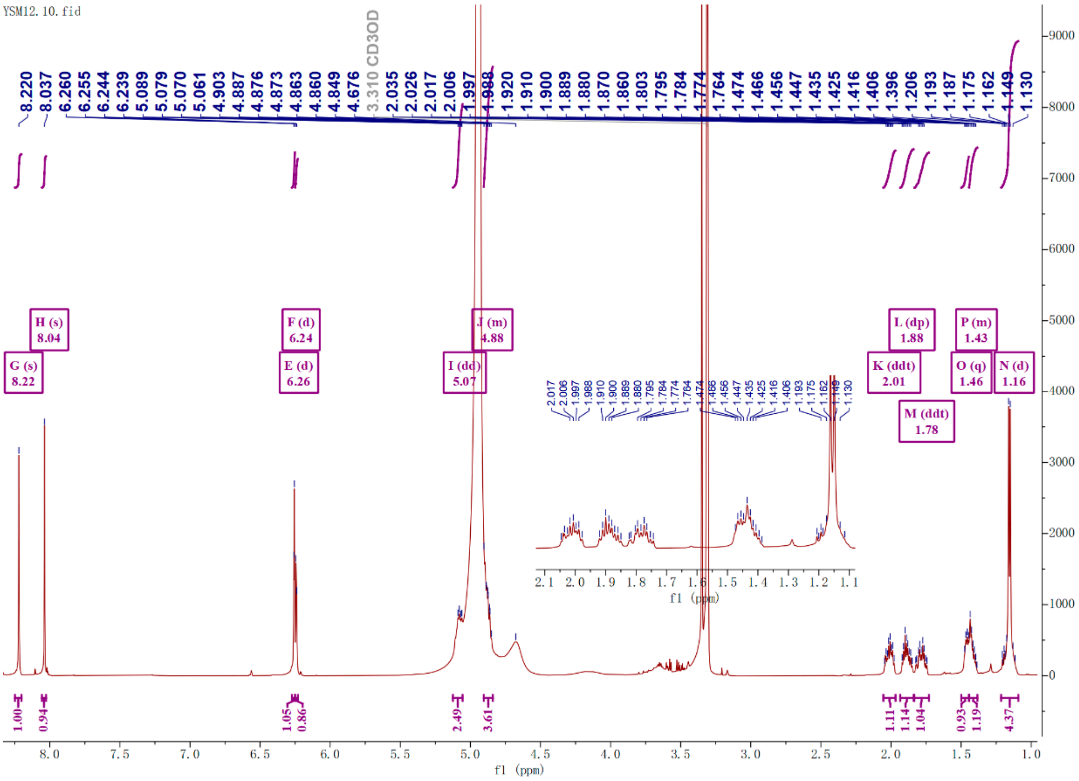

Figure S26 ^{13}C NMR spectrum of 6 in methanol-d_4

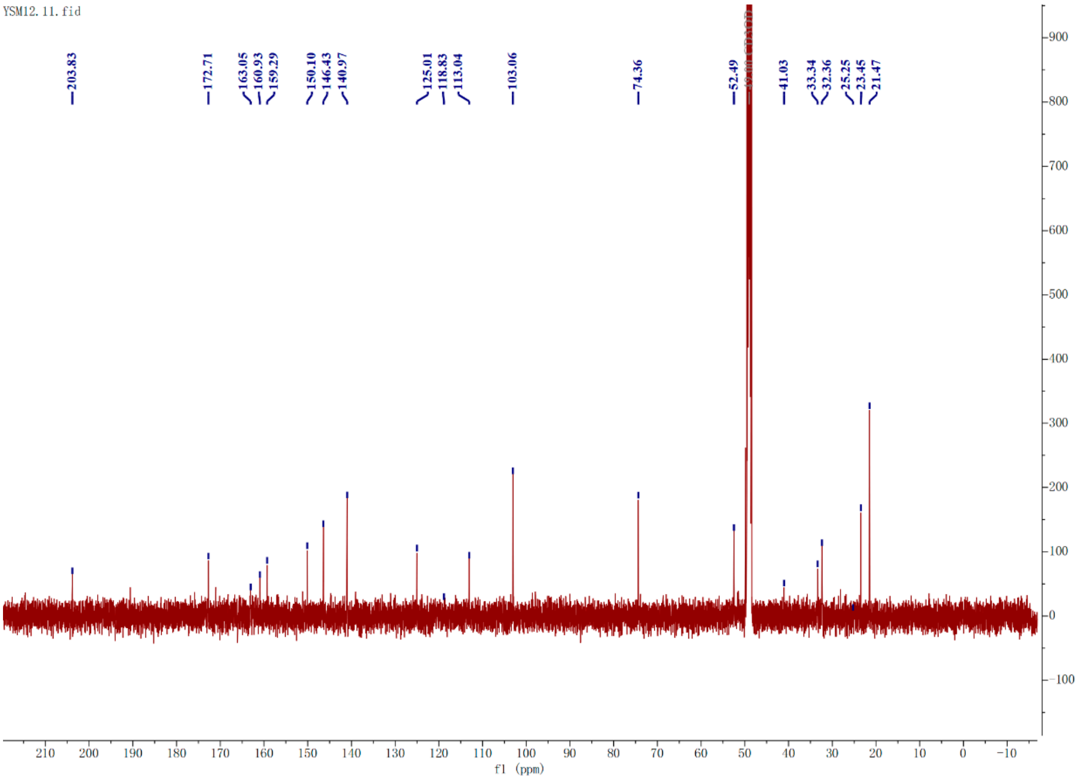

Figure S27 DEPT135 spectrum of 6 in methanol-d_4

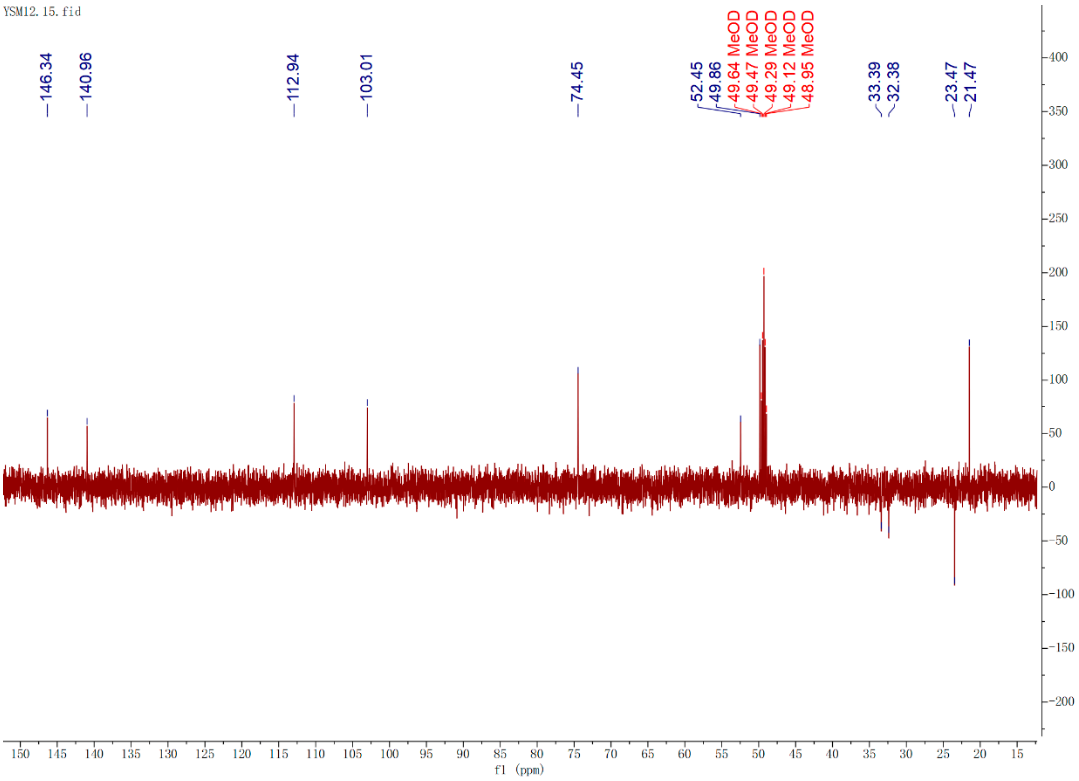

Figure S28 HSQC spectrum of 6 in methanol-d_4

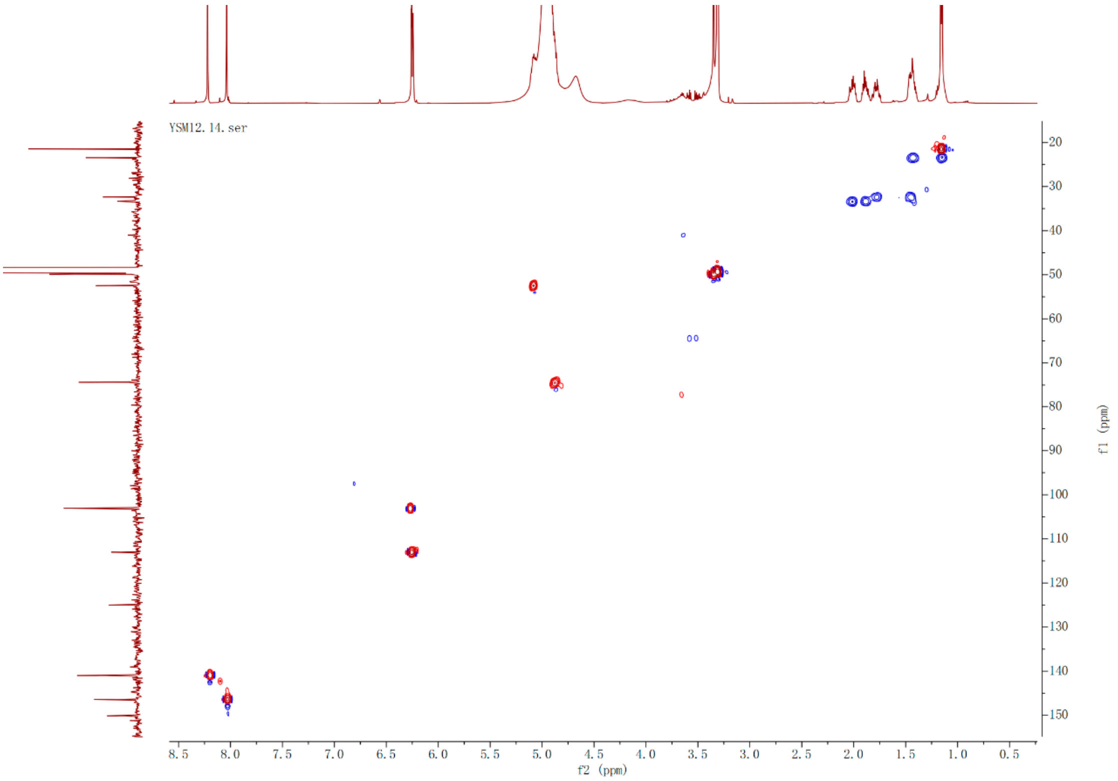

Figure S29 HMBC spectrum of 6 in methanol-d_4

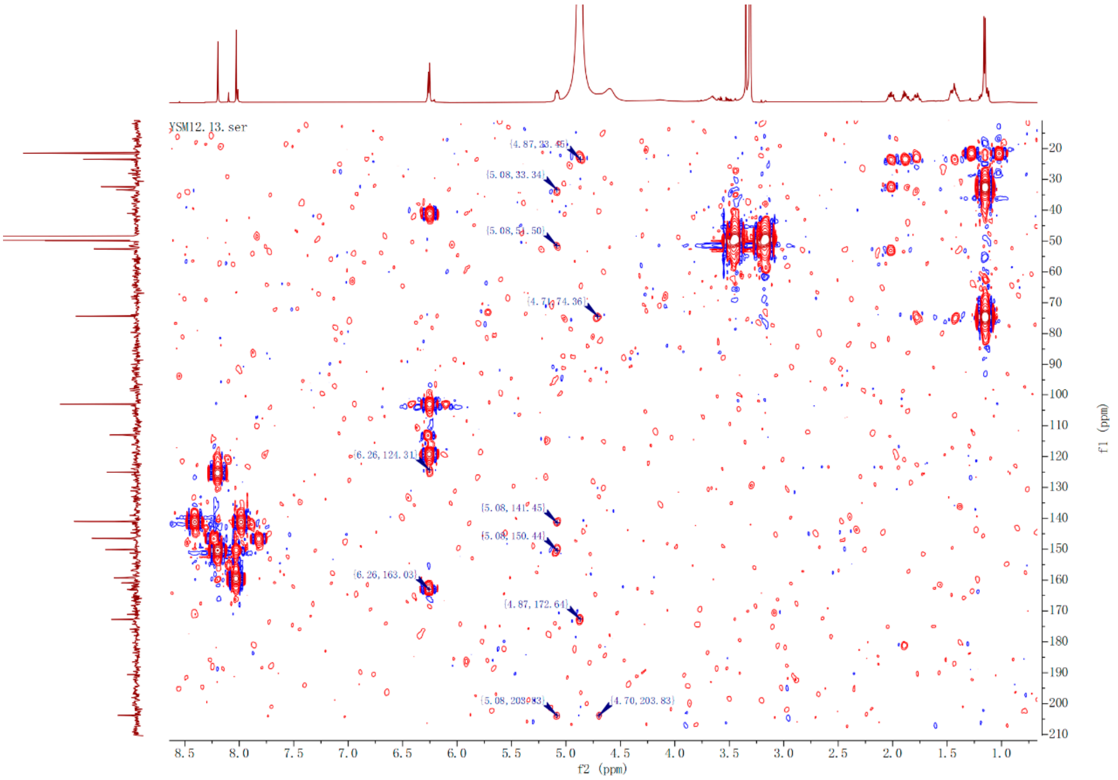

Figure S30 HR-ESIMS spectrum of 6

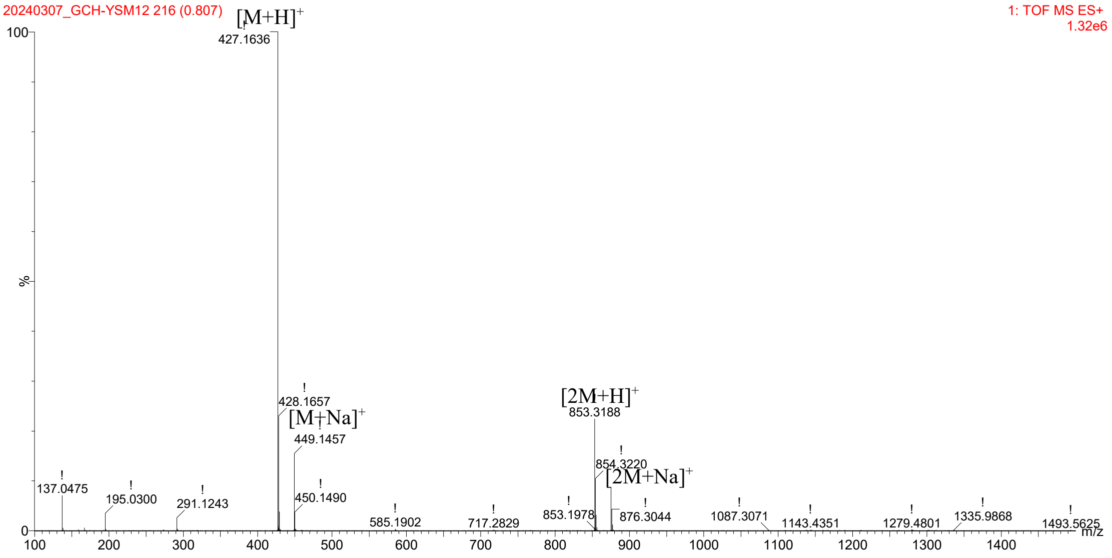

Figure S31 ^{1}H NMR spectrum of 7 in methanol-d_4

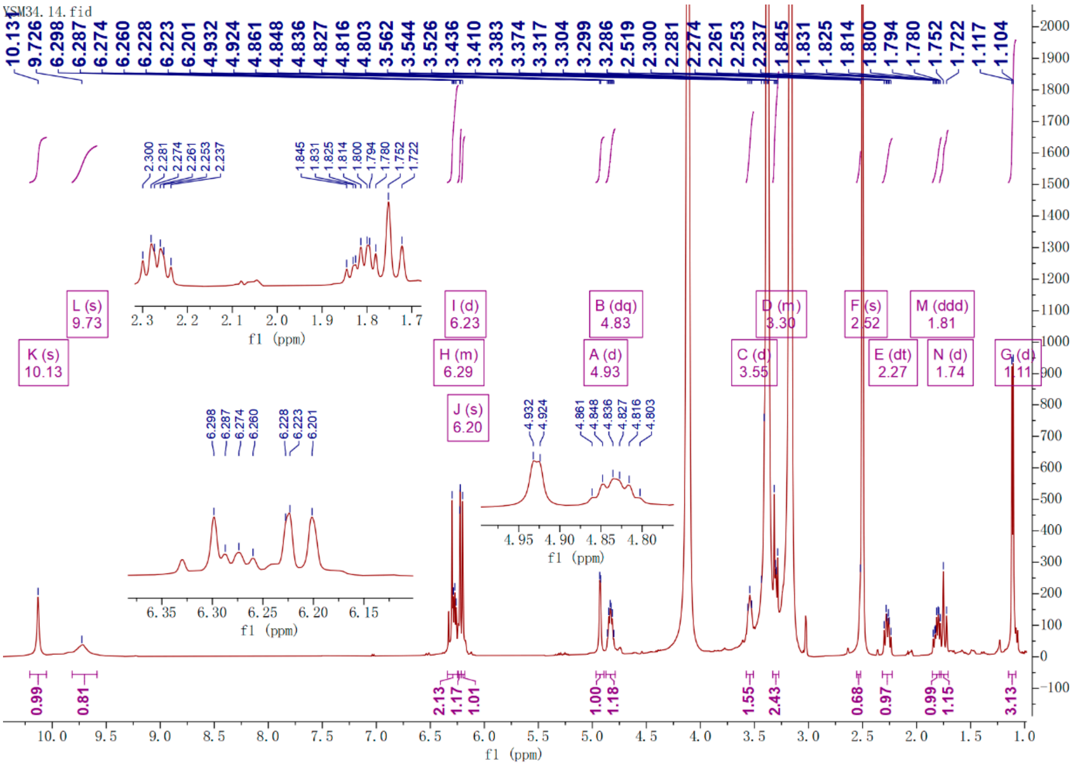

Figure S32 ^{13}C NMR spectrum of 7 in methanol-d_4

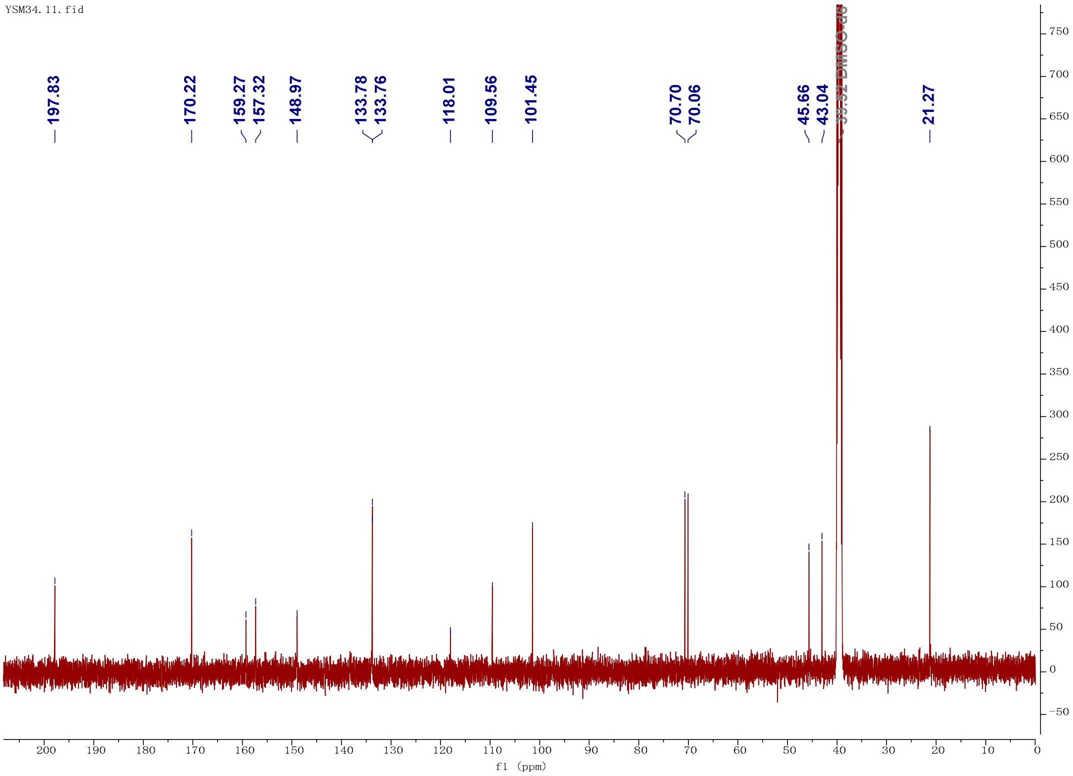

Figure S33 DEPT135 spectrum of 7 in methanol-d_4

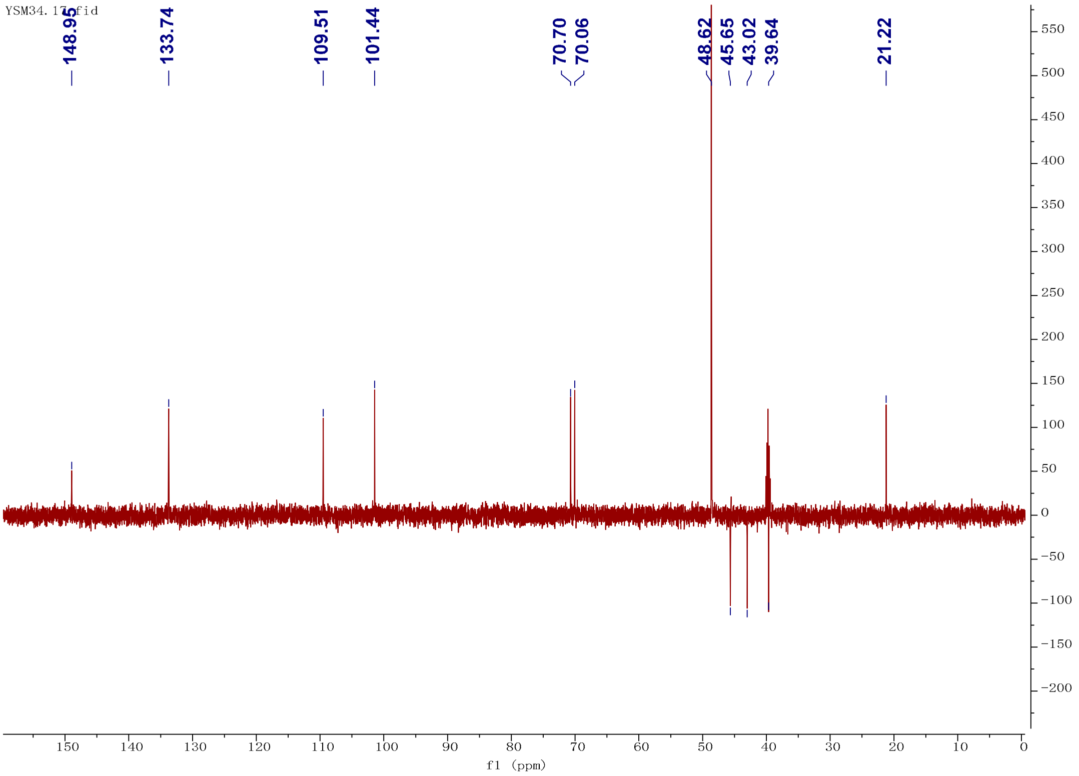

Figure S34 ^{1}H-^{1}H COSY spectrum of 7 in methanol-d_4

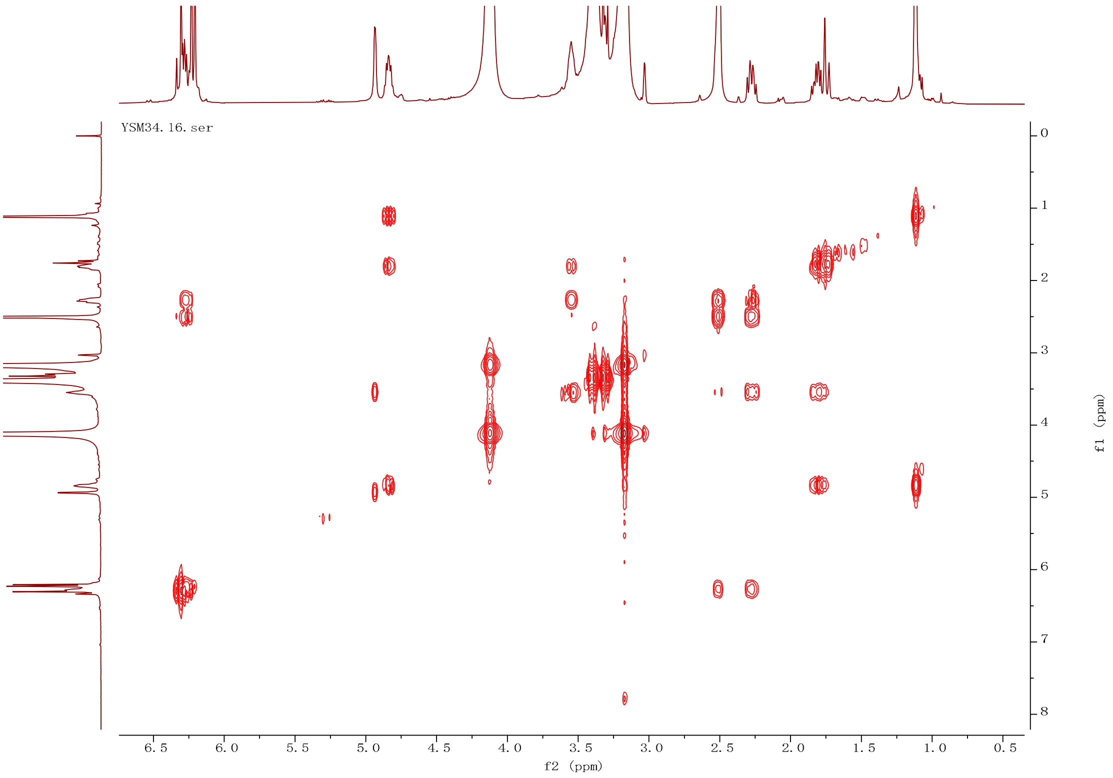

Figure S35 HSQC spectrum of 7 in methanol-d_4

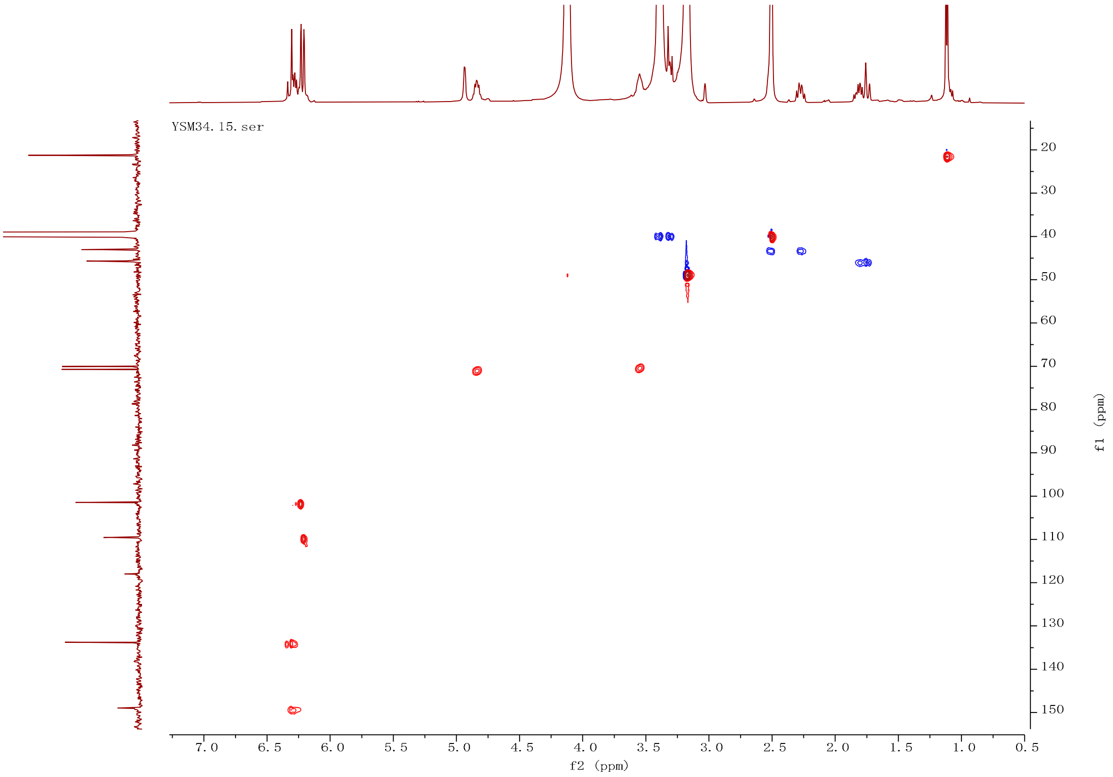

Figure S36 HMBC spectrum of 7 in methanol-d_4

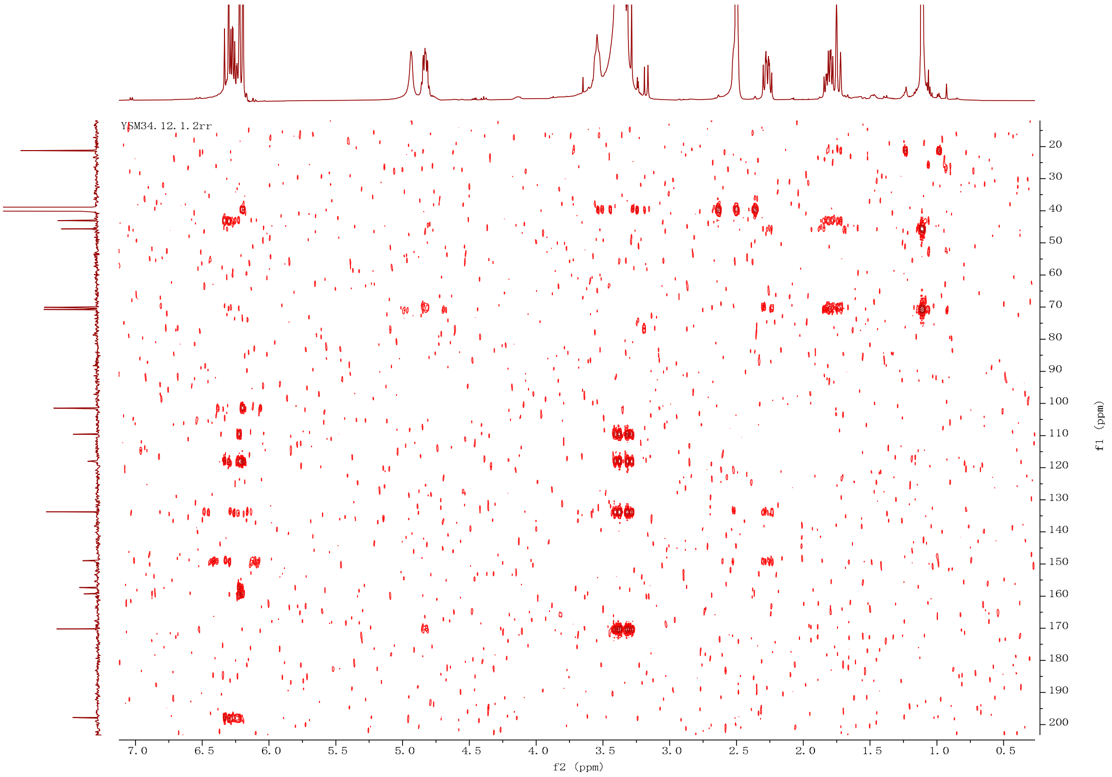

Figure S37 NOESY spectrum of 7 in methanol-d_4

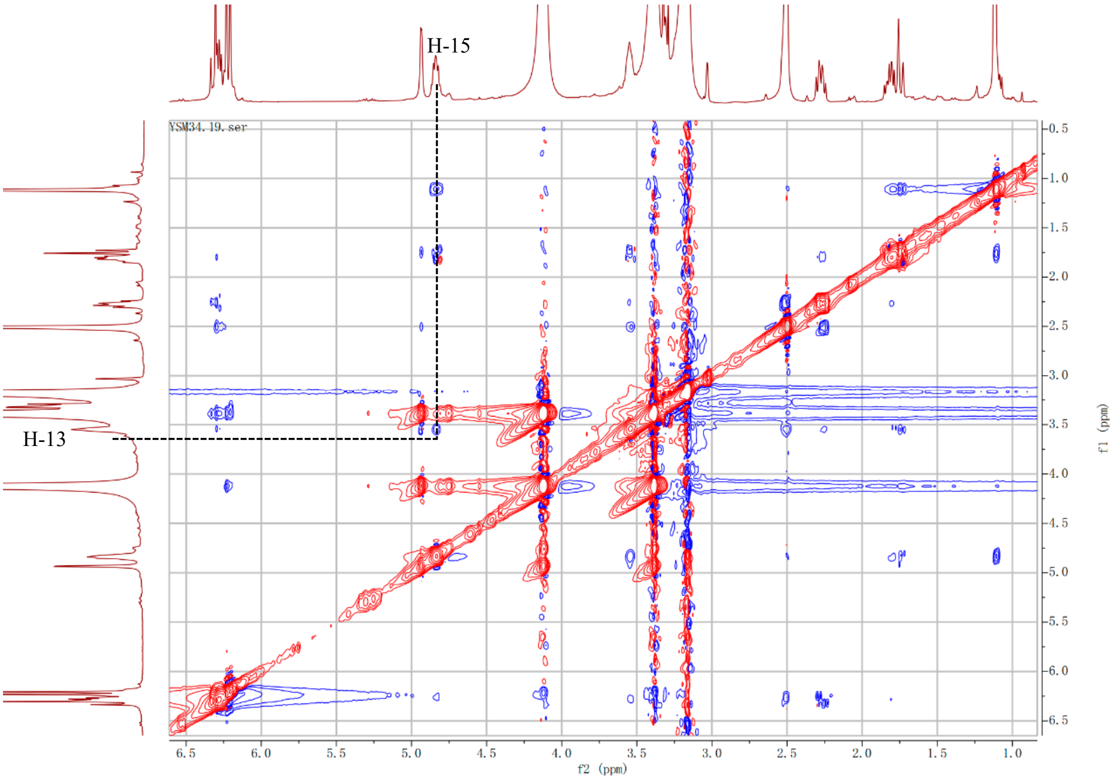

Figure S38 HR-ESIMS spectrum of 7

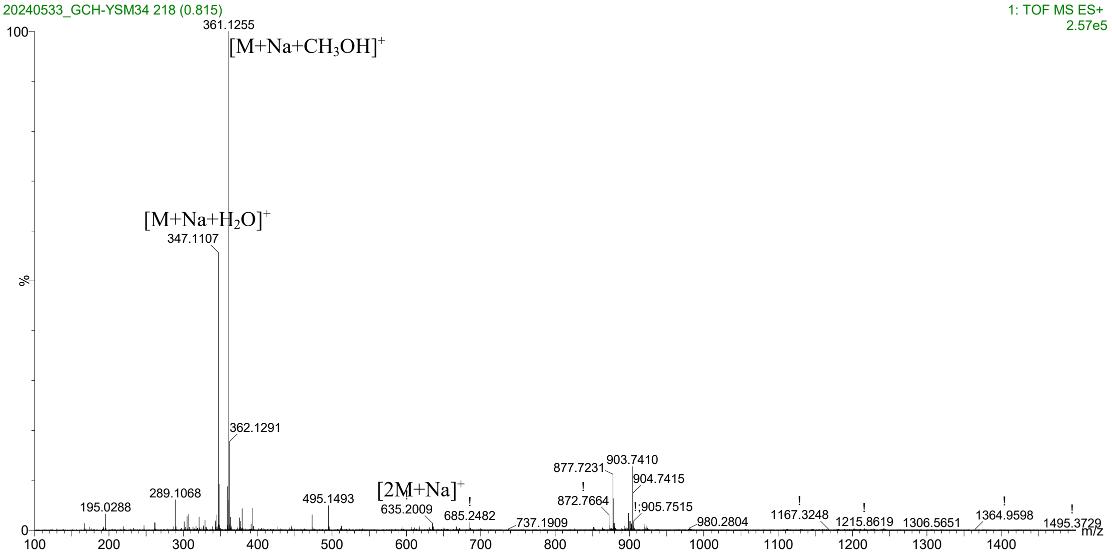

Figure S39 ^{1}H NMR spectra of 7 and (+)-(10*E*,15*R*)-13-Hydroxy-10,11-dehydrocurvularin in acetone-d_6

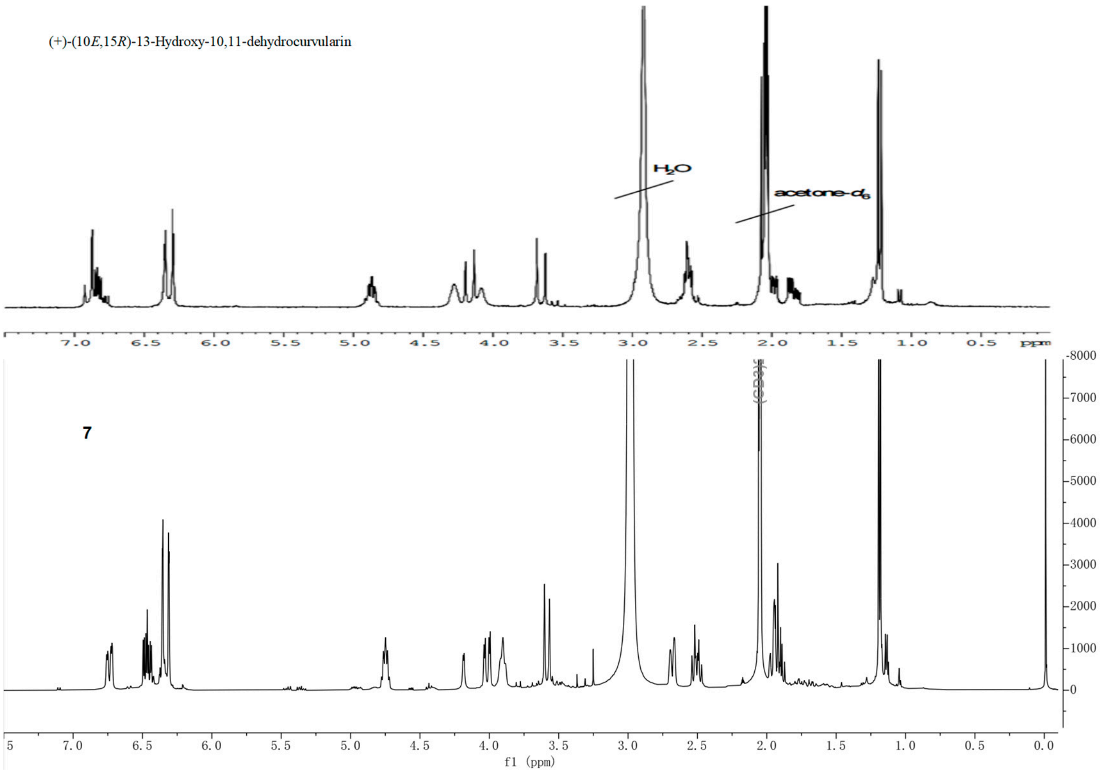

Figure S40 Comparison the ^{1}H NMR spectra of 8 and (+)-(10*E*,15*R*)-12-Hydroxy-10,11-dehydrocurvularin in acetone-d_6

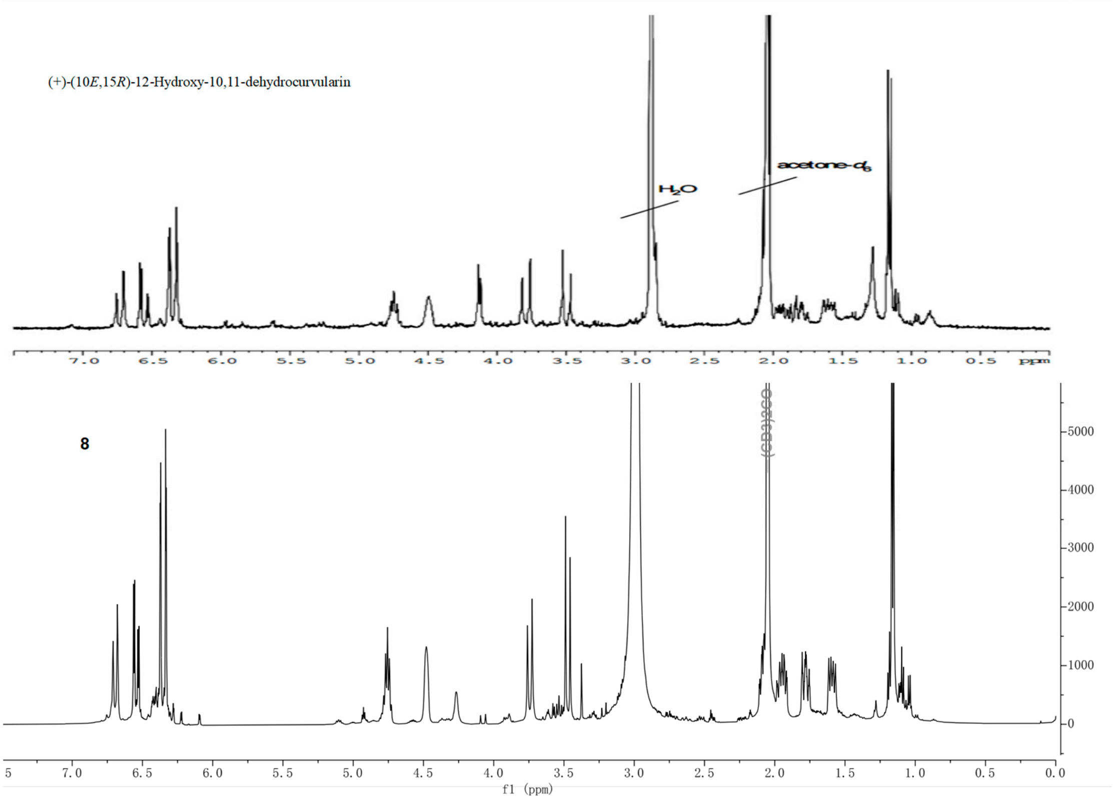

Figure S41 ^{1}H NMR spectrum of 4 in methanol-d_4

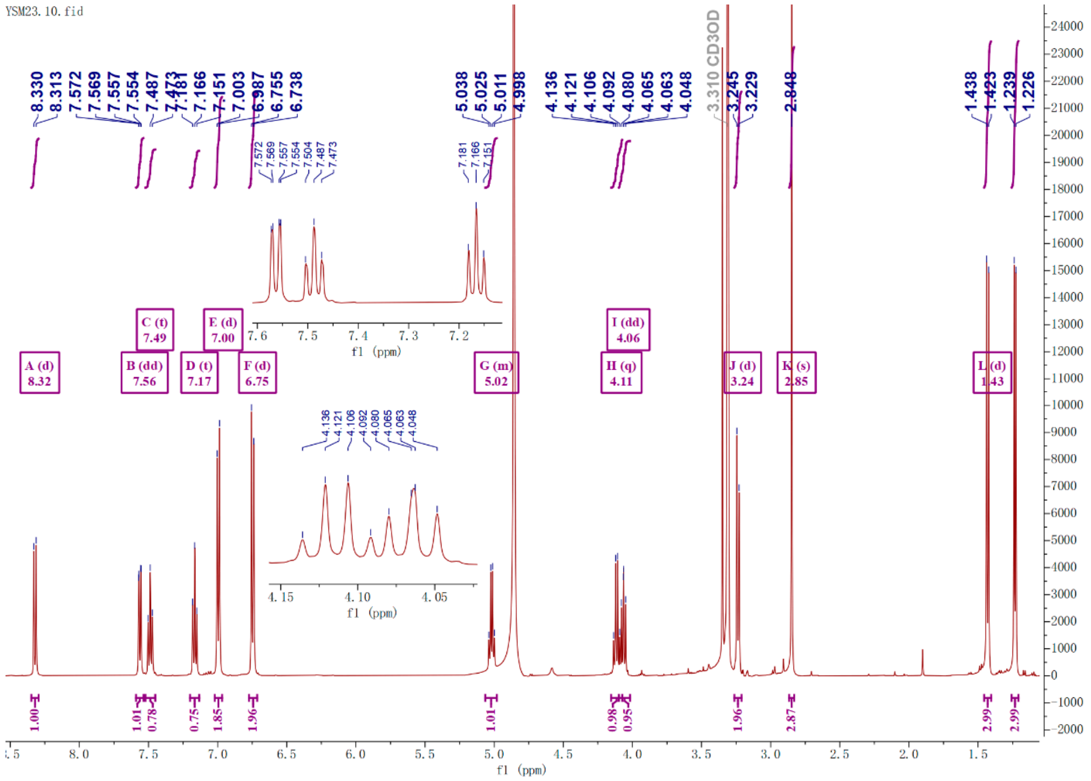

Figure S42 ^{13}C NMR spectrum of 4 in methanol-d_4

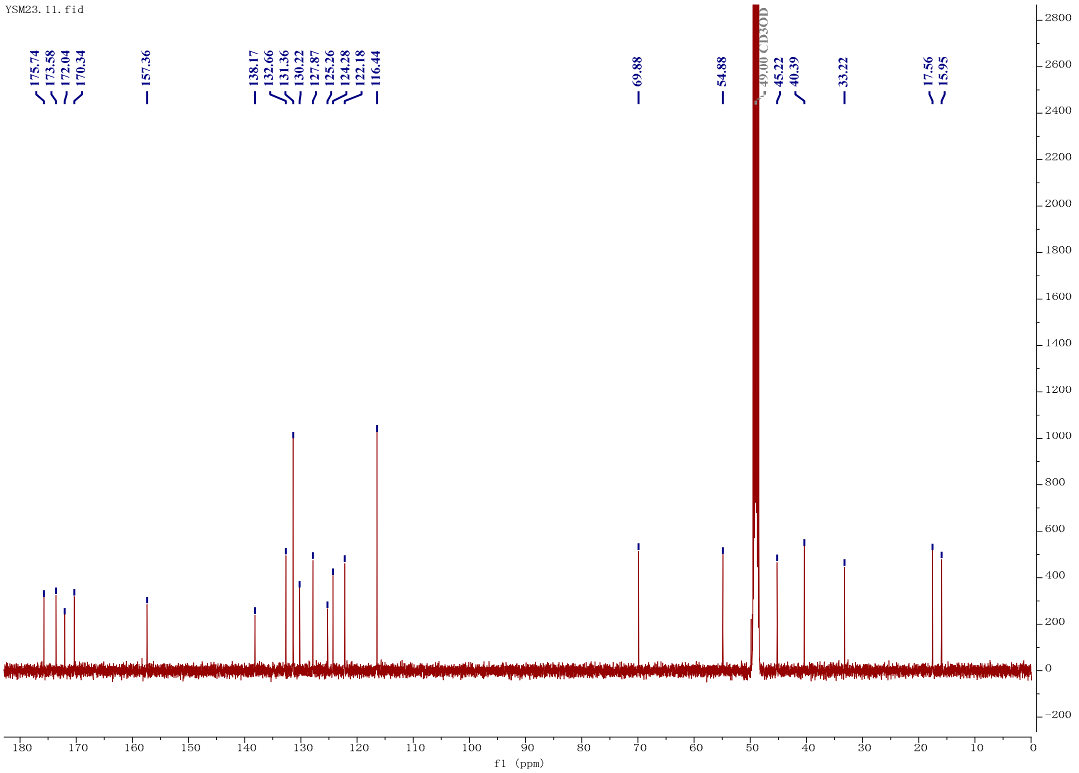

Figure S43 DEPT135 spectrum of 4 in methanol-d_4

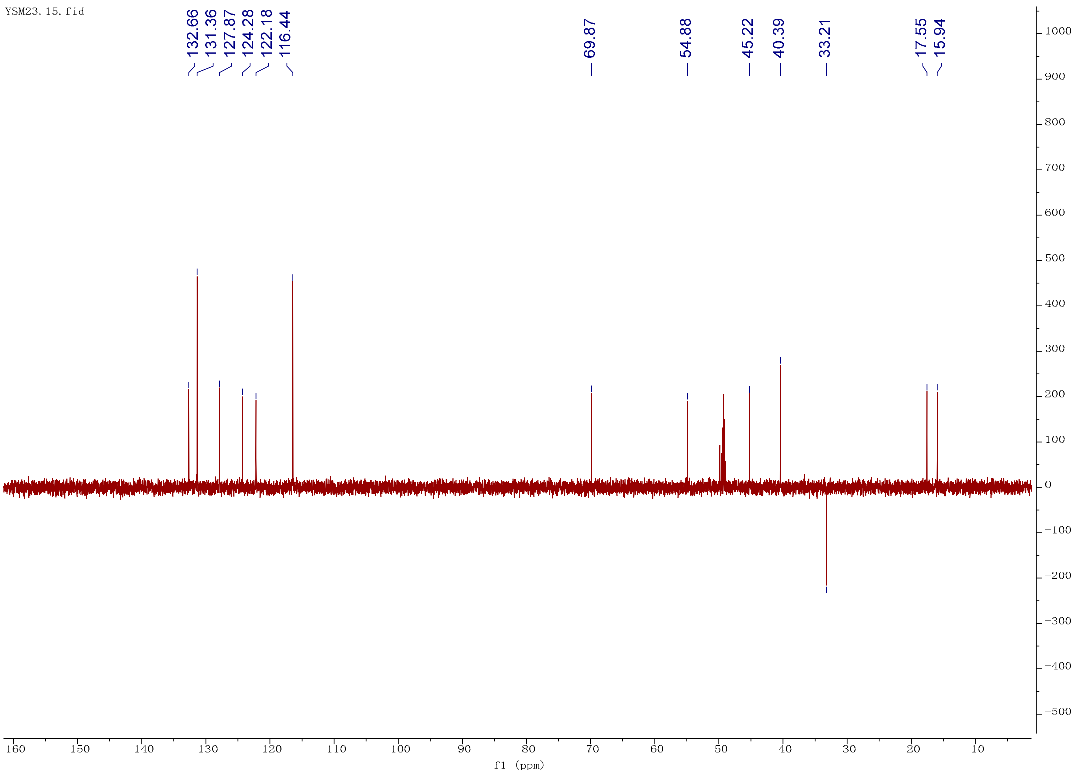

Figure S44 ^{1}H-^{1}H COSY spectrum of 4 in methanol-d_4

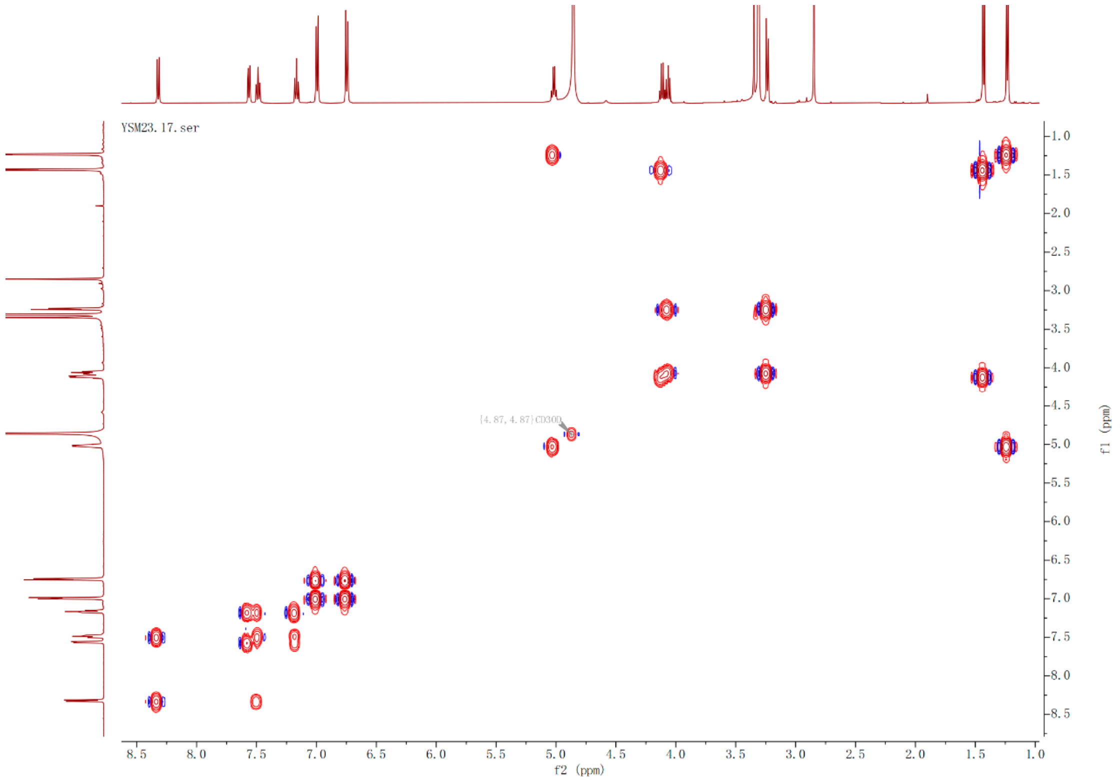

Figure S45 HSQC spectrum of 4 in methanol-d_4

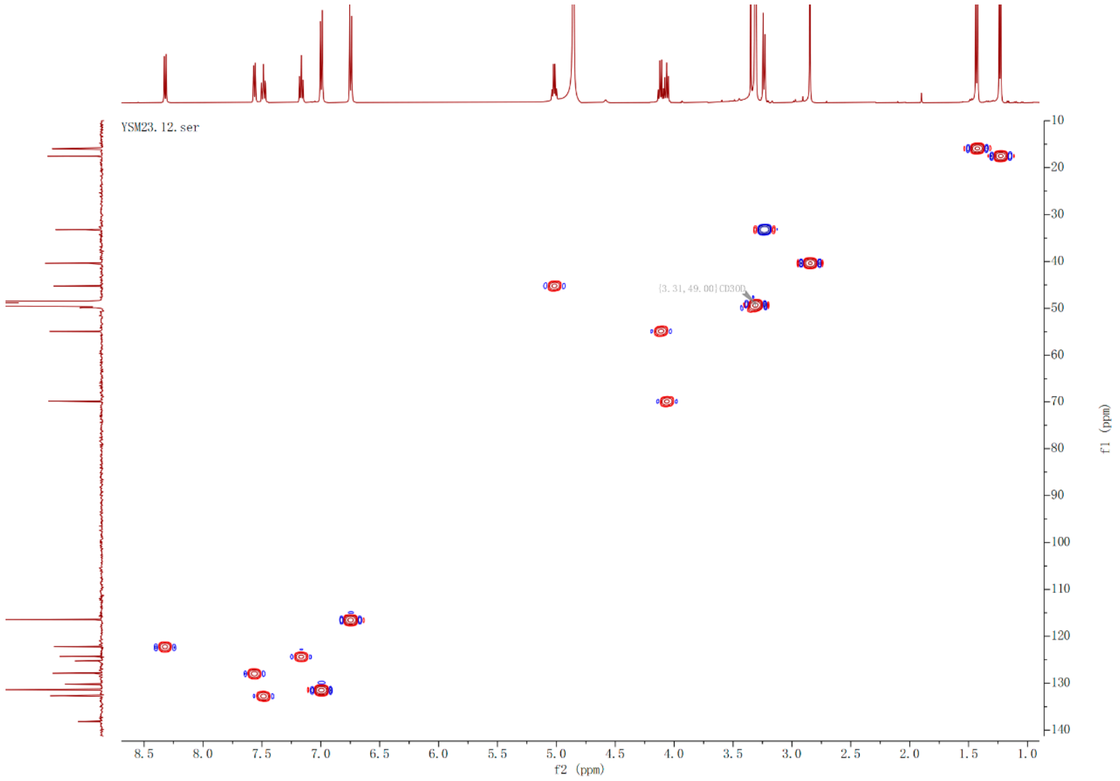

Figure S46 HMBC spectrum of 4 in methanol-d_4

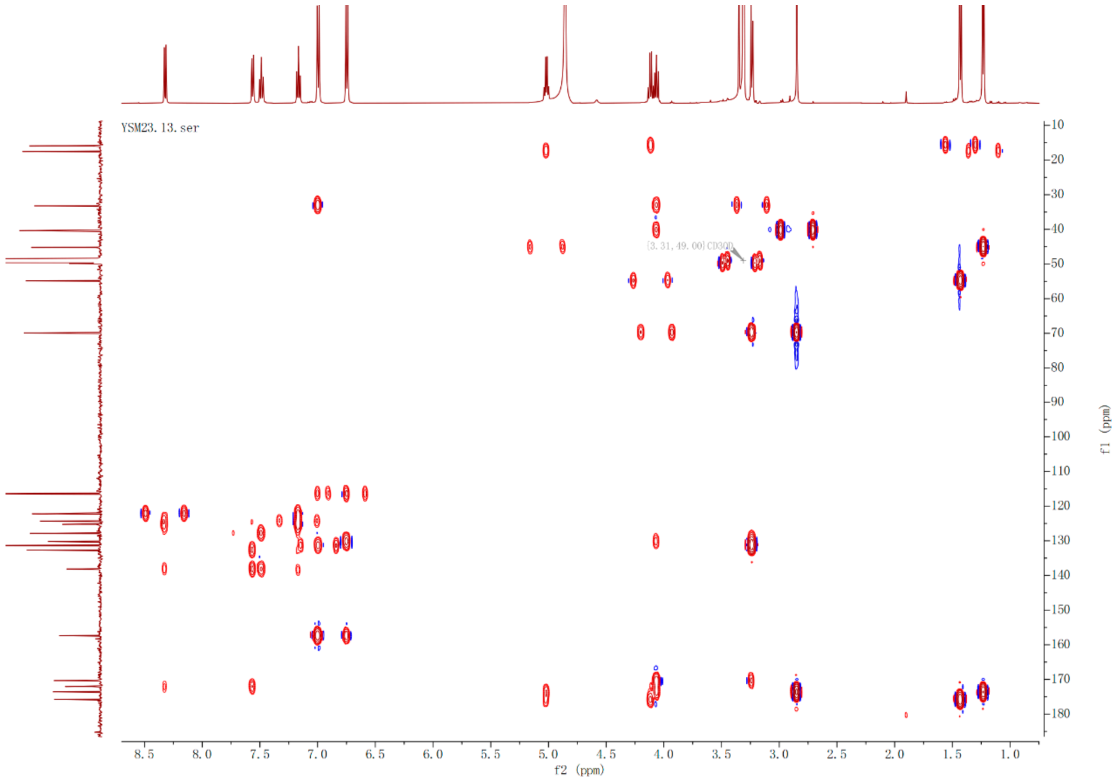

Figure S47 NOESY spectrum of 4 in methanol-d_4

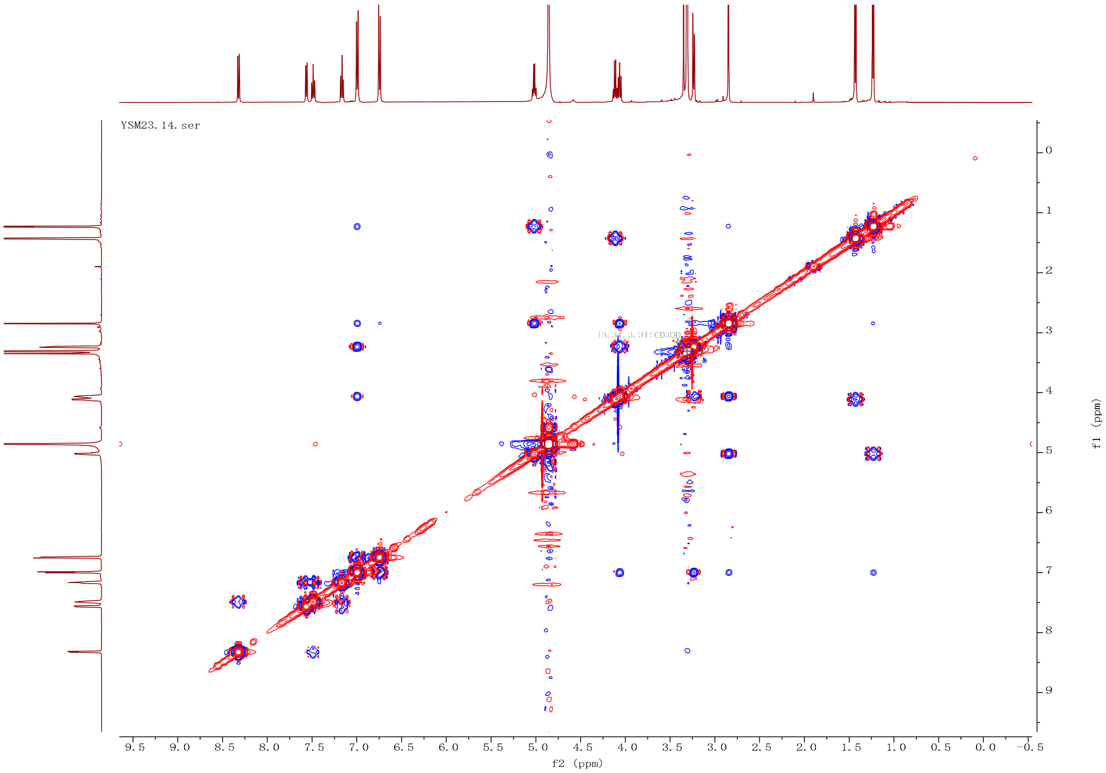

Figure S48 HR-ESIMS spectrum of 4

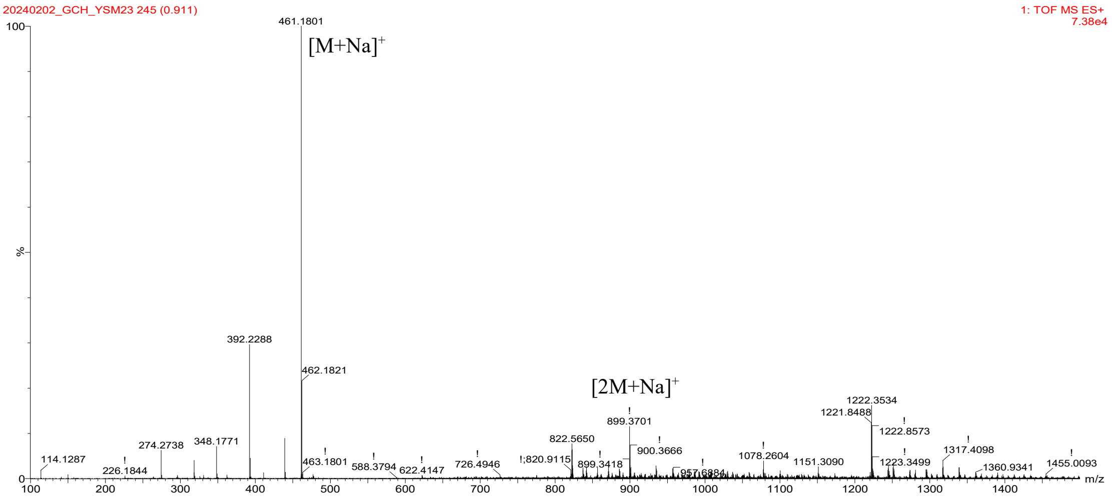

Figure S49 ^{1}H NMR spectrum of 5 in methanol-d_4

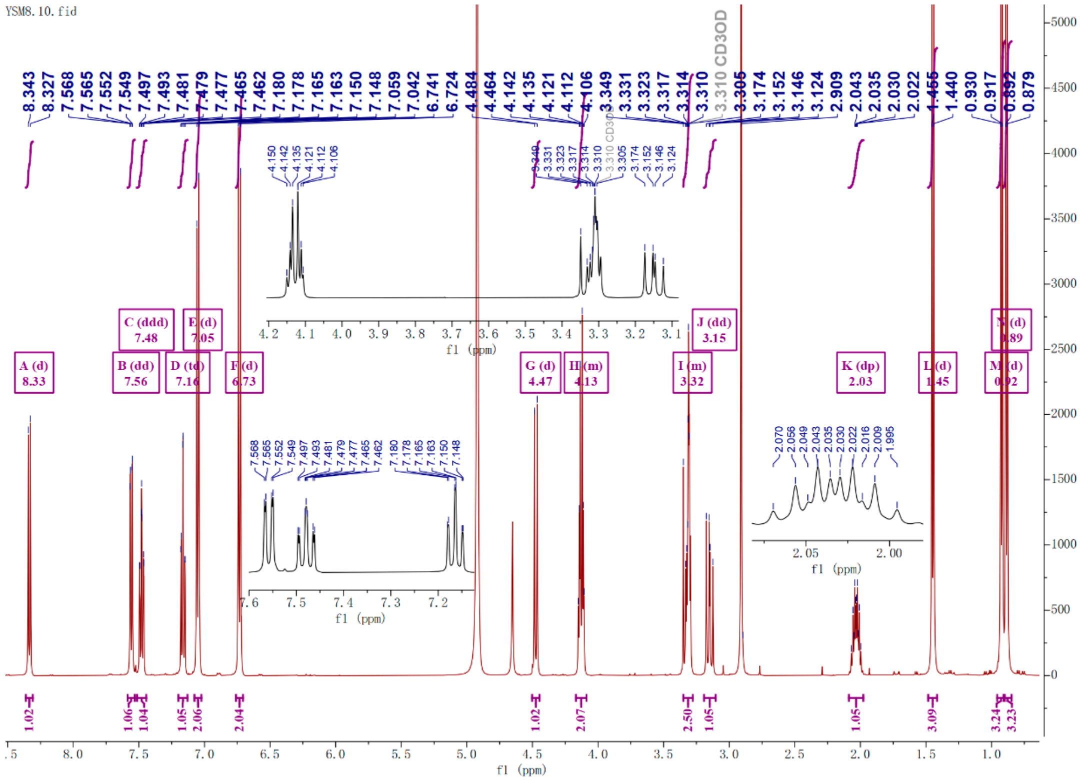

Figure S50 ^{13}C NMR spectrum of 5 in methanol-d_4

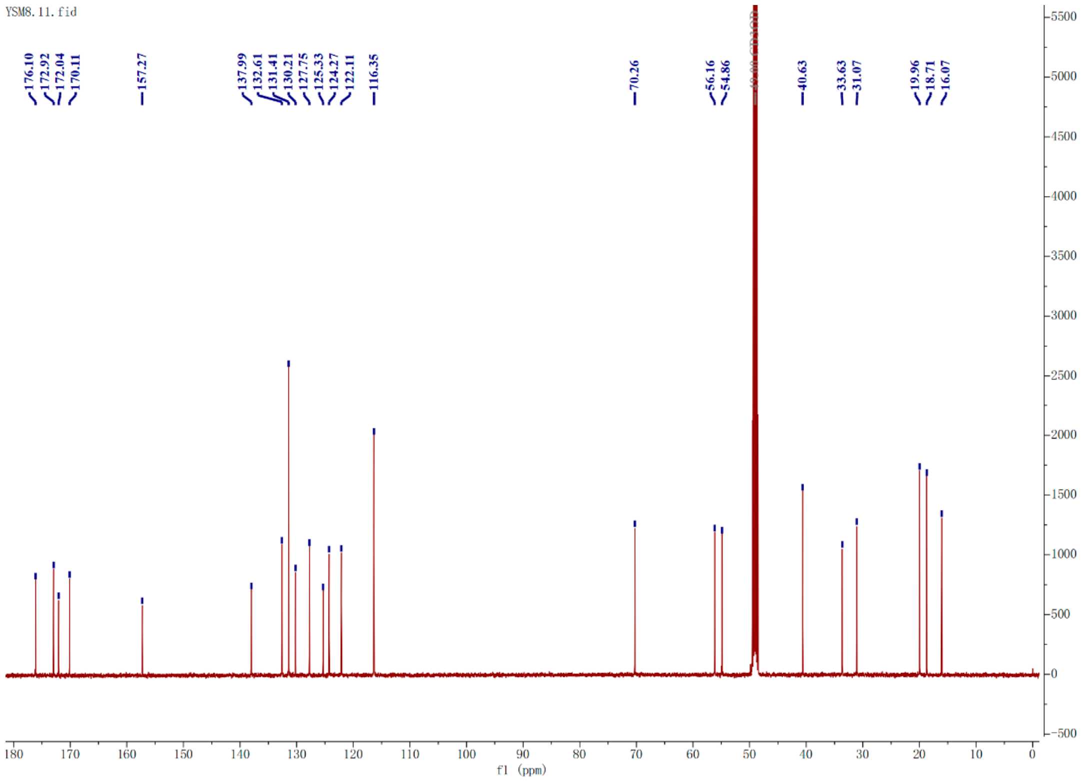

Figure S51 HR-ESIMS spectrum of 5

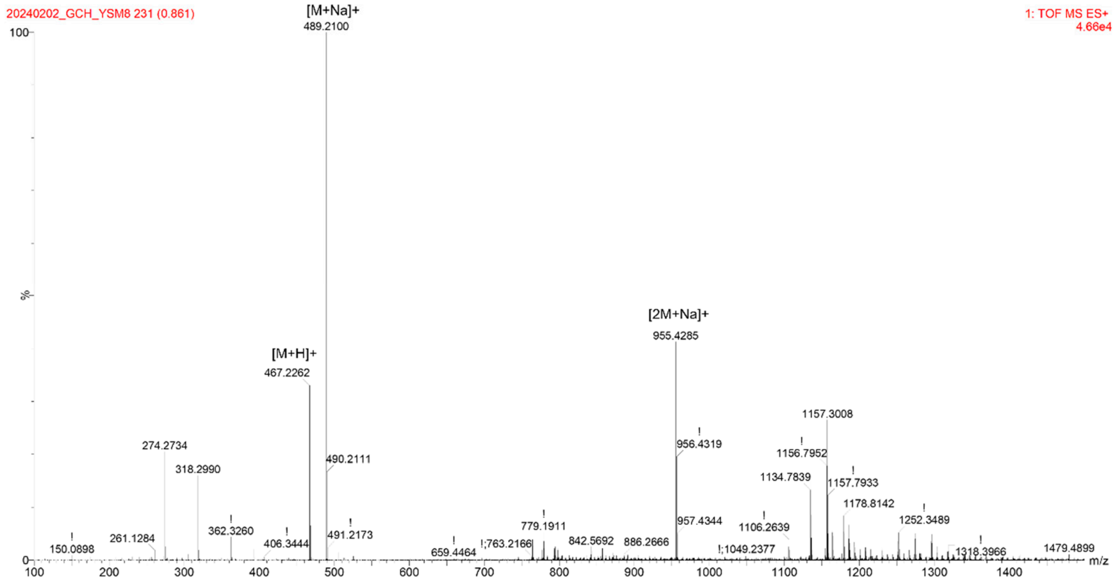

Figure S52 ^{1}H NMR spectrum of 8 in DMSO-d_6

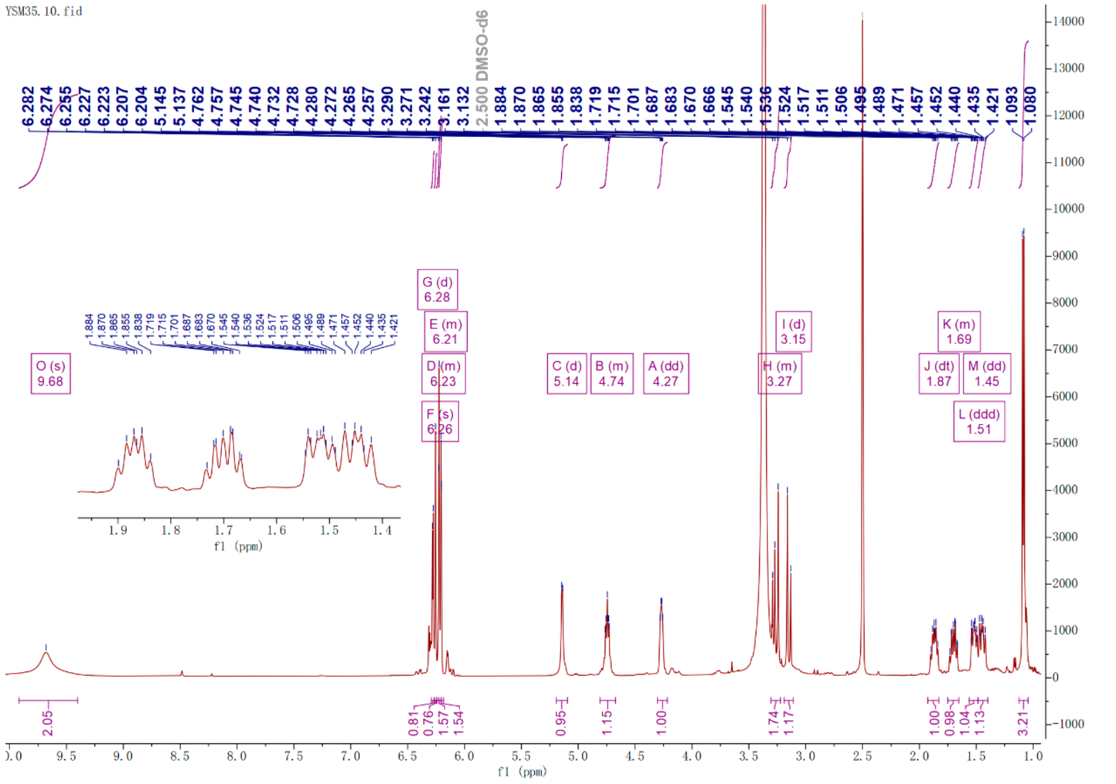

Figure S53 ^{13}C NMR spectrum of 8 in DMSO-d_6

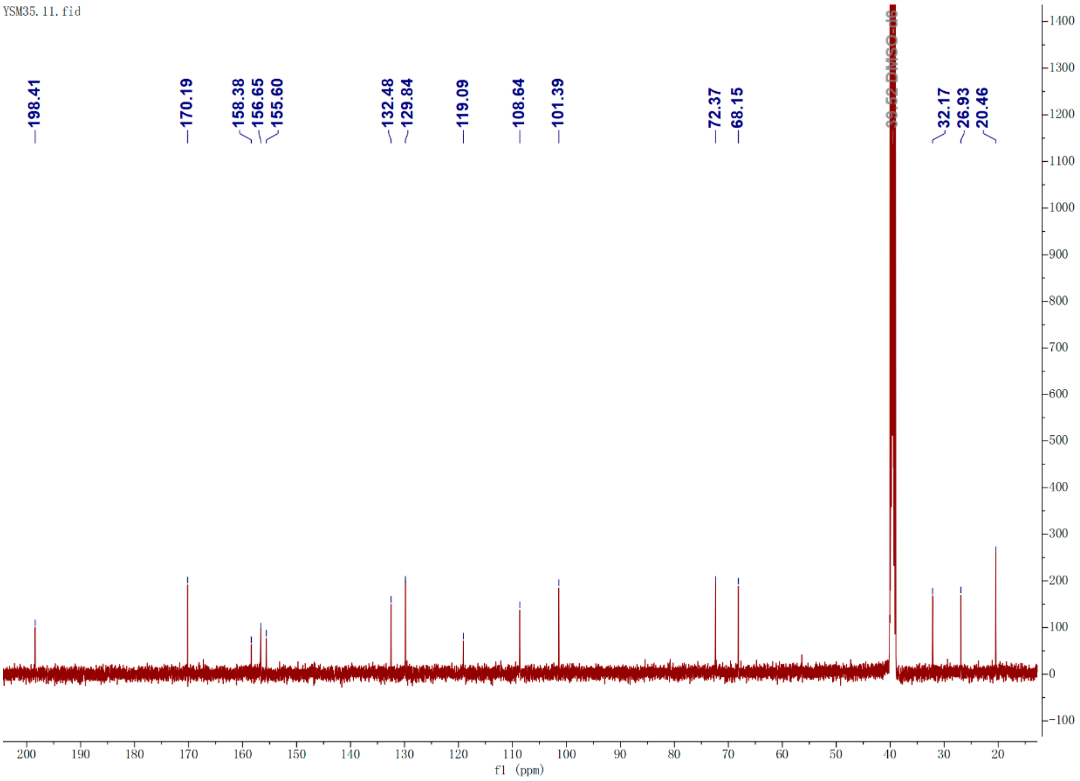

Figure S54 HMBC spectrum of 8 in DMSO-d_6

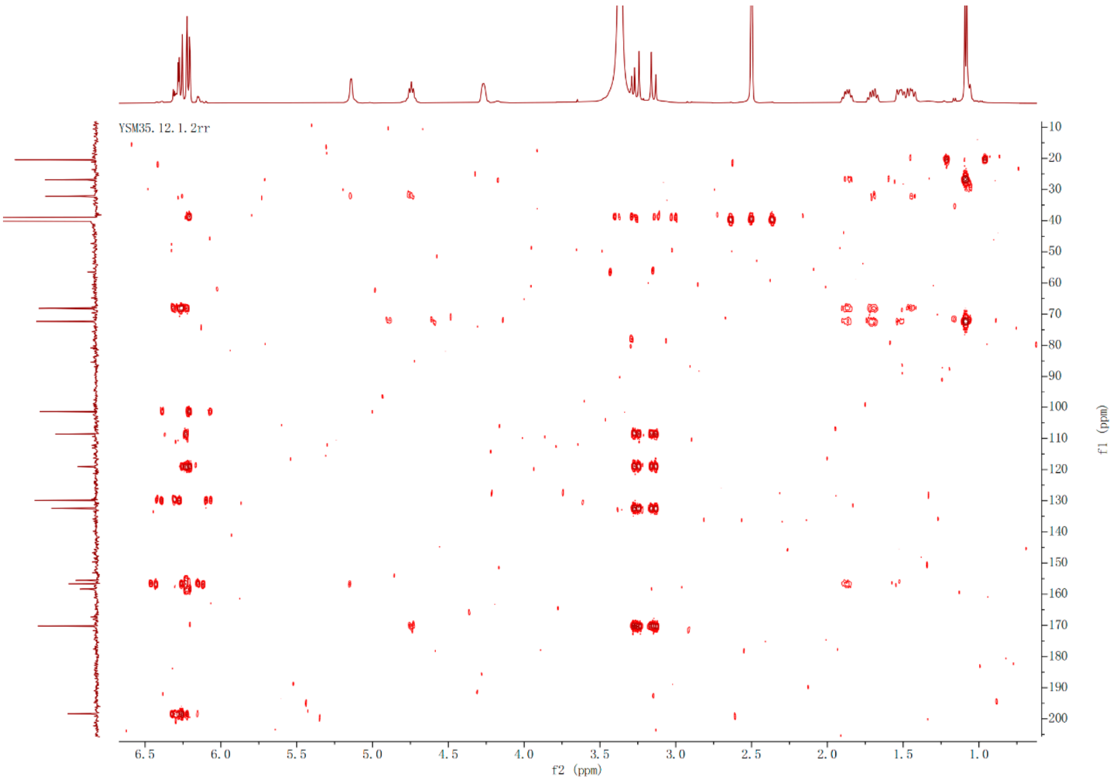

Figure S55 HR-ESIMS spectrum of 8

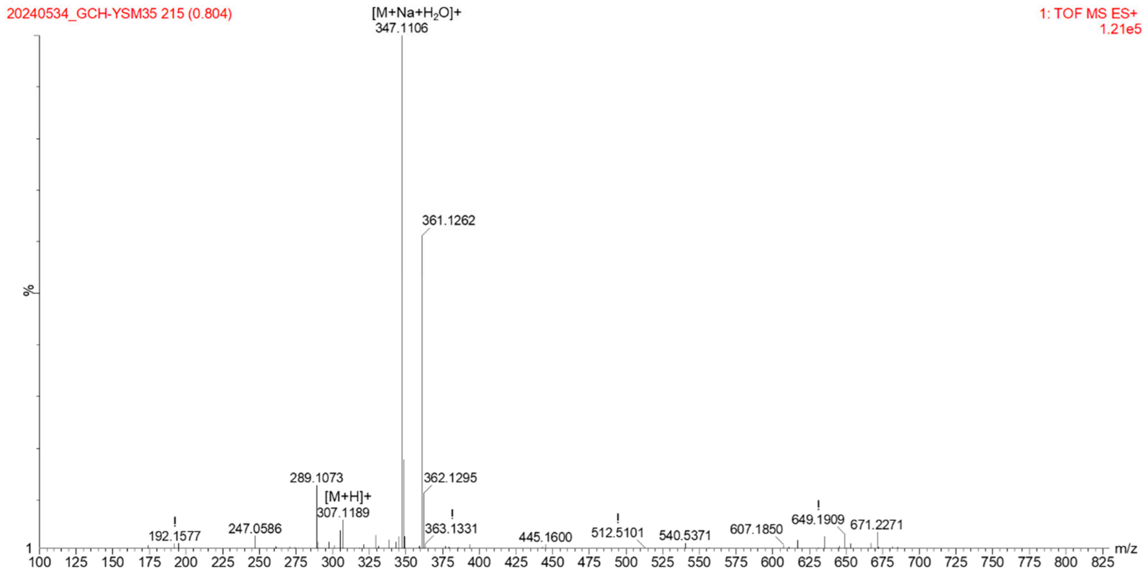

Figure S56 ^1H NMR spectrum of 9 in DMSO-d_6

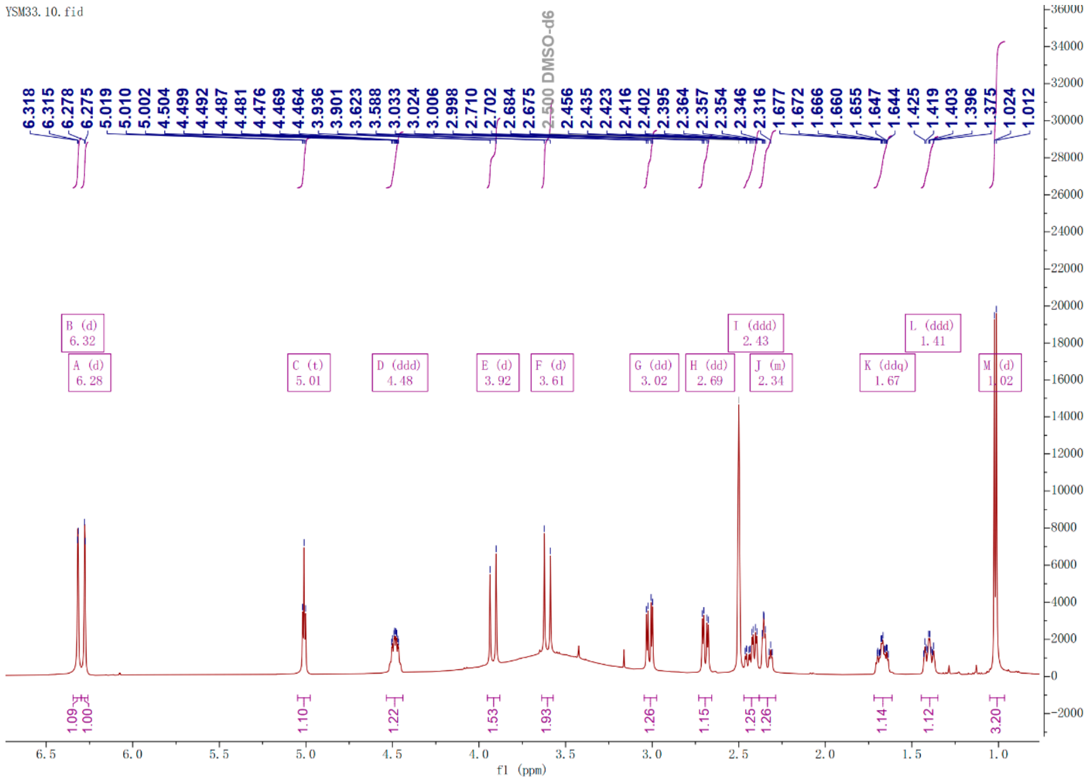

Figure S57 ^{13}C NMR spectrum of 9 in DMSO-d_6

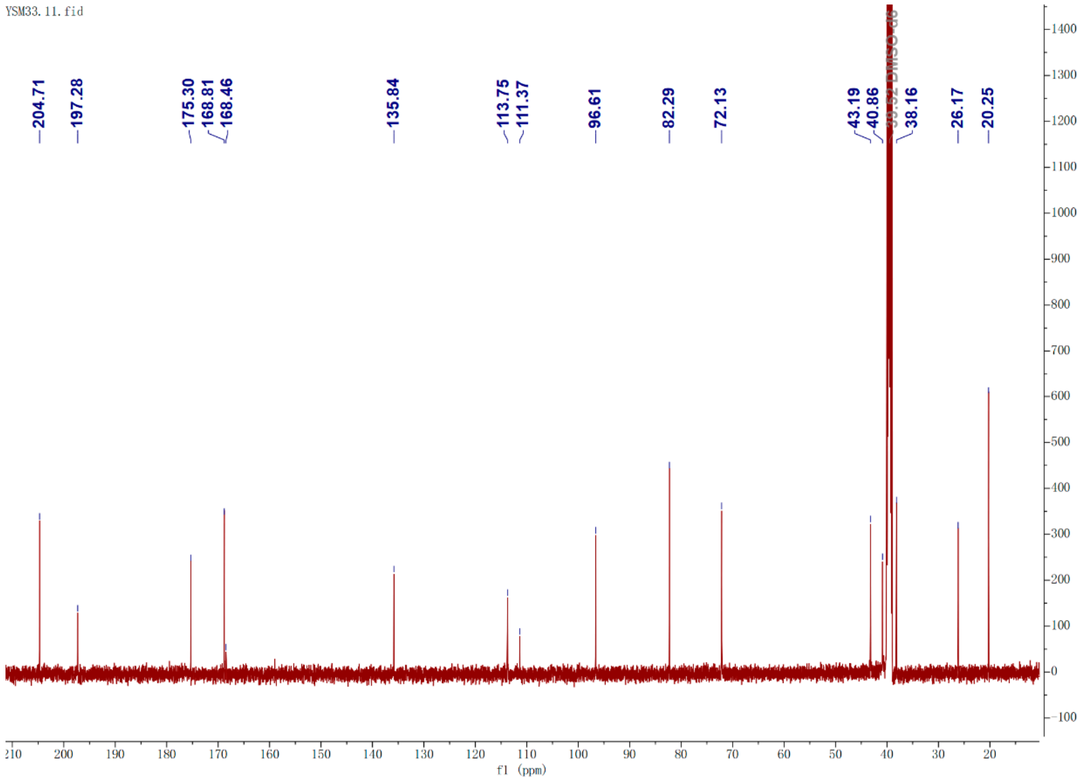

Figure S58 HR-ESIMS spectrum of 9

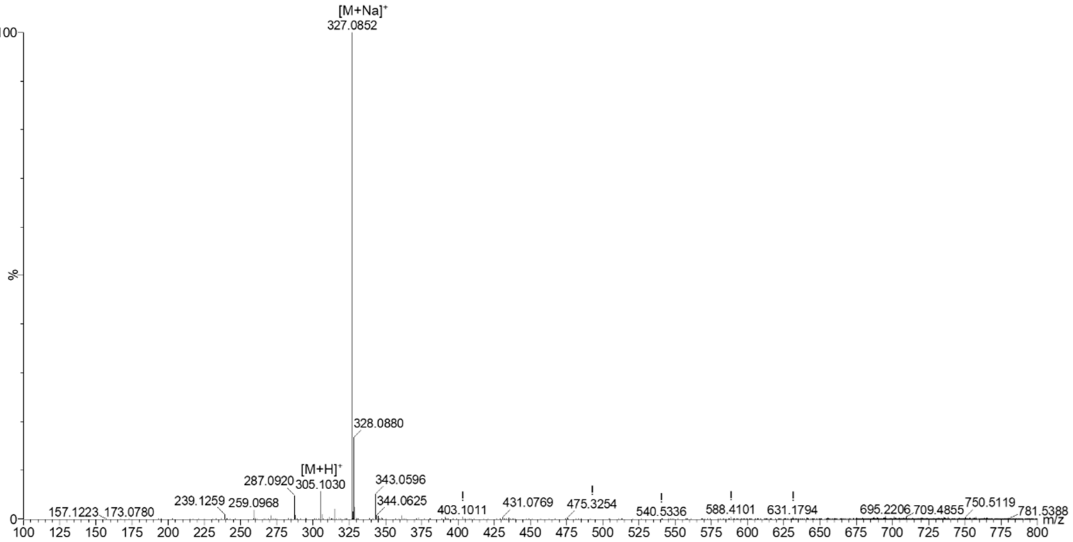

Figure S59 HPLC analysis spectra of compounds 5, 8-9

Compound **5**: white powder, t_R=21.30 min, purity: 95%

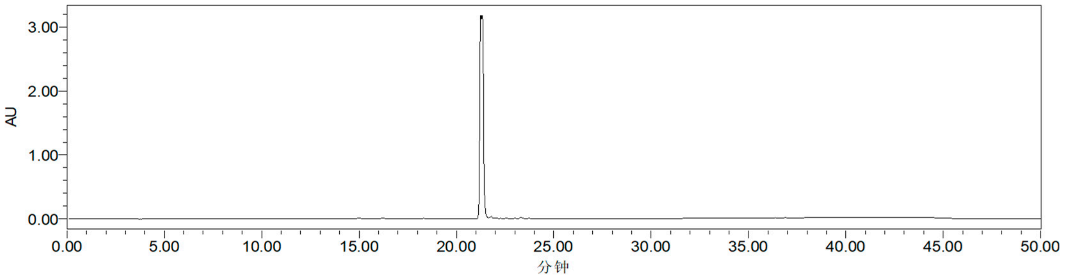

Compound **8**: yellow oil, t_R=21.64 min, purity: 93%

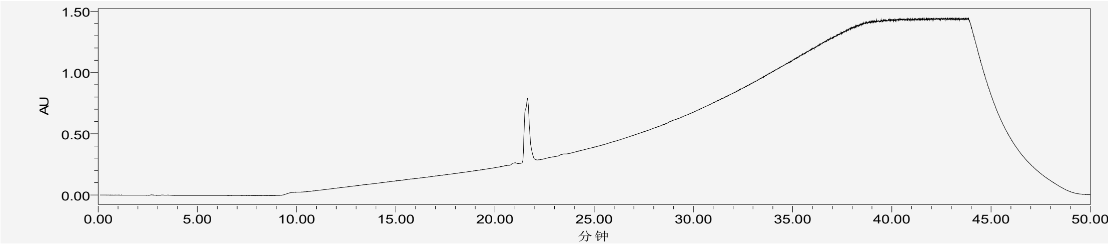

Compound **9**: white powder, t_R=24.28 min, purity: 94%

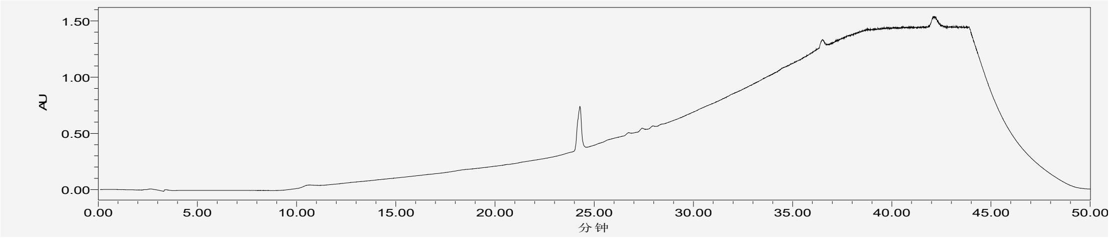

Figure S60 UV spectra of compounds 1-4, 6-7

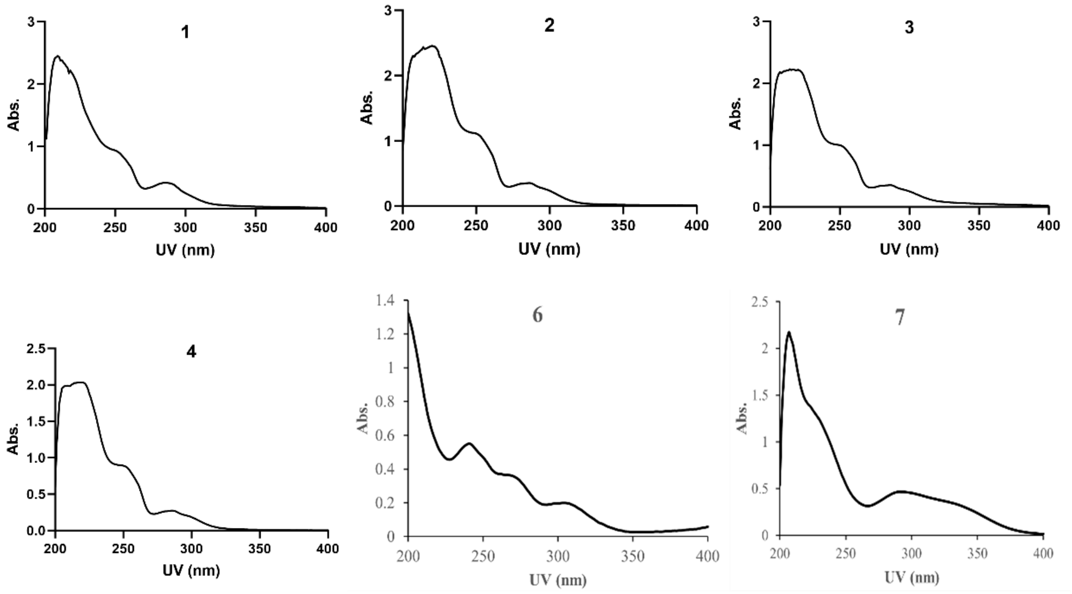

Figure S61 HPLC analysis of FDAA derivates of standard amino acids and compound 1

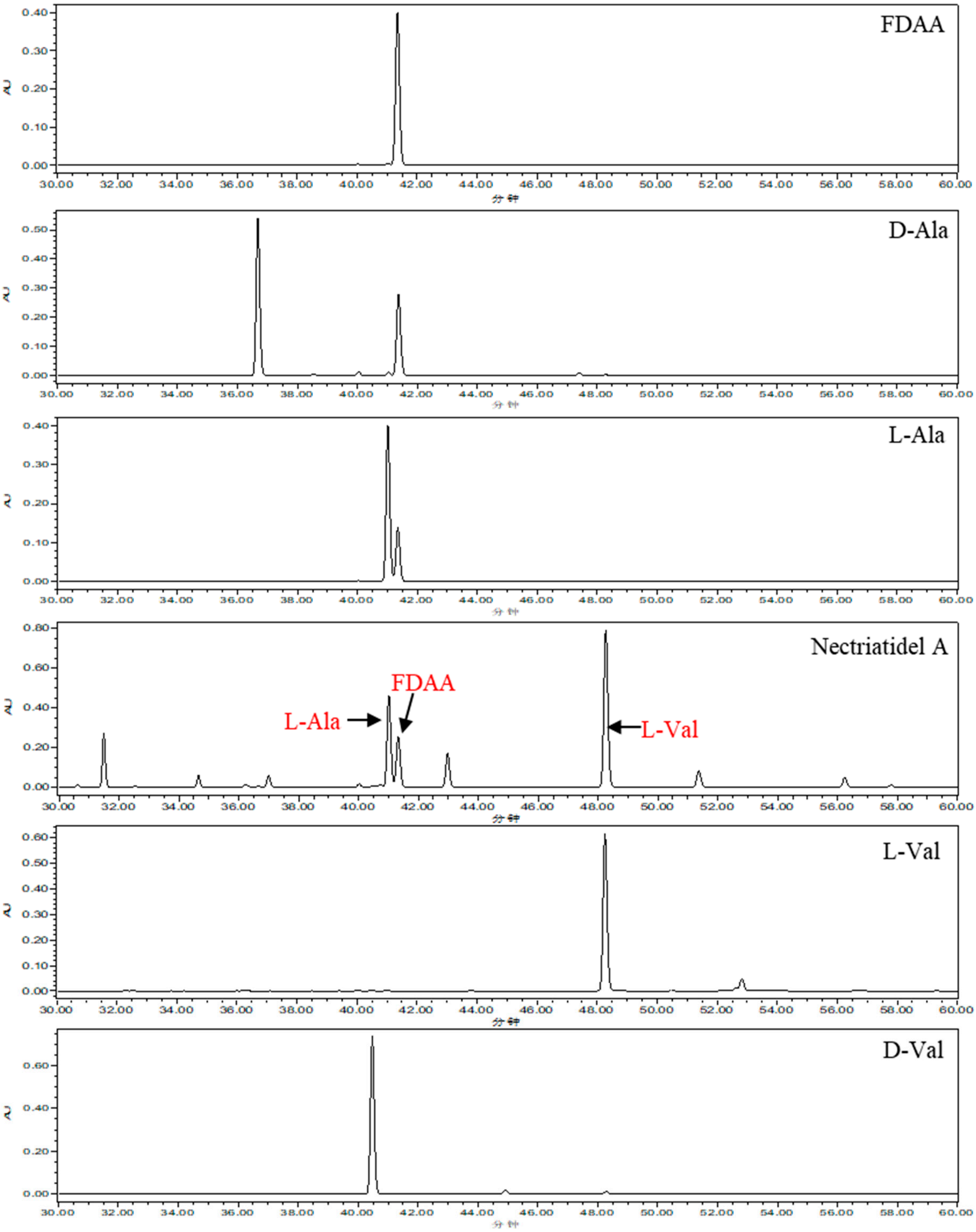

Figure S62 HPLC analysis of FDAA derivates of standard amino acids and compound 2

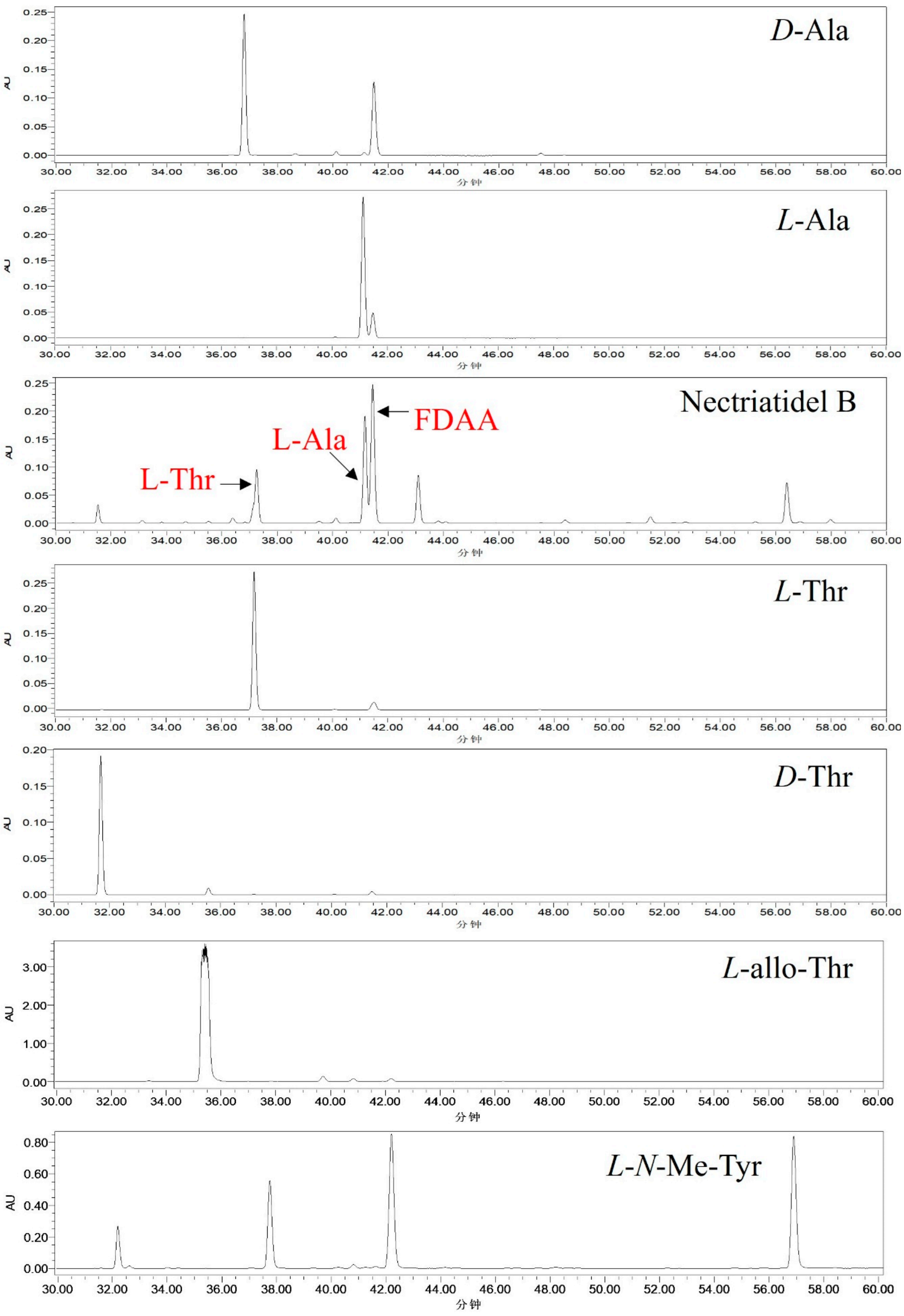

Figure S63 HPLC analysis of FDAA derivates of standard amino acids and compound 3

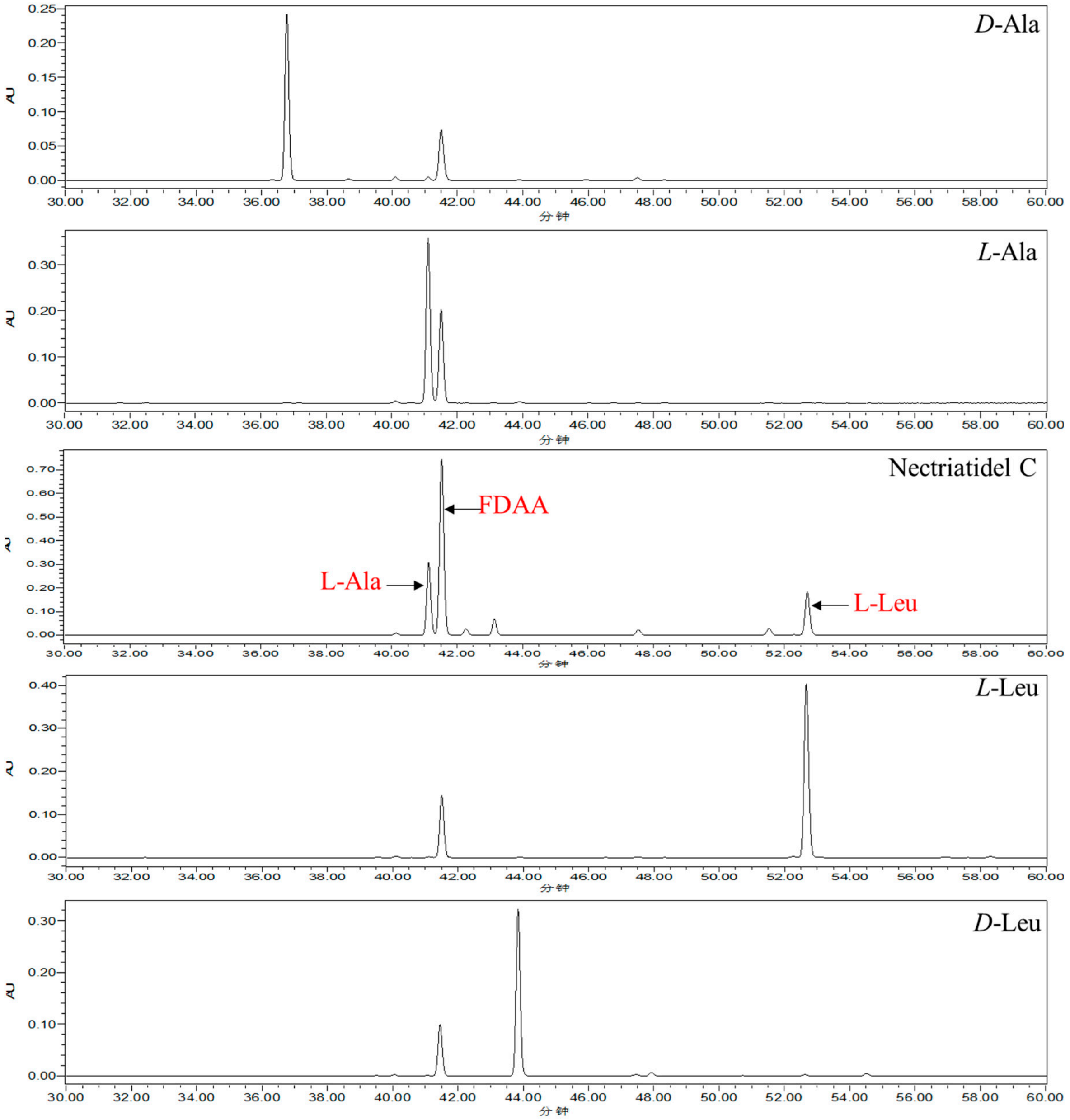

Figure S64 HPLC analysis of FDAA derivates of standard amino acids and compounds 4-5

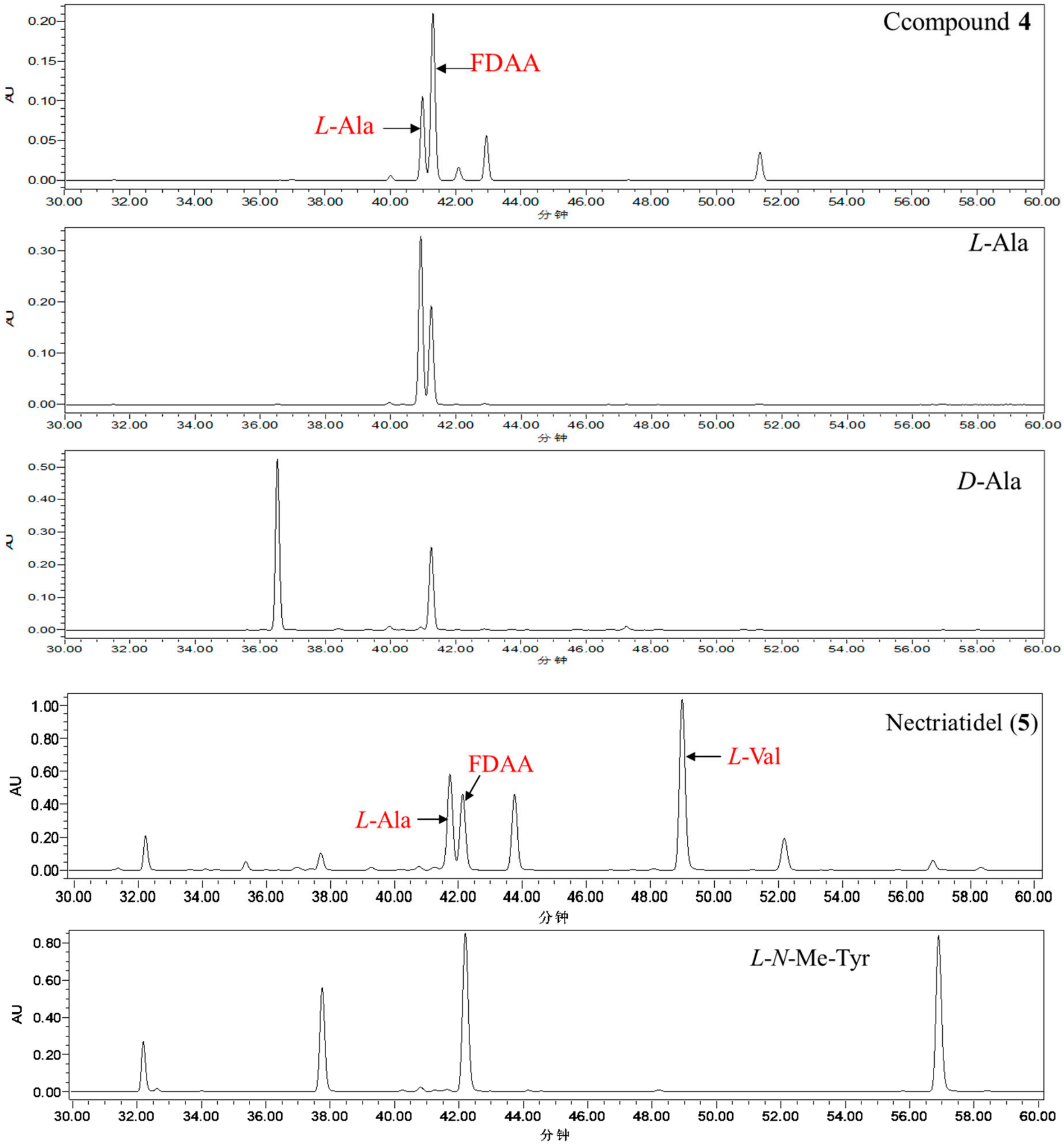

Figure S65 Conformations of low-energy conformers of 6 in MeOH

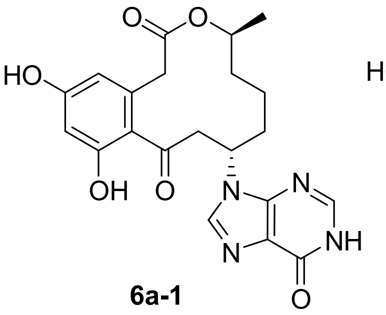

6a-1

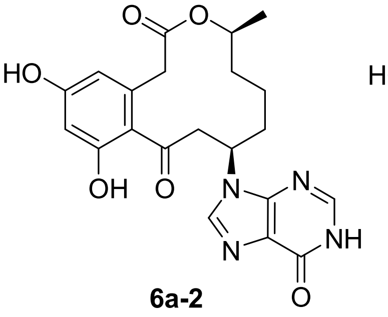

6a-2

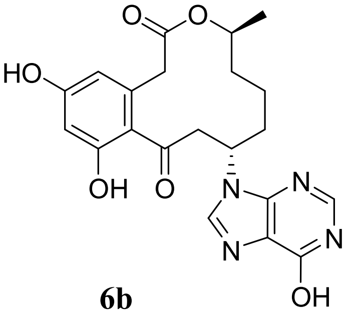

6b

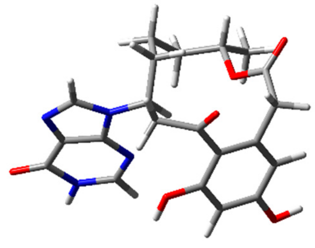

(11*S*,15*S*)-**6a**-1

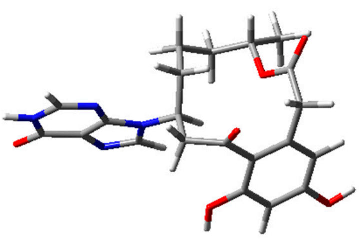

(11*S*,15*S*)-**6a**-2

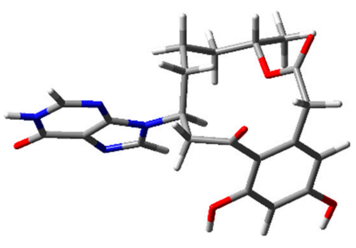

(11*S*,15*S*)-**6a**-3

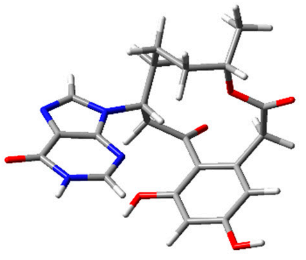

(11*R*,15*S*)-**6a**-1

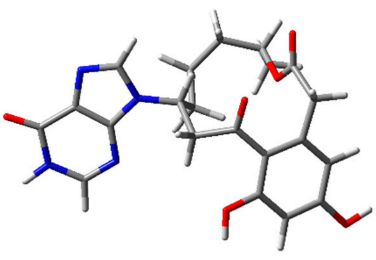

(11*R*,15*S*)-**6a**-2

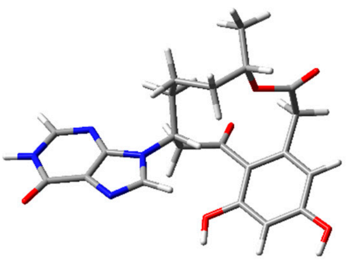

(11*R*,15*S*)-**6a**-3

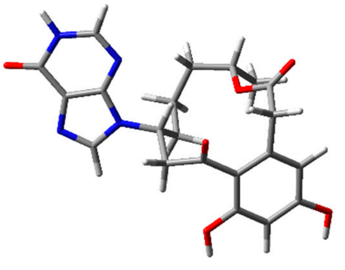

(11*R*,15*S*)-**6a**-4

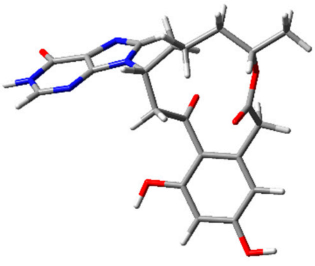

(11*R*,15*S*)-**6a**-5

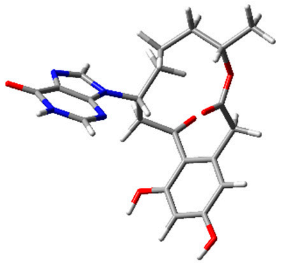

(11*R*,15*S*)-**6a**-6

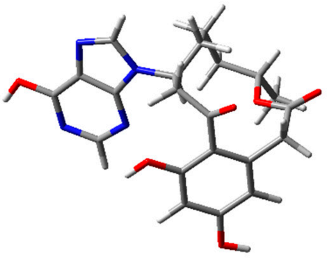

(11*S*,15*S*)-**6b**-1

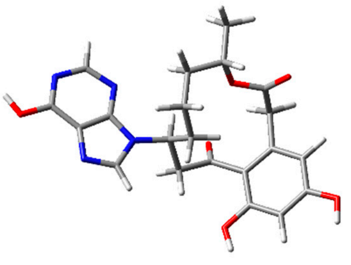

(11*S*,15*S*)-**6b**-2

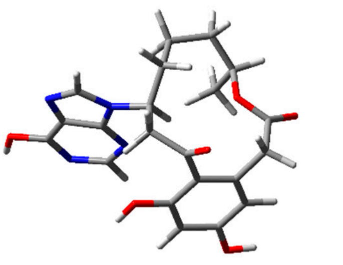

(11*S*,15*S*)-**6b**-3

Table S9 Relative thermal energies (ΔE), relative free energies (ΔG), and equilibrium populations (P) of low-energy conformers of 6 in MeOH

Conformers	G (kcal/mol)	ΔE(kcal/mol)[a]	Δ*G*(kcal/mol)[b]	P (%)[c]
(11*S*,15*S*)-**6a-1**	-931004.1910	0.0000	0.6221	21.90
(11*S*,15*S*)-**6a-2**	-931004.8131	0.8519	0.0000	62.62
(11*S*,15*S*)-**6a-3**	-931003.8827	1.0538	0.9305	13.01
Conformers	G (kcal/mol)	ΔE(kcal/mol)[a]	Δ*G*(kcal/mol)[b]	P (%)[c]
(11*R*,15*S*)-**6a-1**	-931002.7672	0.0000	0.0000	63.35
(11*R*,15*S*)-**6a-2**	-931000.5293	2.1362	2.2378	1.45
(11*R*,15*S*)-**6a-3**	-931001.5783	1.3080	1.1888	8.51
(11*R*,15*S*)-**6a-4**	-931002.0706	1.1069	0.6965	19.54
(11*R*,15*S*)-**6a-5**	-931000.5432	2.8483	2.2240	1.48
(11*R*,15*S*)-**6a-6**	-931000.9921	2.1591	1.7750	3.16
Conformers	G (kcal/mol)	ΔE(kcal/mol)[a]	Δ*G*(kcal/mol)[b]	P (%)[c]
(11*S*,15*S*)-**6b-1**	-930998.3155	0.0000	0.0000	96.41
(11*S*,15*S*)-**6b-2**	-930996.2138	1.5789	2.1017	1.94
(11*S*,15*S*)-**6b-3**	-930995.7358	2.2509	2.5797	1.65

[a] At the WB97XD/def2TZVP/ SMD level of theory. [b] From ΔG values at 298.15 K. [c] in MeOH, no imaginary frequency.

Figure S66 DP4+ analysis result of 6 (experimental for 6, isomer 1 for (11*S*,15*S*)-6a, isomer 2 for (11*S*,15*S*)-6b)

Functional		Solvent?		Basis Set		Type of Data	
mPW1PW91		PCM		6-311G(d,p)		Shielding Tensors	
		DP4+	100.00%	0.00%	–	–	–
Nuclei	sp2?	Experimental	Isomer 1	Isomer 2	Isomer 3	Isomer 4	Isomer 5
C	x	172.7	177.9	182.7			
C		41.1	42.0	43.2			
C	x	124.4	145.7	149.3			
C	x	113	113.9	116.7			
C	x	163	164.4	168.6			
C	x	103.1	103.6	107.1			
C	x	160.9	163.1	167.5			
C	x	119.1	120.3	124.3			
C	x	203.8	210.2	215.8			
C		41.1	56.8	59.4			
C		52.5	56.3	54.3			
C		33.3	33.22	35.88			
C		23.5	27.33	28.12			
C		32.4	32.51	33.92			
C		74.4	78.64	80.57			
C		21.5	22.03	22.49			
C	x	141.0	145.29	150.48			
C	x	125.0	128.62	125.46			
C	x	150.1	153.02	161.41			
C	x	146.4	149.36	160.17			
C	x	159.3	158.60	166.42			

Functional	Solvent?	Basis Set	Type of Data		
mPW1PW91	PCM	6-311G(d,p)	Shielding Tensors		

	Isomer 1	Isomer 2	Isomer 3	Isomer 4	Isomer 5	Isomer 6
sDP4+ (H data)	-	-	-	-	-	-
sDP4+ (C data)	99.94%	0.06%	-	-	-	-
sDP4+ (all data)	99.94%	0.06%	-	-	-	-
uDP4+ (H data)	-	-	-	-	-	-
uDP4+ (C data)	93.86%	6.14%	-	-	-	-
uDP4+ (all data)	93.86%	6.14%	-	-	-	-
DP4+ (H data)	-	-	-	-	-	-
DP4+ (C data)	100.00%	0.00%	-	-	-	-
DP4+ (all data)	100.00%	0.00%	-	-	-	-

Figure S67 DP4+ results and linear correlation plots between the experimental and calculated ^{13}C NMR chemical shifts of compound 6

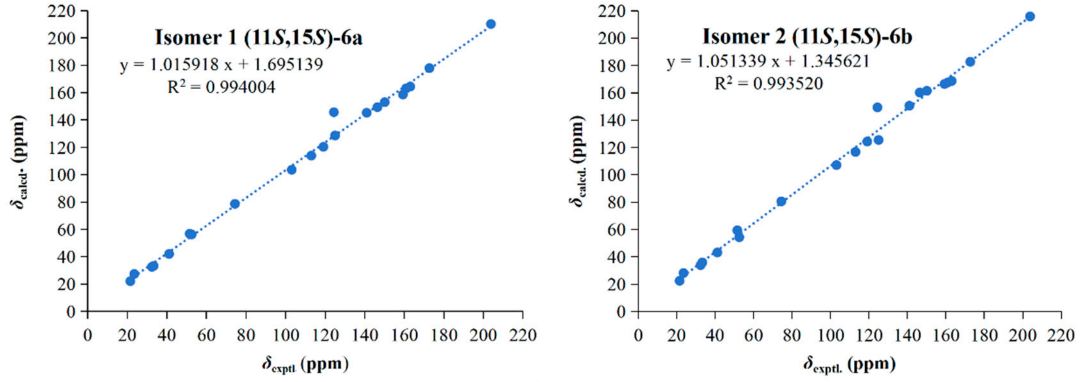

Figure S68 Conformations of low-energy conformers of 7 in MeOH

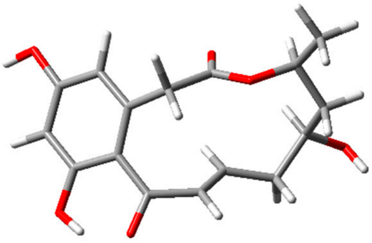
(13*S*,15*S*)-**7-1**

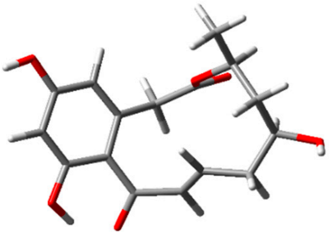
(13*S*,15*S*)-**7-2**

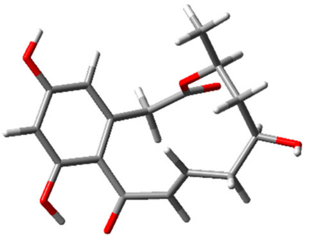
(13*S*,15*S*)-**7-3**

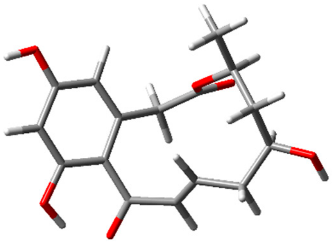
(13*S*,15*S*)-**7-4**

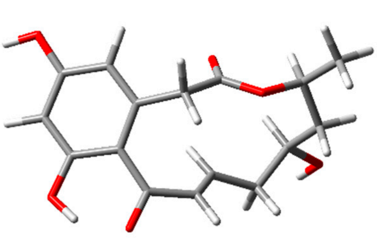
(13*S*,15*S*)-**7-5**

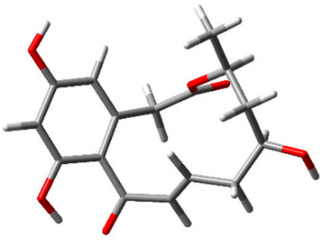
(13*S*,15*S*)-**7-6**

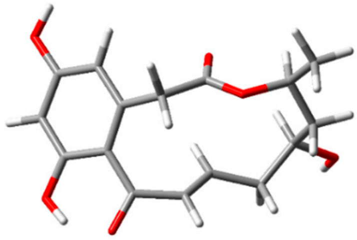

(13*S*,15*S*)-**7**-7

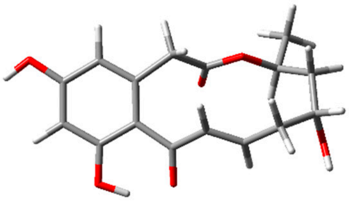

(13*R*,15*S*)-**7**-1

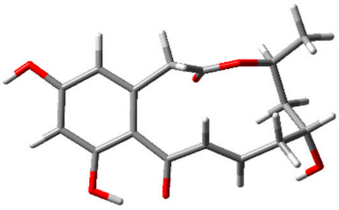

(13*R*,15*S*)-**7**-2

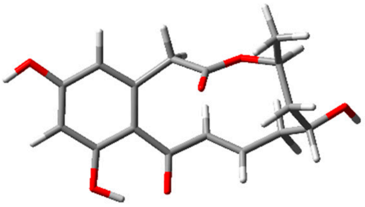

(13*R*,15*S*)-**7**-3

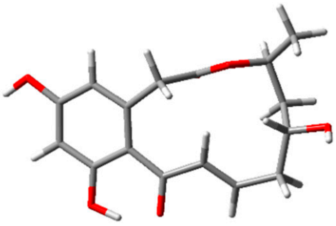

(13*R*,15*S*)-**7**-4

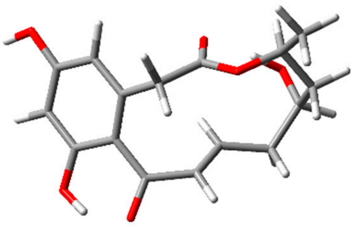

(13*R*,15*S*)-**7**-5

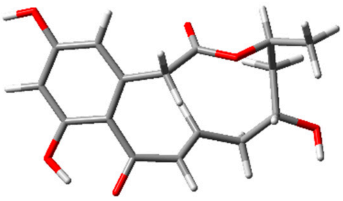

(13*R*,15*S*)-**7**-6

Table S10 Relative thermal energies (ΔE), relative free energies (ΔG), and equilibrium populations (P) of low-energy conformers of 7 in MeOH

Conformers	ΔE(kcal/mol)[a]	Δ*G*(kcal/mol)[b]	P (%)[c]
(13*S*,15*S*)-**7-1**	0.4261	0.4151	11.54
(13*S*,15*S*)-**7-2**	0.5159	0.6361	7.94
(13*S*,15*S*)-**7-3**	0.6165	0.7724	6.31
(13*S*,15*S*)-**7-4**	0.1554	0.2184	16.08
(13*S*,15*S*)-**7-5**	0.0003	0.0000	23.26
(13*S*,15*S*)-**7-6**	0.2602	0.3897	12.04
(13*S*,15*S*)-**7-7**	0.0000	0.0160	22.64
Conformers	ΔE(kcal/mol)[a]	Δ*G*(kcal/mol)[b]	P (%)[c]
(13*R*,15*S*)-**7-1**	0.0000	0.0000	63.88
(13*R*,15*S*)-**7-2**	0.4782	0.6934	19.80
(13*R*,15*S*)-**7-3**	1.5894	1.8052	3.03
(13*R*,15*S*)-**7-4**	1.6696	1.3125	6.96
(13*R*,15*S*)-**7-5**	1.4026	1.8519	2.80
(13*R*,15*S*)-**7-6**	2.4979	2.0661	1.95

[a] At the M06-2X/def2-TZVP/ SMD level of theory. [b] From ΔG values at 298.15 K. [c] in MeOH, no imaginary frequency.

Figure S69 DP4+ analysis result of 7 (experimental for 7, isomer 1 for (13*R*,15*S*)-7, isomer 2 for (13*S*,15*S*)-7)

Functional		Solvent?		Basis Set	
mPW1PW91		PCM		6-311G(d,p)	
		DP4+	0.00%	100.00%	-
Nuclei	sp2?	Experimental	Isomer 1	Isomer 2	Isomer 3
C	x	170.2	179.6	180.2	
C		40.1	48.8	47.0	
C	x	133.8	148.5	149.1	
C	x	109.6	115.5	117.2	
C	x	159.3	167.1	169.1	
C	x	101.5	104.2	104.8	
C	x	157.3	172.8	172.0	
C	x	118.0	117.2	119.7	
C	x	197.8	199.7	208.0	
C	x	133.8	138.3	141.2	
C	x	149.0	157.3	158.4	
C		43.0	45.24	51.71	
C		70.1	72.95	75.25	
C		45.7	43.61	49.99	
C		70.7	72.62	75.59	
C		21.3	21.70	23.12	

Functional	Solvent?	Basis Set	Type of Data			
mPW1PW91	PCM	6-311G(d,p)	Shielding Tensors			
	Isomer 1	Isomer 2	Isomer 3	Isomer 4	Isomer 5	Isomer 6
sDP4+ (H data)	-	-	-	-	-	-
sDP4+ (C data)	0.00%	100.00%	-	-	-	-
sDP4+ (all data)	0.00%	100.00%	-	-	-	-
uDP4+ (H data)	-	-	-	-	-	-
uDP4+ (C data)	29.15%	70.85%	-	-	-	-
uDP4+ (all data)	29.15%	70.85%	-	-	-	-
DP4+ (H data)	-	-	-	-	-	-
DP4+ (C data)	0.00%	100.00%	-	-	-	-
DP4+ (all data)	0.00%	100.00%	-	-	-	-

Figure S70 Conformations of low-energy conformers of 8 in MeOH

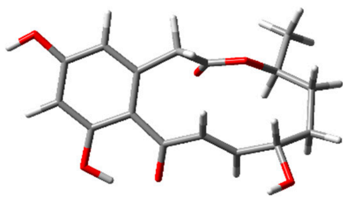

(12*S*,15*S*)-**8**-1

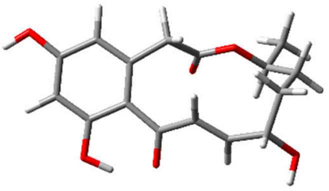

(12*S*,15*S*)-**8**-2

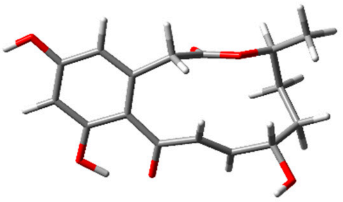

(12*S*,15*S*)-**8**-3

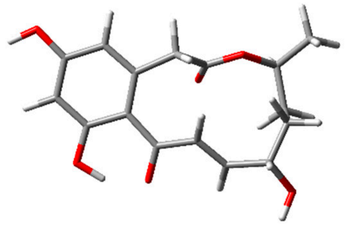

(12*S*,15*S*)-**8**-4

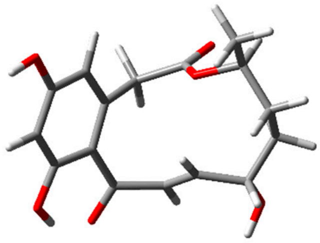

(12*S*,15*S*)-**8**-5

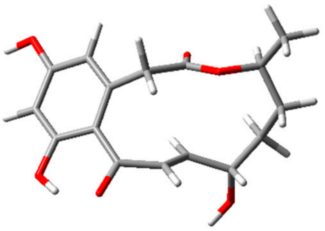

(12*S*,15*S*)-**8**-6

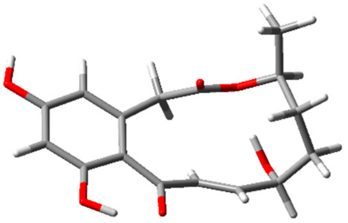

(12*S*,15*S*)-**8**-7

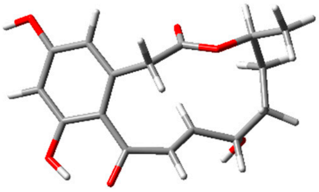

(12*S*,15*S*)-**8**-8

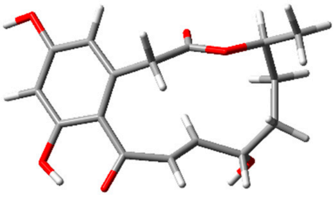

(12*S*,15*S*)-**8**-9

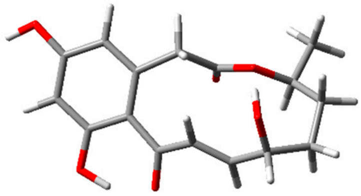

(12*R*,15*S*)-**8**-1

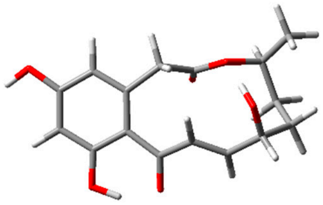

(12*R*,15*S*)-**8**-2

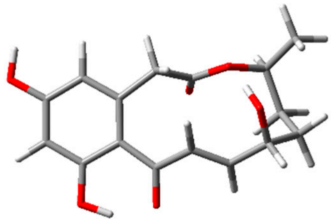

(12*R*,15*S*)-**8**-3

Figure S71 Comparison of the experimental and calculated ECD spectra of 8

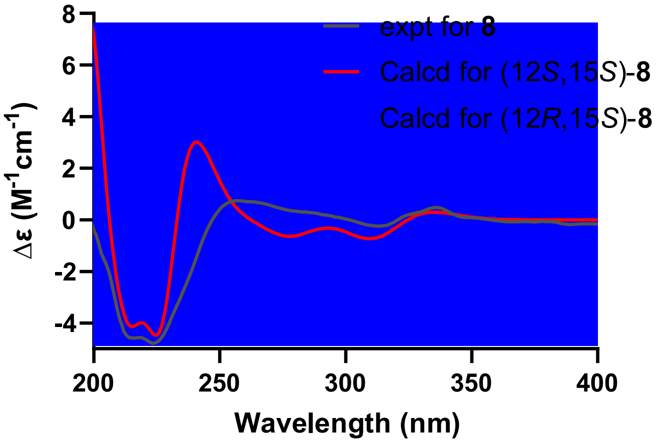

Table S11 Relative thermal energies (ΔE), relative free energies (ΔG), and equilibrium populations (P) of low-energy conformers of 8 in MeOH

Conformers	ΔE(kcal/mol)[a]	Δ*G*(kcal/mol)[b]	P (%)[c]
(12*S*,15*S*)-**8-1**	0.3092	0.0000	33.36
(12*S*,15*S*)-**8-2**	0.4565	0.3788	17.60
(12*S*,15*S*)-**8-3**	0.0000	0.0083	32.90
(12*S*,15*S*)-**8-4**	1.7322	1.3176	3.60
(12*S*,15*S*)-**8-5**	1.7648	1.2203	4.25
(12*S*,15*S*)-**8-6**	2.4494	1.7266	1.81
(12*S*,15*S*)-**8-7**	2.6994	1.5007	2.65
(12*S*,15*S*)-**8-8**	3.5676	2.0555	1.04
(12*S*,15*S*)-**8-9**	3.3494	2.0459	1.05
Conformers	ΔE(kcal/mol)[a]	Δ*G*(kcal/mol)[b]	P (%)[c]
(12*R*,15*S*)-**8-1**	0.3694	0.0000	42.34
(12*R*,15*S*)-**8-2**	0.0000	0.1483	32.96
(12*R*,15*S*)-**8-3**	0.1987	0.3859	22.06

Figure S72 DP4+ analysis result of 8 (experimental for 8, isomer 1 for (12*R*,15*S*)-8, isomer 2 for (12*S*,15*S*)-8)

Functional	Solvent?	Basis Set	Type of Data
mPW1PW91	PCM	6-311G(d, p)	Shielding Tensors

		DP4+	90. 68%	9. 32%	-	-	-
Nuclei	sp2?	Experimental	Isomer 1	Isomer 2	Isomer 3	Isomer 4	Isomer 5
C	x	170. 2	176. 9	178. 8			
C		39	48. 7	48. 6			
C	x	132. 5	146. 5	148. 3			
C	x	108. 6	114. 8	115. 6			
C	x	158. 4	165. 6	167. 1			
C	x	101. 4	103. 5	104. 1			
C	x	155. 6	171. 5	172. 8			
C	x	119. 1	115. 8	117. 0			
C	x	198. 4	197. 2	199. 6			
C	x	129. 8	132. 1	136. 8			
C	x	156. 7	162. 4	160. 3			
C		68. 2	74. 64	76. 59			
C		32. 2	35. 80	36. 23			
C		26. 9	31. 24	32. 67			
C		72. 4	77. 09	77. 14			
C		20. 5	20. 05	21. 01			

Functional	Solvent?	Basis Set	Type of Data		
mPW1PW91	PCM	6-311G(d,p)	Shielding Tensors		

	Isomer 1	Isomer 2	Isomer 3	Isomer 4	Isomer 5	Isomer 6
sDP4+ (H data)	-	-	-	-	-	-
sDP4+ (C data)	86.20%	13.80%	-	-	-	-
sDP4+ (all data)	86.20%	13.80%	-	-	-	-
uDP4+ (H data)	-	-	-	-	-	-
uDP4+ (C data)	60.91%	39.09%	-	-	-	-
uDP4+ (all data)	60.91%	39.09%	-	-	-	-
DP4+ (H data)	-	-	-	-	-	-
DP4+ (C data)	90.68%	9.32%	-	-	-	-
DP4+ (all data)	90.68%	9.32%	-	-	-	-

Figure S73 DP4+ results and linear correlation plots between the experimental and calculated ^{13}C NMR chemical shifts of compounds 7-8

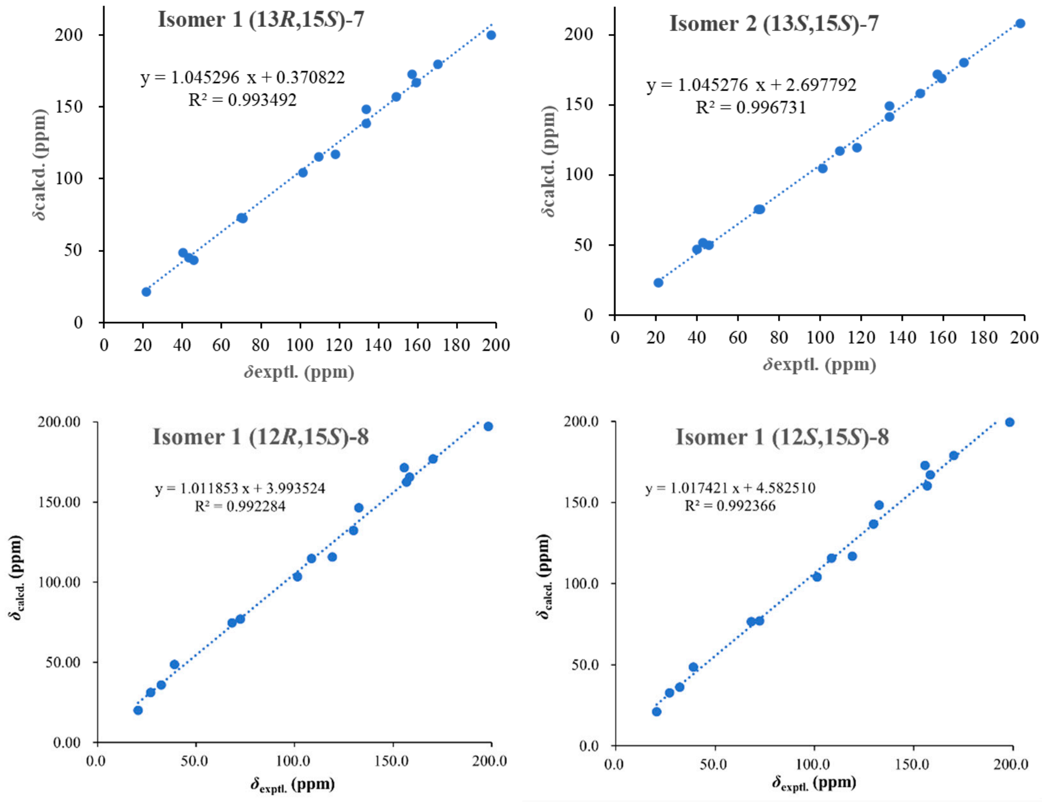

Table S12 Line correlation coefficients R^2 and mean absolute error (MAE) analyses of the experimental and calculated ^{13}C NMR data of model compounds 6-8

Model compounds	(11*S*,15*S*)-**6a**	(11*S*,15*S*)-**6b**	(11*R*,15*S*)-**7**	(11*S*,15*S*)-**7**	(11*S*,15*S*)-**8**	(11*R*,15*S*)-**8**
R^2	0.994004	0.993520	0.993492	0.996731	0.992366	0.992284
MAE	3.445874	6.753342	5.609585	7.568355	6.690468	5.867718

Figure S74 The anti-*Mycobacterium tuberculosis* activity of compounds 7-8

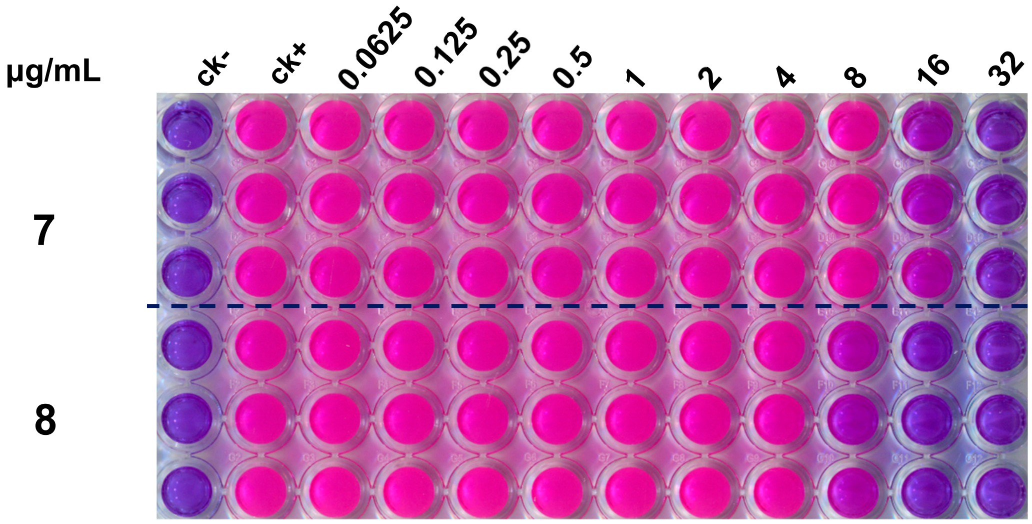

Figure S75 The cytotoxicity of compounds 1-5, 7-9 against cell lines SW480, B16F10, DLD-1, PC-3 and 22Rv1

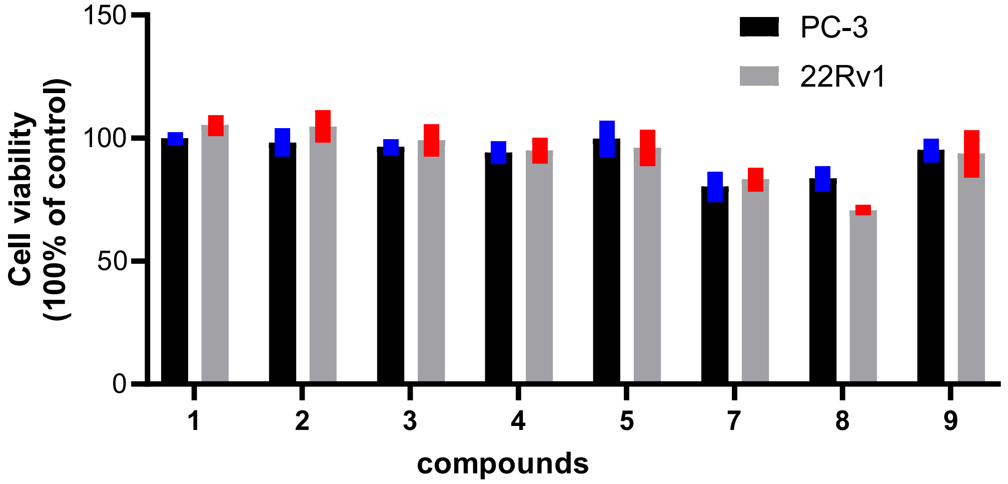

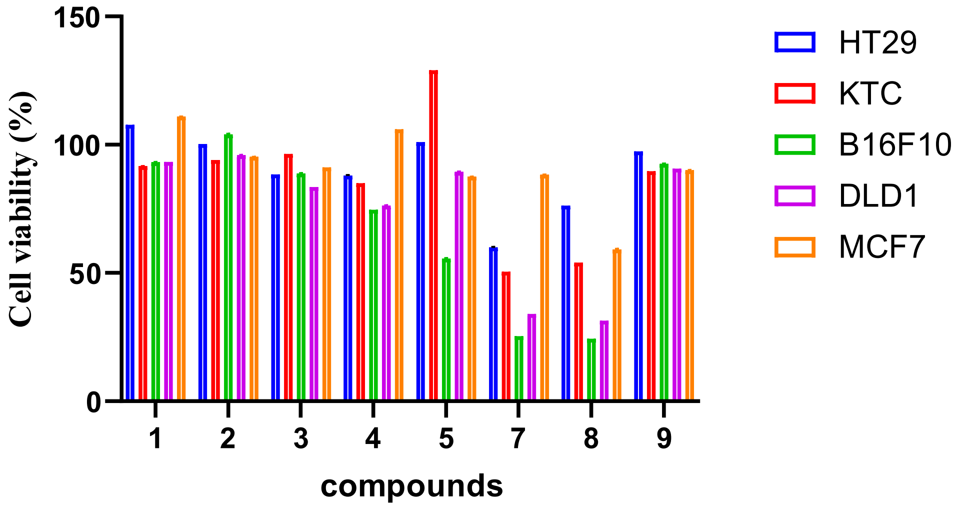

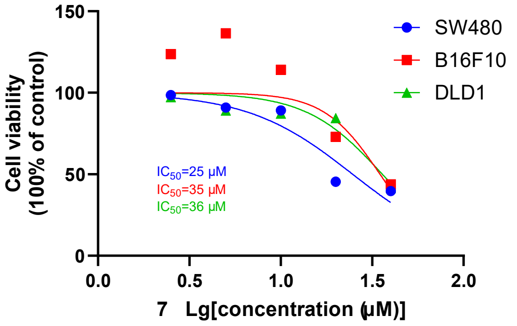

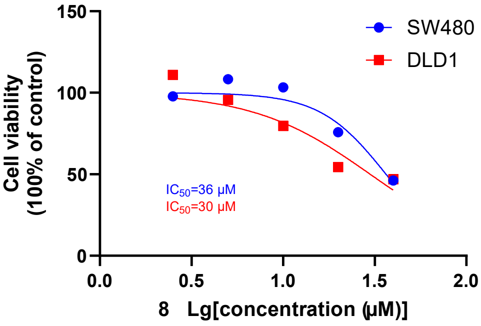

Table S13 The acetylcholinesterase and *α*-glucosidase activities of compounds 1-5, 7-9

No.	Inhibition rate	
	α-glucosidase	AChE
**1**	28.03%	-3.20%
**2**	24.09%	-3.44%
**3**	32.12%	-1.15%
**4**	26.65%	1.10%
**5**	25.34%	-5.65%
**7**	33.90%	-12.00%
**8**	27.01%	14.86%
**9**	26.45%	2.70%
acarbose	100.14%	-
tacrine	-	100.08%

Notes: acetylcholinesterase (AChE), - means no test.

Figure S76 Organization of three NPRS biosynthetic gene clusters

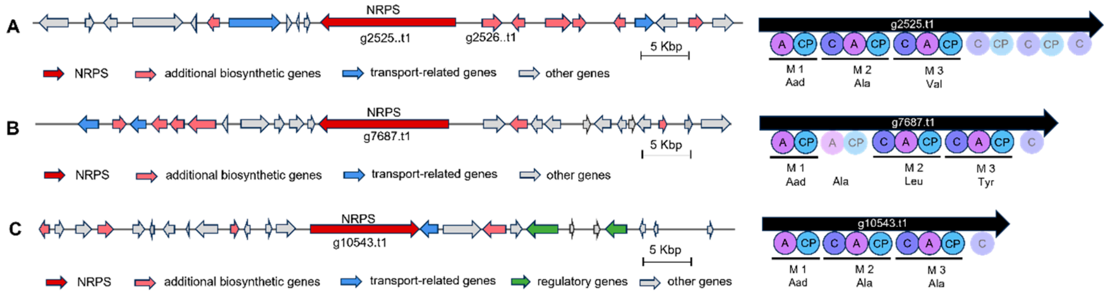

Figure S77 The proposed NRPS biosynthetic pathway for 5

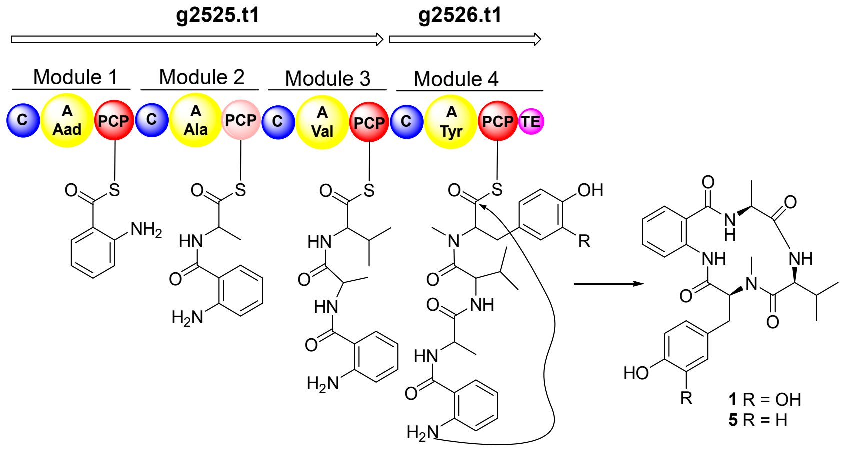

Figure S78 Structure and arrangement of genes involved in curvularin biosynthesis in the *Aspergillus spelaeus* genome.

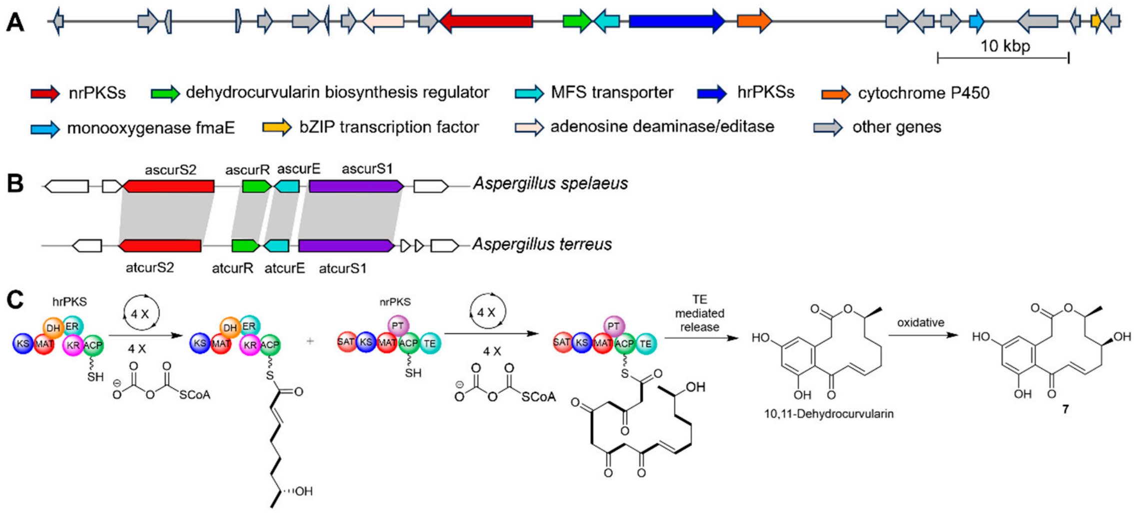

Table S14 Genome features of *Aspergillus spelaeus* GXIMD 04541

Category	Property
Genome Size (bp)	35909697
Number of scaffolds	358
GC content (%)	52.6
CDS Number	11886
tRNA genes	199
rRNA genes	44
Genes of COG	5277
Genes of KEGG	8125
BUSCO completeness (%)	98.5
BUSCO duplication (%)	0.1
CEGMA completeness (%)	94.76
Scaffold N50 (bp)	1176687
Scaffold N90 (bp)	301426
Tandem repeat sequences (bp)	773635
Pfam	9129
Notes from Swiss-Prot	8768
BGC of Secondary Metabolites	106

Table S15 Secondary metabolite biosynthesis gene clusters in *Aspergillus spelaeus* GXIMD 04541

No.	Type	From	To	Most similar known cluster	Similarity Confidence
1	T1PKS,other,indole,terpene	1	70,007	citreoviridin	Medium
2	T1PKS	735,190	804,879		
3	terpene	960,037	993,190		
4	NRPS,indole	1,439,907	1,509,029		
5	T1PKS	2,051,942	2,134,012	dehydrocurvularin	Low
6	NRPS,T1PKS,indole	254,952	349,380	dichlorodiaporthin	Medium
7	T1PKS	622,645	690,273	shimalactone A/shimalactone B	Low
8	isocyanide,indole	1,006,936	1,067,999		
9	isocyanide,terpene	1,502,716	1,578,800	14-(*N,N*-dimethylleucyloxy)paspalinine/14-(leucyloxy)paspalinine/14-hydroxypaspalinine	Low
10	T1PKS	1,670,180	1,739,553		
11	T1PKS,NRPS	1,902,858	2,007,358		
12	NRPS-like	6,524	69,493	atpenin B	Low
13	NRPS,indole	114,078	187,650	penigequinolone A	High
14	terpene	797,627	830,871		
15	NRPS-like,terpene	974,969	1,038,283		
16	terpene	1,592,926	1,624,463		
17	NRPS-like	1	61,116		
18	indole	159,424	190,768	ochratoxin A	High
19	NRPS	374,218	448,510	metachelin C/metachelin A/metachelin A-CE/metachelin B/dimerumic acid 11-mannoside/dimerumic acid	Low
20	T1PKS	689,098	758,873		
21	betalactone	930,700	969,779		
22	indole	1,446,537	1,477,861		
23	terpene,NRPS,terpene-precursor	1,635,505	1,729,792		
24	NRPS	1,767,807	1,831,977		
25	NRPS-like,T1PKS,indole,terpene	14,744	101,693		
26	indole	254,280	290,739	communesin A/communesin B/communesin C/communesin D/communesin E/communesin G/communesin H	Low

27	NRPS	445,871	524,631	aspercryptins	Low
28	NRPS-like,terpene	849,563	912,811		
29	NRPS-like	913,247	976,509		
30	terpene-precursor	1,259,973	1,291,147		
31	NRPS-like	1,463,279	1,523,341		
32	terpene	95,882	127,519		
33	terpene	252,269	284,751	clavaric acid	High
34	NI-siderophore	407,772	451,776		
35	T1PKS	790,315	858,241		
36	terpene	1,242,326	1,273,392		
37	NRPS	1	42,014		
38	terpene	776,626	808,206		
39	terpene-precursor	368,829	400,028		
40	T3PKS	1,136,948	1,198,261		
41	NRPS-like	124,611	188,404		
42	terpene	376,323	407,873		
43	indole	414,396	447,831		
44	terpene,T1PKS	541,120	620,836		
45	T1PKS,NRPS-like	675,208	783,738		
46	NRPS	1,066,312	1,196,970	asperphenamate	Low
47	indole	1,249,141	1,280,510		
48	NRPS	791	85,966		
49	NRPS-like,terpene	109,595	173,834		
50	NRPS-like,NRPS,T1PKS,indole	769,144	923,235	HEx-pks23 polyketide	Low
51	NRPS-like,indole	343,247	406,009		
52	NRPS-like	836,088	898,862	aspulvinone H/aspulvinone B1	High
53	NRPS-like,NRPS,T1PKS	1	142,240		
54	terpene	362,383	393,666		
55	terpene-precursor	403,753	434,882		
56	NRPS	443,961	516,375		
57	NI-siderophore	709,942	752,993		
58	terpene	813,400	845,454		
59	T1PKS,terpene	1,006,582	1,084,569	PR-toxin	Low
60	NRPS-like	110,359	174,183	choline	High
61	T1PKS	211,357	279,859		
62	terpene	534,371	565,968		
63	NRPS-like,T1PKS	677,141	748,146		
64	NRPS	838,430	878,016		
65	NRPS	416,880	490,623		
66	NRPS-like	740,866	803,601	asterriquinone CT5	Low
67	T1PKS,NRPS-like	168,110	286,745		

68	terpene	299,798	330,225		
69	NRPS	532,021	610,074	nidulanin A	Low
70	T1PKS	402,647	470,548		
71	terpene	707,300	738,274		
72	NRPS	9,116	72,409		
73	T1PKS,NRPS,betalactone	117,544	244,964	penicillin	Low
74	NRPS,T1PKS	154,335	226,663	flavichalasine F/flavichalasine G/aspochalasin C/aspochalasin E/aspochalasin M/TMC-169/aspergillin PZ	High
75	T1PKS	241,185	307,875	endocrocin	Low
76	terpene,NRPS	154,102	239,011		
77	T1PKS	295,916	363,494		
78	terpene	513,048	544,365		
79	T1PKS	40,324	109,294		
80	NRPS-like	344,004	407,141		
81	terpene	58,566	90,165	squalestatin S1	Medium
82	indole	133,361	164,473		
83	terpene	255,993	287,488		
84	T1PKS,terpene	558,707	624,068	chevalone E	High
85	NRPS-like	168,156	230,932		
86	terpene	475,956	506,733		
87	terpene	171,453	204,028		
88	indole	512,531	543,366		
89	terpene	1	28,637		
90	T1PKS,terpene	196,545	262,465	YWA1	High
91	terpene	394,511	426,938	clavaric acid	High
92	T1PKS	175,363	319,447		
93	NRPS	1	66,473		
94	terpene	215,480	246,409		
95	terpene	65,280	99,011		
96	fungal_CDPS	287,856	319,705		
97	T1PKS	1	67,486		
98	NRPS,terpene	88,699	152,118		
99	NRPS-like	219,189	282,499		
100	NRPS-like	342,379	382,138		
101	NRPS,indole	171,545	208,053		
102	NRPS	1	39,348		
103	T1PKS	7,779	75,985		
104	terpene	27,000	57,979		
105	NRPS-like,indole	1	92,429	terrequinone A	High
106	T1PKS	1	49,933		
